# Supplementary material for: Synthesis and Biological Activities of C1-Substituted Acylhydrazone β-Carboline Analogues as Antifungal Candidates
Source: Molecules. 2024 Jul 29;29(15):3569. doi: 10.3390/molecules29153569 (PMC11314034; doi:10.3390/molecules29153569)
Supplement: Supplementary file 1 [file molecules-29-03569-s001.zip › molecules-3111336-supplementary.pdf]

Supplementary Material

Synthesis and biological activities of C1-substituted acylhydrazone *β*-carboline analogues as antifungal candidates

Yujie Xu [1], Lishan Li [1], Jinghan Zhang [1], Yu Lan [1], Na Li [2,*] and Junru Wang [1,*]

[1] College of Chemistry and Pharmacy, Northwest A&F University, Yangling, 712100, China.
xuyj91@nwafu.edu.cn (Y.J.X.); 2021051625@nwafu.edu.cn (L.H.L.); zhangjinghan0906@163.com (J.H.Z.); lanyu9920@nwafu.edu.cn (Y.L.)

[2] Department of Scientific Research Services, Sanya Yazhou Bay Center for Innocation and Development Co., Ltd., Sanya, 572000, China

* Correspondence: wangjunru@nwafu.edu.cn (J.R.W.); Na Li, yl0lina@163.com (N.L.)

1. Data for compounds 9c-o and 10c-o.

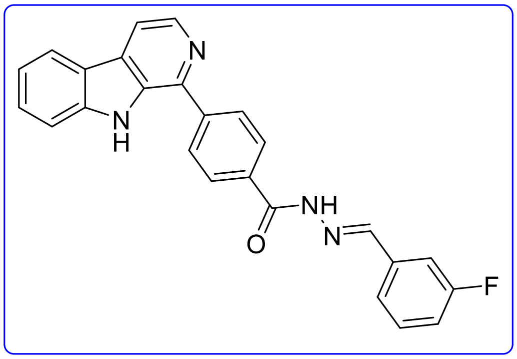

Data for **9c**: white solid, yield: 41%; ^{1}H NMR (600 MHz, DMSO-d_6) δ: 12.14 (s, 1H, H-9), 11.63 (s, 1H), 8.54 (s, 1H), 8.51 (d, J = 4.8 Hz, 1H), 8.21-8.17 (m, 5H, -Ph), 7.67 (d, J = 8.4 Hz, 1H), 7.61-7.53 (m, 4H), 7.30-7.27 (m, 2H); ^{13}C NMR (150 MHz, DMSO-d_6) δ: 163.7, 163.3, 147.1, 142.1, 141.6, 141.5, 139.0, 133.7, 133.3, 131.5, 130.0, 130.0, 128.9, 128.6, 124.0, 122.2, 121.2, 120.1, 117.4 (J_{CF} = 21.0 Hz), 115.0, 113.5 (J_{CF} = 22.5 Hz), 112.9; MS (ESI) m/z calcd for $C_{25}H_{17}FN_4O$ ([M+H]$^+$), 409.1459; found, 409.1472. The coupling between F and C1 was not detected in ^{13}C NMR spectrum..

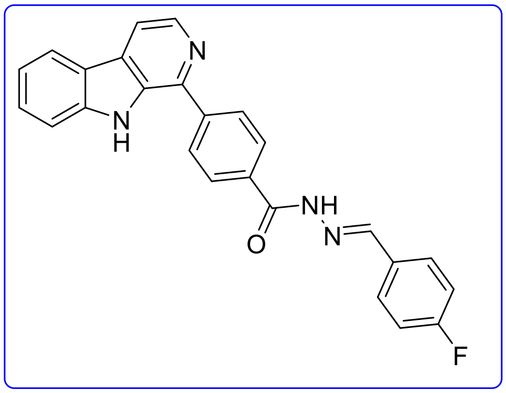

Data for **9d**: white solid, yield: 44%; ^{1}H NMR (600 MHz, DMSO-d_6) δ: 12.08 (s, 1H, H-9), 11.65 (s, 1H), 8.56 (s, 1H), 8.51 (d, J = 5.4 Hz, 1H), 8.30 (d, J = 7.8 Hz, 1H), 8.19-8.18 (m, 5H, -Ph), 7.85-7.83 (m, 2H), 7.67 (d, J = 7.8 Hz, 1H), 7.59-7.57 (m, 1H), 7.35-7.32 (m, 1H), 7.30-7.28 (m, 1H); ^{13}C

NMR (150 MHz, DMSO-d_6) δ: 164.5, 163.2, 147.3, 142.0, 141.6, 141.5, 139.0, 133.7, 133.4, 131.5, 130.0, 129.8, 128.9, 128.8, 128.5, 122.2, 121.2, 120.1, 116.4 (J_{CF} = 21.0 Hz), 114.9, 112.9; MS (ESI) m/z calcd for $C_{25}H_{17}FN_4O$ ($[M+H]^+$), 409.1459; found, 409.1466. The coupling between F and C1 was not detected in ^{13}C NMR spectrum.

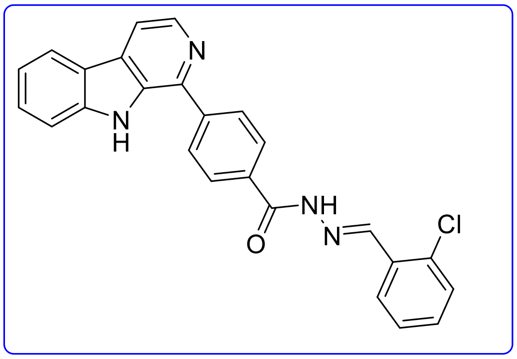

Data for **9e**: white solid, yield: 55%; ^1H NMR (600 MHz, DMSO-d_6) δ: 12.29 (s, 1H, H-9), 11.66 (s, 1H), 8.97 (s, 1H), 8.51 (d, J = 5.4 Hz, 1H), 8.30 (d, J = 7.8 Hz, 1H), 8.20-8.19 (m, 5H, -Ph), 8.09-8.07 (m, 1H), 7.67 (d, J = 8.4 Hz, 1H), 7.58-7.57 (m, 2H), 7.49-7.47 (m, 2H), 7.30-7.28 (m, 1H); ^{13}C NMR (150 MHz, DMSO-d_6) δ: 162.2, 143.2, 141.0, 140.6, 140.4, 137.9, 132.6, 132.1, 131.0, 129.4, 128.9, 127.9, 127.8, 127.5, 127.1, 126.3, 120.2, 119.1, 113.9, 111.8; MS (ESI) m/z calcd for $C_{25}H_{17}ClN_4O$ ($[M+H]^+$), 425.1164; found, 425.1173.

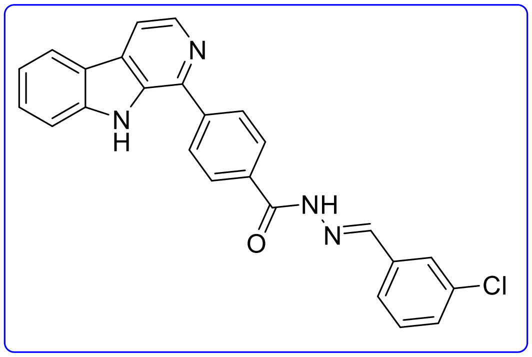

Data for **9f**: white solid, yield: 73%; ^1H NMR (600 MHz, DMSO-d_6) δ: 12.18 (s, 1H, H-9), 11.64 (s, 1H), 8.52-8.50 (m, 2H), 8.29 (d, J = 7.8 Hz, 1H), 8.19-8.18 (m, 5H, -Ph), 7.83 (s, 1H), 7.73 (s, 1H),

7.67 (d, J = 8.4 Hz, 1H), 7.59-7.56 (m, 1H), 7.52 (d, J = 4.2 Hz, 2H), 7.30-7.27 (m, 1H); ^{13}C NMR (150 MHz, DMSO-d_6) δ: 163.4, 146.7, 142.1, 141.6, 141.5, 139.0, 137.1, 134.2, 133.7, 131.3, 130.0, 128.9, 128.8, 128.6, 126.8, 126.3, 122.2, 121.2, 120.1, 115.0, 112.9; MS (ESI) m/z calcd for $C_{25}H_{17}ClN_4O$ ([M+H]$^+$), 425.1164; found, 425.1174.

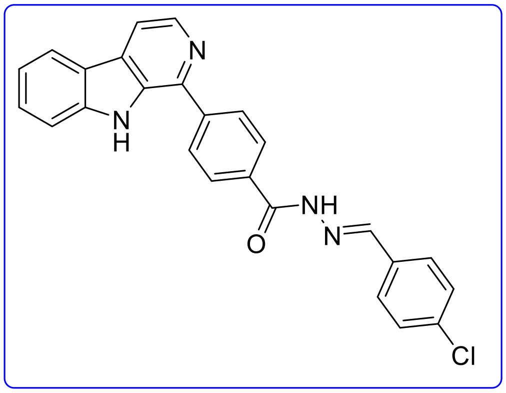

Data for **9g**: white solid, yield: 48%; ^{1}H NMR (600 MHz, DMSO-d_6) δ: 12.12 (s, 1H, H-9), 11.64 (s, 1H), 8.54 (s, 1H), 8.51 (d, J = 4.8 Hz, 1H), 8.29 (d, J = 7.8 Hz, 1H), 8.19-8.18 (m, 5H, -Ph), 7.80 (d, J = 8.4 Hz, 2H), 7.67 (d, J = 8.4 Hz, 1H), 7.58-7.55 (m, 3H), 7.30-7.27 (m, 1H); ^{13}C NMR (150 MHz, DMSO-d_6) δ: 163.3, 147.1, 142.0, 141.6, 141.5, 139.0, 135.0, 133.7, 133.3, 130.0, 129.5, 129.5, 129.5, 129.5, 129.2, 129.2, 128.9, 128.8, 128.6, 122.2, 121.2, 120.1, 115.0, 112.9; MS (ESI) m/z calcd for $C_{25}H_{17}ClN_4O$ ([M+H]$^+$), 425.1164; found, 425.1174.

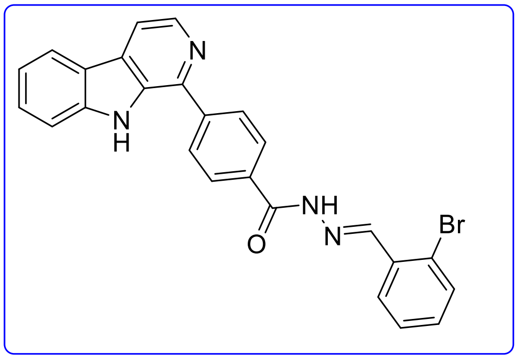

Data for **9h**: white solid, yield: 28%; ^{1}H NMR (600 MHz, DMSO-d_6) δ: 12.33 (s, 1H, H-9), 11.67 (s, 1H), 8.93 (s, 1H), 8.51 (d, J = 4.8 Hz, 1H), 8.30 (d, J = 7.8 Hz, 1H), 8.21-8.19 (m, 5H, -Ph), 8.06 (d, J = 7.8 Hz, 1H), 7.73 (d, J = 7.8 Hz, 1H), 7.67 (d, J = 8.4 Hz, 1H), 7.59-7.57 (m, 1H, -Ph), 7.53-7.50 (m, 1H, -Ph), 7.41-7.39 (m, 1H, -Ph), 7.30-7.28 (m, 1H, -Ph); ^{13}C NMR (150 MHz, DMSO-d_6) δ: 163.3, 146.6, 142.1, 141.7, 141.5, 139.0, 133.7, 133.2, 132.3, 130.0, 128.9, 128.8, 128.6, 127.8, 124.1, 122.2, 121.2, 120.1, 115.0, 112.9. MS (ESI) m/z calcd for $C_{25}H_{17}$ BrN_4O ($[M+H]^+$), 469.0659; found, 469.0659.

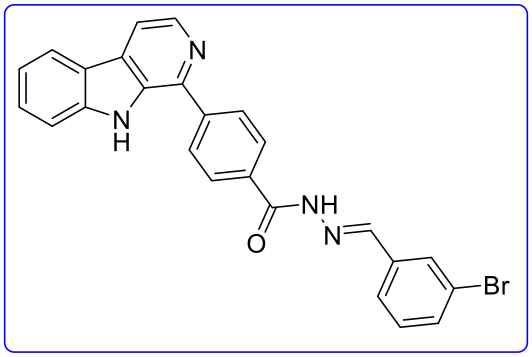

Data for **9i**: white solid, yield: 34%; ^{1}H NMR (600 MHz, DMSO-d_6) δ: 12.20 (s, 1H, H-9), 11.65 (s, 1H), 8.51 (d, J = 4.8 Hz, 2H), 8.29 (d, J = 7.8 Hz, 1H), 8.19-8.18 (m, 5H, -Ph), 7.97 (s, 1H), 7.77 (d, J = 7.2 Hz, 1H), 7.68-7.64 (m, 2H), 7.57 (t, J = 7.2 Hz, 1H), 7.45 (t, J = 7.8 Hz, 1H), 7.29 (t, J = 7.2 Hz, 1H); ^{13}C NMR (150 MHz, DMSO-d_6) δ: 163.4, 146.6, 142.1, 141.6, 141.5, 139.0, 137.3, 133.9, 133.2, 133.1, 131.6, 130.0, 129.6, 128.9, 128.8, 128.6, 126.8, 122.7, 122.2, 121.2, 120.1, 115.0, 112.9; MS (ESI) m/z calcd for $C_{25}H_{17}$ BrN_4O ($[M+H]^+$), 469.0659; found, 469.0668.

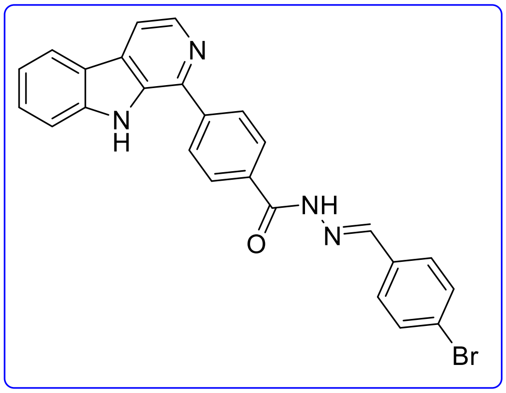

Data for **9j**: white solid, yield: 49%; ^{1}H NMR (600 MHz, DMSO-d_6) δ: 12,17 (s, 1H, H-9), 11.66 (s, 1H), 8.54-8.50 (m, 2H), 8.29 (d, J = 7.8 Hz, 1H), 8.19-8.18 (m, 5H, -Ph), 7.72-7.66 (m, 5H), 7.59-7.56 (m, 1H), 7.30-7.27 (m, 1H); ^{13}C NMR (150 MHz, DMSO-d_6) δ: 163.3, 147.2, 142.0, 141.7, 141.5, 139.0, 134.2, 133.7, 132.4, 132.4, 132.4, 132.4, 130.0, 129.5, 129.5, 128.9, 128.9, 128.8, 128.6, 123.8, 122.2, 121.2, 120.1, 115.0, 112.9; MS (ESI) m/z calcd for $C_{25}H_{17}$ BrN_4O ($[M+H]^+$),469.0659; found, 469.0670.

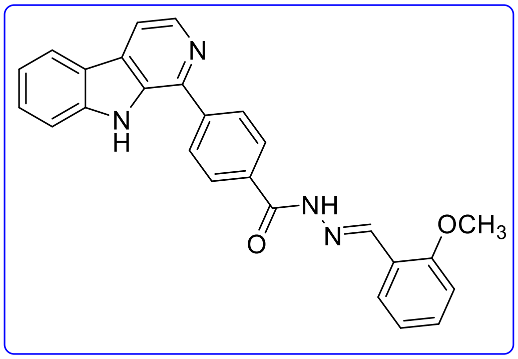

Data for **9k**: white solid, yield: 41%; ^{1}H NMR (600 MHz, DMSO-d_6) δ: 12.02 (s, 1H, H-9), 11.64 (s, 1H), 8.89 (s, 1H), 8.51 (d, J = 4.8 Hz, 1H), 8.29 (d, J = 7.8 Hz, 1H), 8.19-8.18 (m, 5H, -Ph), 7.92 (d, J = 7.2 Hz, 1H), 7.67 (d, J = 8.4 Hz, 1H), 7.59-7.56 (m, 1H), 7.46-7.43 (m, 1H), 7.30-7.27 (m, 1H), 7.13 (d, J = 8.4 Hz, 1H), 7.07-7.04 (m, 1H), 3.89 (s, 3H, -OCH_3); ^{13}C NMR (150 MHz, DMSO-d_6) δ: 163.0, 158.3, 143.9, 141.9, 141.6, 141.5, 139.0, 133.7, 133.4, 132.1, 130.0, 128.9, 128.9, 128.5,

128.5, 126.0, 122.8, 122.2, 121.3, 121.3, 120.1, 114.9, 112.9, 112.4, 56.2; MS (ESI) m/z calcd for $C_{26}H_{20}N_4O_2$ ($[M+H]^+$), 421.1659; found, 421.1666.

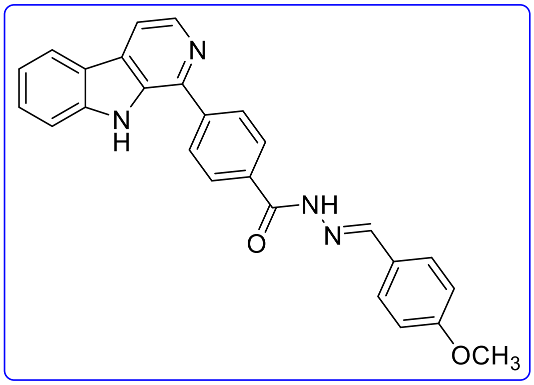

Data for **9l**: white solid, yield: 80%; ^{1}H NMR (600 MHz, DMSO-*d*$_6$) δ: 11.91 (s, 1H), 11.65 (s, 1H), 8.51-8.48 (m, 2H), 8.29 (d, J = 7.8 Hz, 1H), 8.19-8.16 (m, 5H), 7.72-7.67 (m, 3H), 7.59-7.56 (m, 1H), 7.30-7.27 (m, 1H), 7.05 (d, J = 8.4 Hz, 2H), 3.82 (s, 3H, -OCH_3); ^{13}C NMR (150 MHz, DMSO-*d*$_6$) δ: 163.0, 161.4, 148.4, 141.8, 141.6, 141.5, 139.0, 133.7, 130.0, 129.2, 129.2, 128.9, 128.9, 128.8, 128.5, 128.5, 127.4, 122.2, 121.2, 120.1, 114.9, 114.9, 114.9, 112.9, 55.8; MS (ESI) m/z calcd for $C_{26}H_{20}N_4O_2$ ($[M+H]^+$), 421.1659; found, 421.1670.

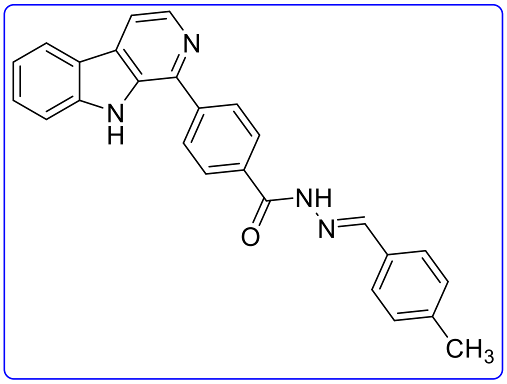

Data for **9m**: white solid, yield: 45%; ^{1}H NMR (600 MHz, DMSO-*d*$_6$) δ: 11.96 (s, 1H, H-9), 11.64,(s, 1H), 8.51-8.50 (m, 2H), 8.29 (d, J = 7.8 Hz, 1H), 8.19-8.18 (m, 5H, -Ph), 7.67-7.66 (m, 3H), 7.59-7.56 (m, 1H), 7.30-7.29 (m, 3H), 2.36 (s, 3H, -CH_3); ^{13}C NMR (150 MHz, DMSO-*d*$_6$) δ: 163.1, 148.5,

141.9, 141.6, 141.5, 140.5, 139.0, 133.7, 133.5, 132.1, 130.0, 128.9, 128.8, 128.5, 127.6, 122.2, 121.2, 120.1, 114.9, 112.9, 21.5; MS (ESI) m/z calcd for $C_{26}H_{20}N_4O$ ($[M+H]^+$), 405.1710; found, 405.1716.

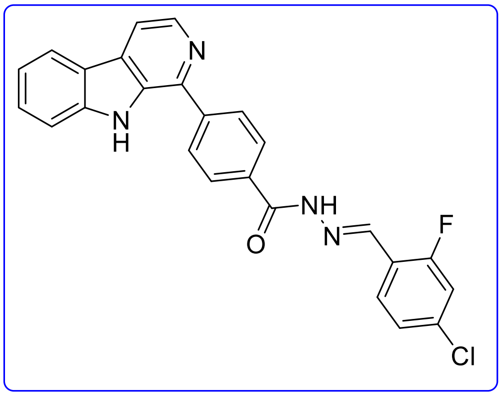

Data for **9n**: white solid, yield: 29%; ^{1}H NMR (600 MHz, DMSO-*d*$_6$) δ: 12.32 (s, 1H, H-9), 11.67 (s, 1H), 8.94 (s, 1H), 8.51 (d, *J* = 4.8 Hz, 1H), 8.30 (d, *J* = 7.8 Hz, 1H), 8.21-8.19 (m, 5H, -Ph), 8.13-8.11 (m, 1H), 7.68 (d, *J* = 7.8 Hz, 1H), 7.59-7.57 (m, 2H), 7.40-7.37 (m, 1H), 7.30-7.28 (m, 1H); ^{13}C NMR (150 MHz, DMSO-*d*$_6$) δ: 172.5, 169.2, 143.4, 142.1, 141.7, 141.4, 139.0, 134.6 (J_{CF} = 10.5 Hz), 133.7, 133.1, 130.0, 128.9, 128.8, 128.6, 122.2, 121.2, 120.1, 117.6 (J_{CF} = 25.5 Hz), 116.0 (J_{CF} = 21.0 Hz), 115.0, 112.9; MS (ESI) m/z calcd for $C_{25}H_{16}ClFN_4O$ ($[M+H]^+$), 443.1069; found, 443.1075. The coupling between F and C1 was not detected in ^{13}C NMR spectrum.

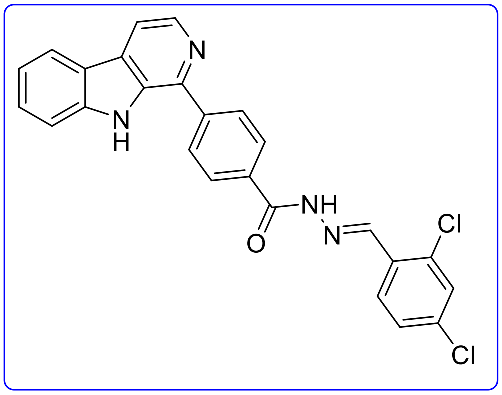

Data for **9o**: white solid, yield: 47%; ^{1}H NMR (600 MHz, DMSO-d_6) δ: 12.31 (s, 1H, H-9), 11.64 (s, 1H), 8.90 (s, 1H), 8.51 (d, J = 4.8 Hz, 1H), 8.30 (d, J = 8.4 Hz, 1H), 8.20-8.19 (m, 5H, -Ph), 8.08 (d, J = 8.4 Hz, 1H), 7.76 (s, 1H), 7.67 (d, J = 7.8 Hz, 1H), 7.59-7.57 (m, 2H), 7.30-7.28 (m, 1H); ^{13}C NMR (150 MHz, DMSO-d_6) δ: 163.2, 143.2, 142.2, 141.6, 141.4, 139.0, 135.6, 134.4, 133.7, 133.1, 131.2, 129.9, 129.0, 128.8, 128.6, 122.2, 121.2, 120.1, 115.0, 112.9; MS (ESI) m/z calcd for $C_{25}H_{16}Cl_2N_4O$ ([M+H]$^+$), 459.0774; found, 459.0779.

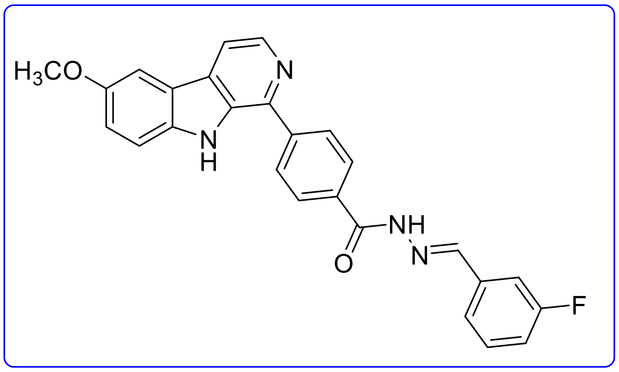

Data for **10c**: white solid, yield: 55%; ^{1}H NMR (600 MHz, DMSO-d_6) δ: 12.14 (s, 1H, H-9), 11.46 (s, 1H), 8.54 (s, 1H), 8.47 (d, J = 4.8 Hz, 1H), 8.19-8.16 (m, 5H, -Ph), 7.84 (d, J = 2.4 Hz, 1H), 7.58-7.56 (m, 4H), 7.31 (t, J = 7.2 Hz, 1H), 7.23 (dd, J = 9.0 Hz, 2.4 Hz, 1H), 3.89 (s, 3H, -OCH$_3$); ^{13}C NMR (150 MHz, DMSO-d_6) δ: 162.7, 162.2, 153.0, 146.0, 141.1, 140.4, 137.3, 135.4, 133.1, 132.2, 130.4, 128.8, 127.8, 127.5, 122.9, 120.5, 117.9, 114.0, 112.7, 112.4 (J_{CF} = 22.5 Hz), 102.8, 55.0; MS (ESI) m/z calcd for $C_{26}H_{19}FN_4O_2$ ([M+H]$^+$), 439.1565; found, 439.1572. The coupling between F and C1 was not detected in ^{13}C NMR spectrum.

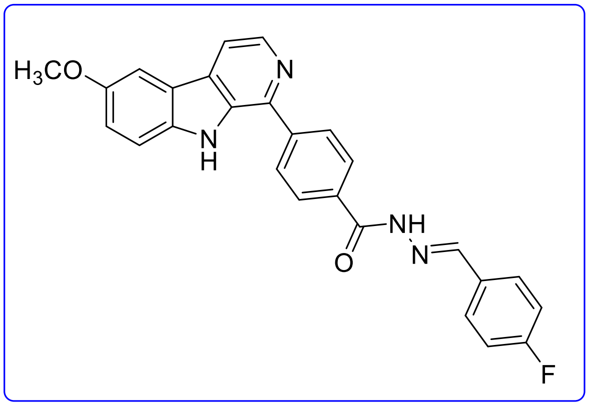

Data for **10d**: white solid, yield: 13%; ^{1}H NMR (600 MHz, DMSO-d_6) δ: 12.04 (s, 1H, H-9), 11.45 (s, 1H), 8.53 (s, 1H), 8.46 (d, J = 5.4 Hz, 1H), 8.18-8.16 (m, 5H, -Ph), 7.84-7.83 (m, 3H), 7.57 (d, J = 9.0 Hz, 1H), 7.33 (t, J = 8.4 Hz, 2H), 7.23-7.21 (m, 1H), 3.89 (s, 3H, -OCH$_3$); ^{13}C NMR (150 MHz, DMSO-d_6) δ 162.5, 153.4, 146.6, 141.3, 140.8, 137.7, 135.8, 133.5, 132.7, 129.2, 129.1 (J_{CF} = 9.0 Hz), 128.1, 127.8, 120.9, 118.2, 115.7 (J_{CF} = 22.5 Hz), 114.3, 113.0, 103.2, 55.4; MS (ESI) m/z calcd for $C_{26}H_{19}FN_4O_2$ ([M+H]$^+$), 439.1656; found, 439.1578. The coupling between F and C1 was not detected in ^{13}C NMR spectrum.

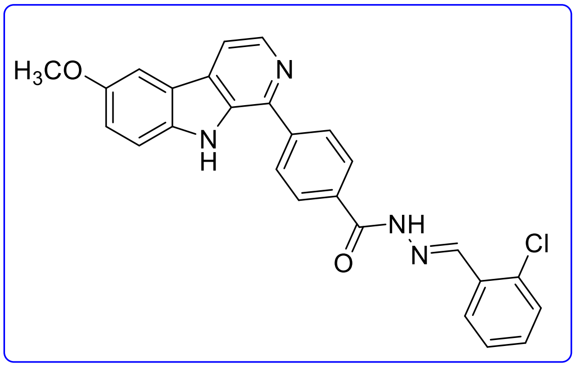

Data for **10e**: white solid, yield: 69%; ^{1}H NMR (600 MHz, DMSO-d_6) δ: 12.27 (s, 1H, H-9), 11.55 (s, 1H), 8.96 (s, 1H), 8.48 (d, J = 5.4 Hz, 1H), 8.21 (s, 5H, -Ph), 8.09-8.07 (m, 1H), 7.87 (d, J = 2.4 Hz, 1H), 7.59-7.57 (m, 2H), 7.49-7.47 (m, 2H), 7.25-7.23 (m, 1H), 3.89 (s, 3H, -OCH$_3$); ^{13}C NMR (150 MHz, DMSO-d_6) δ: 163.2, 154.2, 144.3, 136.8, 134.2, 133.7, 133.1, 132.1, 131.3, 130.5, 129.9, 129.0,

128.6, 128.2, 127.7, 127.4, 121.5, 119.3, 115.2, 113.8, 103.9, 56.1; MS (ESI) m/z calcd for $C_{26}H_{19}ClN_4O_2$ ($[M+H]^+$), 455.1269; found, 455.1283.

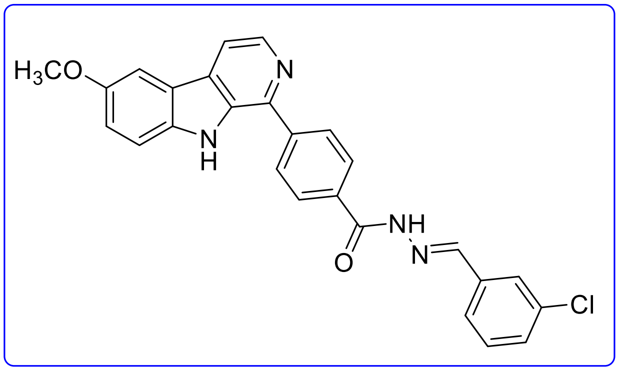

Data for **10f**: white solid, yield: 53%; ^{1}H NMR (600 MHz, DMSO-d_6) δ: 12.17 (s, 1H, H-9), 11.45 (s, 1H), 8.51 (s, 1H), 8.46 (d, *J* = 5.4 Hz, 1H), 8.19-8.16 (m, 5H, -Ph), 7.84-7.8 (m, 2H), 7.73 (s, 1H), 7.57-7.53 (m, 3H), 7.22 (dd, *J* = 8.4 Hz, 2.4 Hz, 1H), 3.88 (s, 3H, -OCH_3); MS (ESI) m/z calcd for $C_{26}H_{19}ClN_4O_2$ ($[M+H]^+$), 455.1269; found, 455.1279.

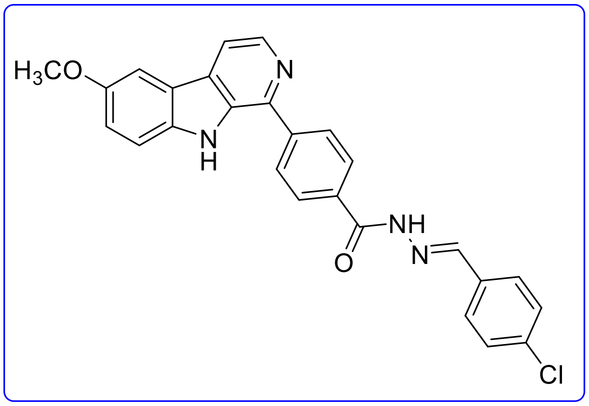

Data for **10g**: white solid, yield: 14%; ^{1}H NMR (600 MHz, DMSO-d_6) δ: 12.09 (s, 1H), 11.46 (s, 1H), 8.53 (s, 1H), 8.45 (d, *J* = 4.8 Hz, 1H), 8.19-8.17 (m, 4H, -Ph), 7.94 (d, *J* = 8.4 Hz, 1H), 7.84 (d, *J* = 2.4 Hz, 1H), 7.80 (d, *J* = 8.4 Hz, 2H), 7.69 (d, *J* = 8.4 Hz, 1H), 7.58-7.55 (m, 2H), 7.23 (dd, *J* = 8.4 Hz, 2.4 Hz, 1H), 3.89 (s, 3H, -OCH_3); ^{13}C NMR (150 MHz, DMSO-d_6) δ: 163.2, 154.1, 147.1, 142.0, 141.5, 139.8, 138.4, 136.5, 134.2, 133.3, 131.6, 129.9,129.9, 129.5, 129.5, 129.2, 129.2, 128.8,

128.8, 128.5, 121.6, 119.0, 115.0, 113.8, 103.9, 56.1; MS (ESI) m/z calcd for $C_{26}H_{19}ClN_4O_2$ ($[M+H]^+$), 455.1269; found, 455.1281.

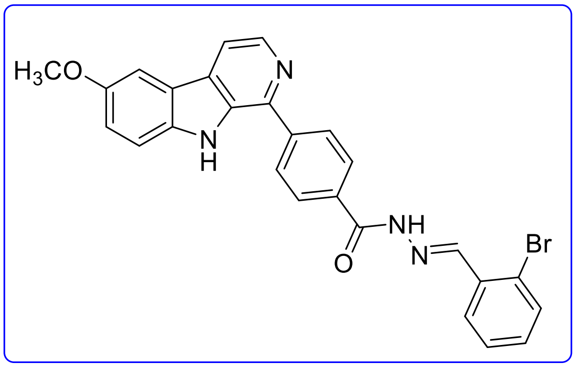

Data for **10h**: white solid, yield: 26%; ^{1}H NMR (600 MHz, DMSO-d_6) δ: 12.38 (s, 1H, H-9), 12.07 (s, 1H), 8.93 (s,1H), 8.55 (d, J = 5.4 Hz, 2H), 8.27-8.20 (m, 4H, -Ph), 8.06 (d, J = 7.2 Hz, 1H), 7.98 (s, 1H), 7.73 (d, J = 7.8 Hz, 1H), 7.64 (d, J = 8.4 Hz, 1H), 7.53-7.50 (m, 1H), 7.42-7.39 (m, 1H), 7.34 (d, J = 7.8 Hz, 1H), 3.91 (s, 3H, -OCH_3); ^{13}C NMR (150 MHz, DMSO-d_6) δ: 161.9, 153.6, 145.8, 137.1, 133.1, 132.7, 132.6, 132.55, 131.9, 131.3, 130.0, 128.6, 127.7, 127.6, 126.7, 123.1, 120.1, 115.0, 113.1, 103.0, 55.1; MS (ESI) m/z calcd for $C_{26}H_{19}BrN_4O_2$ ($[M+H]^+$), 499.0764; found, 499.0777.

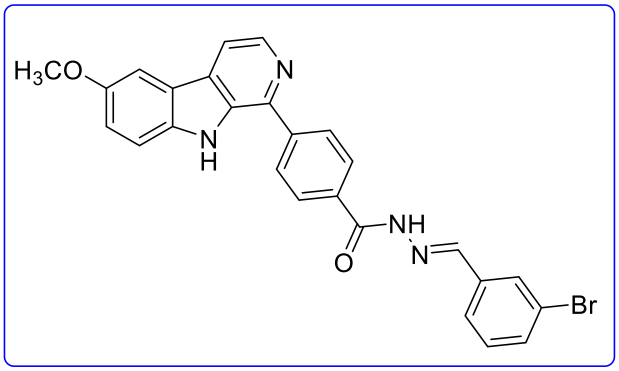

Data for **10i**: white solid, yield: 30%; ^{1}H NMR (600 MHz, DMSO-d_6) δ: 12.16 (s, 1H, H-9), 11.46 (s, 1H), 8.50-8.46 (m, 2H), 8.19-8.16 (m, 4H, -Ph), 8.04 (s, 1H), 7.97-7.93 (m, 2H), 7.84-7.83 (m, 1H), 7.57 (d, J = 8.4 Hz, 1H), 7.49-7.47 (m, 2H), 7.22 (dd, J = 9.0 Hz, 2.4 Hz, 1H), 3.88 (s, 3H, -OCH_3);

^{13}C NMR (150 MHz, DMSO-d_6) δ: 165.4, 153.0, 145.5, 141.4, 140.4, 137.3, 135.4, 135.0, 133.1, 132.5, 131.1, 130.3, 128.6, 127.8, 127.7, 127.5, 121.6, 121.1, 120.5, 117.9, 114.0, 112.7, 102.8, 55.0; MS (ESI) m/z calcd for $C_{26}H_{19}BrN_4O_2$ ($[M+H]^+$), 499.0764; found, 499.0773.

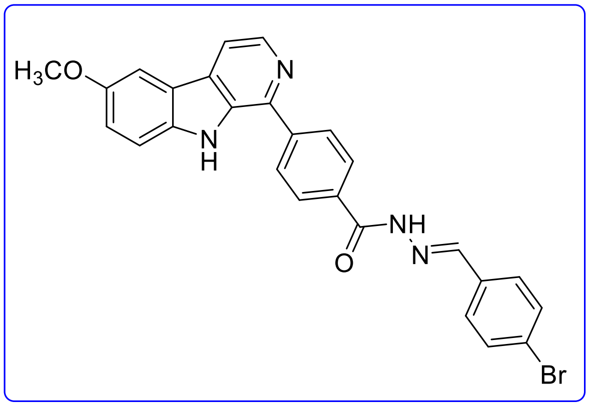

Data for **10j**: white solid, yield: 65%; ^1H NMR (600 MHz, DMSO-d_6) δ: 12.31 (s, 2H), 8.64 (s, 1H), 8.59 (d, J = 6.0 Hz, 2H), 8.30 (d, J = 7.8 Hz, 2H), 8.20 (d, J = 8.4 Hz, 2H), 8.05 (s, 1H), 7.72-7.69 (m, 5H, -Ph), 7.40 (d, J = 7.2 Hz, 1H), 3.92 (s, 3H, -OCH_3); ^{13}C NMR (150 MHz, DMSO-d_6) δ: 162.8, 154.9, 147.6, 139.0, 138.1, 135.3, 134.1, 133.6, 132.4, 130.1, 129.5, 128.9, 124.0, 122.3, 120.9, 116.6, 114.4, 104.2, 56.2; MS (ESI) m/z calcd for $C_{26}H_{19}BrN_4O_2$ ($[M+H]^+$), 499.0764; found, 499.0780.

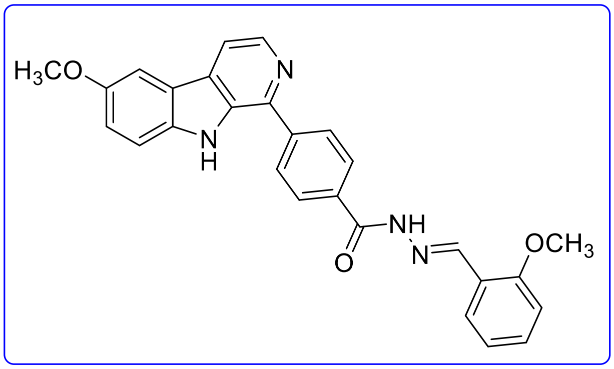

Data for **10k**: white solid, yield: 57%; ^1H NMR (600 MHz, DMSO-d_6) δ: 12.02 (s, 1H, H-9), 11.46 (s, 1H), 8.90 (s, 1H), 8.47 (d, J = 4.8 Hz, 1H), 8.17-8.16 (m, 5H, -Ph), 7.92 (d, J = 7.2 Hz, 1H), 7.84 (d,

J = 2.4 Hz, 1H), 7.57 (d, J = 9.0 Hz, 1H), 7.45 (t, J = 7.2 Hz, 1H), 7.22 (dd, J = 9.0 Hz, 2.4 Hz, 1H), 7.14 (d, J = 8.4 Hz, 1H), 7.06 (t, J = 7.2 Hz, 1H), 3.90 (s, 3H, -OCH_3), 3.88 (s, 3H, -OCH_3); ^{13}C NMR (150 MHz, DMSO-d_6) δ: 161.9, 157.2, 153.0, 142.8, 140.9, 140.5, 137.3, 135.4, 133.1, 132.3, 131.0, 128.8, 127.7, 127.4, 124.9, 121.8, 120.5, 120.2, 117.8, 113.9, 112.7, 111.3, 102.8, 55.1, 55.0; MS (ESI) m/z calcd for $C_{27}H_{22}N_4O_3$ ([M+H]$^+$), 451.1765; found, 451.1773.

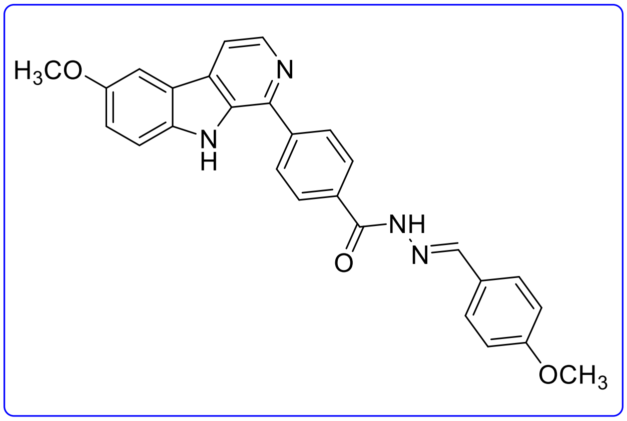

Data for **10l**: white solid, yield: 80%; ^1H NMR (600 MHz, DMSO-d_6) δ: 11.88 (s, 1H, H-9), 11.45 (s, 1H), 8.48-8.46 (m, 2H), 8.18-8.16 (m, 5H, -Ph), 7.84 (d, J = 2.4 Hz, 1H), 7.72 (d, J = 8.4 Hz, 2H), 7.57 (d, J = 9.0 Hz, 1H), 7.22 (dd, J = 9.0 Hz, 2.4 Hz, 1H), 7.05 (d, J = 9.0 Hz, 2H), 3.89 (s, 3H, -OCH_3), 3.83 (s, 3H, -OCH_3); ^{13}C NMR (150 MHz, DMSO-d_6) δ: 161.9, 160.3, 153.0, 147.3, 140.8, 140.5, 17.3, 135.4, 133.1, 132.5, 128.8, 128.1, 127.7, 127.4, 126.3, 120.5, 117.8, 113.8, 112.7, 102.8, 55.0, 54.7; MS (ESI) m/z calcd for $C_{27}H_{22}N_4O_2$ ([M+H]$^+$), 451.1765; found, 451.1777.

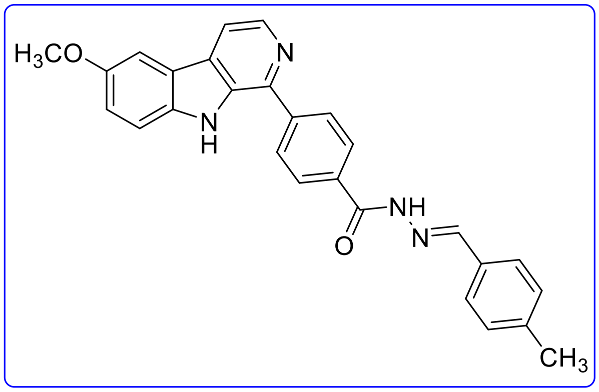

Data for **10m**: white solid, yield: 48%; ^{1}H NMR (600 MHz, DMSO-*d*$_6$) δ: 11.95 (s, 1H, H-9), 11.45 (s, 1H), 8.50 (s, 1H), 8.47 (d, *J* = 5.4 Hz, 1H), 8.18-8.16 (m, 5H, -PH), 7.84 (d, *J* = 3.0 Hz, 1H), 7.67 (d, *J* = 7.8 Hz, 2H), 7.57 (d, *J* = 8.4 Hz, 1H), 7.30 (d, *J* = 7.8 Hz, 2H), 7.22 (dd, *J* = 8.4 Hz, 2.4 Hz, 1H), 3.89 (s, 3H, -OCH$_3$), 2.37 (s, 3H, -CH$_3$); ^{13}C NMR (150 MHz, DMSO-*d*$_6$) δ: 162.0, 153.0, 147.4, 140.9, 140.4, 139.4, 137.3, 135.4, 133.1, 132.4, 127.7, 127.4, 126.5, 120.5, 117.8, 113.9, 112.7, 102.8, 55.0, 20.5; MS (ESI) m/z calcd for $C_{27}H_{22}N_4O_2$ ([M+H]$^+$), 435.1816; found, 435.1821.

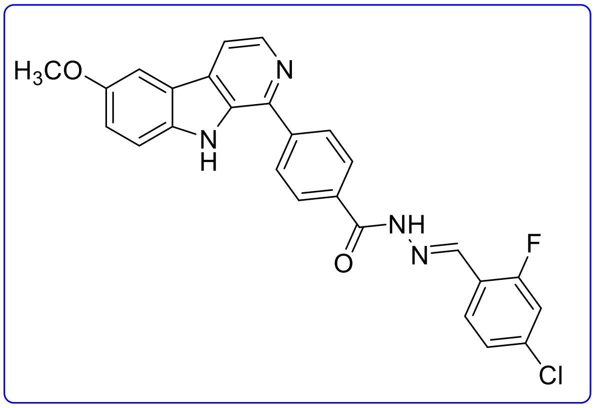

Data for **10n**: white solid, yield: 30%; ^{1}H NMR (600 MHz, DMSO-*d*$_6$) δ: 12.30 (s, 1H, H-9), 11.48 (S, 1H), 8.91 (S, 1H), 8.46 (d, *J* = 5.4 Hz, 1H), 8.20-8.16 (m, 5H, -Ph), 8.11 (t, *J* = 7.2 Hz, 1H), 7.84 (d, *J* = 2.4 Hz, 1H), 7.57 (d, *J* = 9.0 Hz, 2H), 7.37 (t, *J* = 7.2 Hz, 1H), 7.22 (dd, *J* = 8.4 Hz, 2.4 Hz, 1H), 3.88 (s, 3H, -OCH$_3$); ^{13}C NMR (150 MHz, DMSO-*d*$_6$) δ: 162.3, 154.1, 143.4, 142.2, 141.5, 138.4, 136.5, 134.2, 129.9, 129.1 (J_{CF} = 9.0 Hz), 129.0, 128.9, 128.6, 121.6, 118.9, 117.6 (J_{CF} = 25.5

Hz), 116.0 (J_{CF} = 22.5 Hz), 115.0, 113.8, 103.9, 56.1; MS (ESI) m/z calcd for $C_{26}H_{18}ClFN_4O_2$ ($[M+H]^+$), 473.1175; found, 473.1184. The coupling between F and C1 was not detected in ^{13}C NMR spectrum.

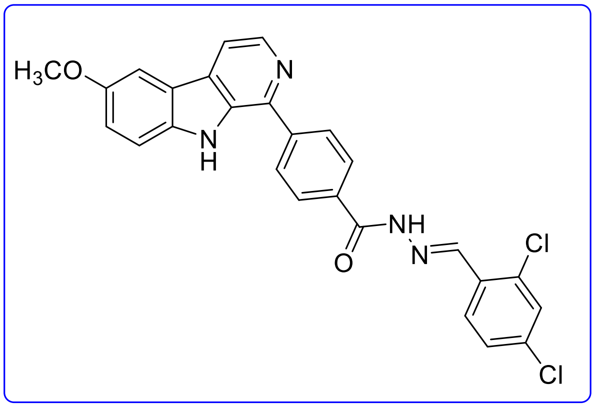

Data for **10o**: white solid, yield: 72%; ^1H NMR (600 MHz, DMSO-d_6) δ: 12.41 (s, 2H), 8.92 (s, 1H), 8.59 (s, 1H), 8.29-8.19 (m, 5H, -Ph), 8.08-8.04 (m, 2H), 7.77 (s, 1H), 7.66 (d, J = 9.0 Hz, 1H), 7.58 (d, J = 7.8 Hz, 1H), 7.39 (d, J = 7.2 Hz, 1H), 3.92 (s, 3H, -OCH_3); MS (ESI) m/z calcd for $C_{26}H_{18}Cl_2N_4O_2$ ($[M+H]^+$), 489.0880; found, 489.0896.

2. Copies of spectra of compounds 9a-o and 10a-o.

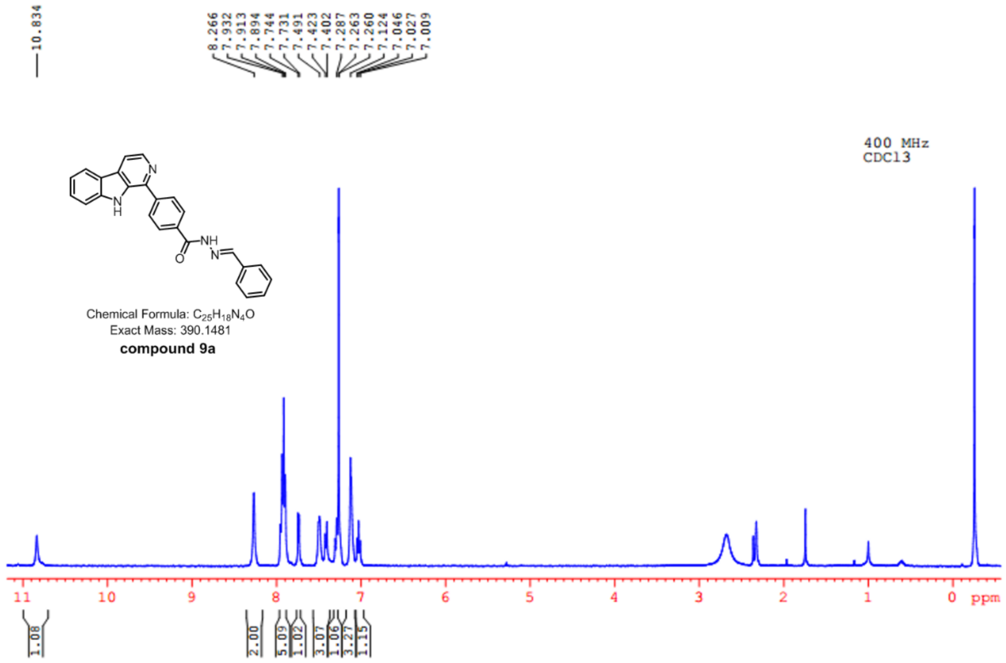

Figure S1 ^{1}H NMR spectrum of **9a**

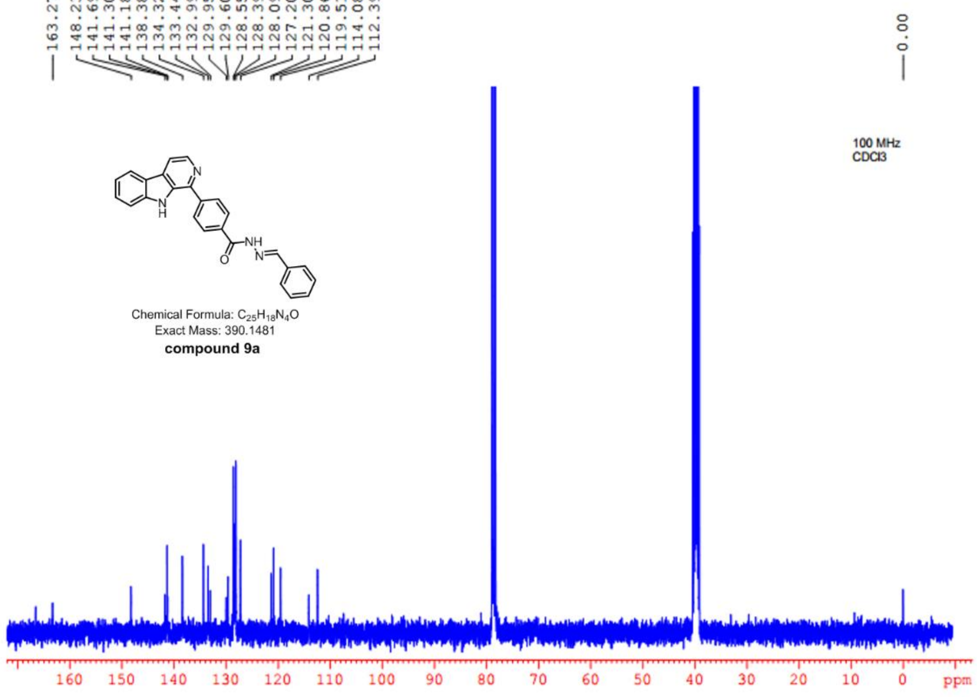

Figure S2 ^{13}C NMR spectrum of **9a**

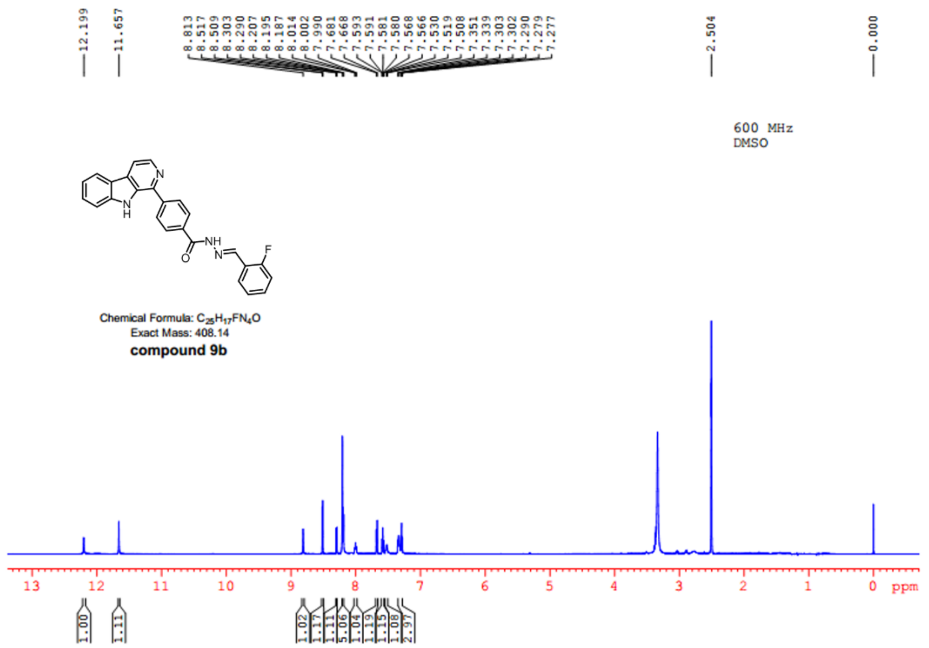

Figure S3 ^{1}H NMR spectrum of **9b**

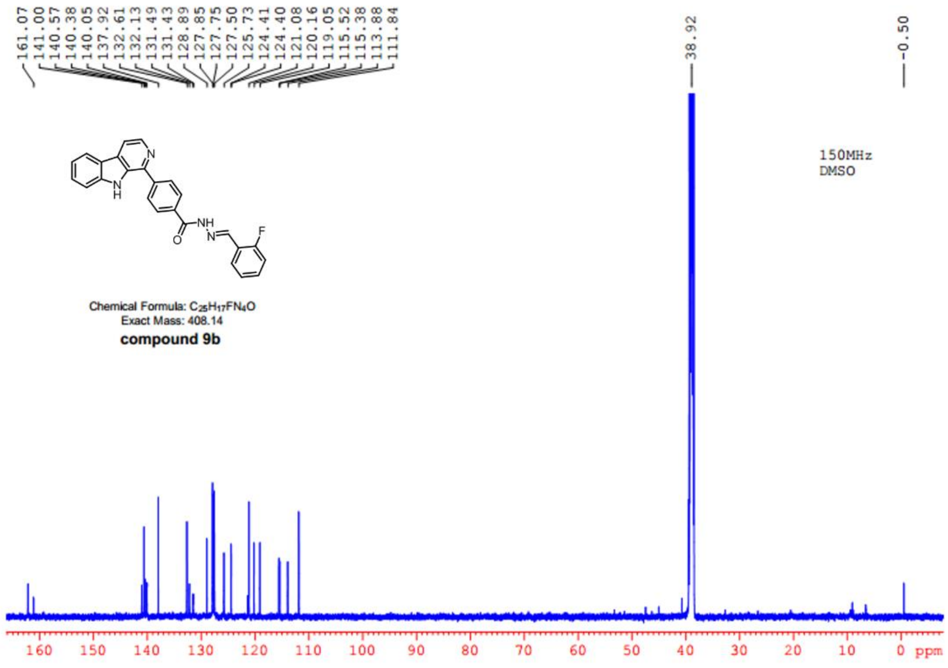

Figure S4 ^{13}C NMR spectrum of **9b**

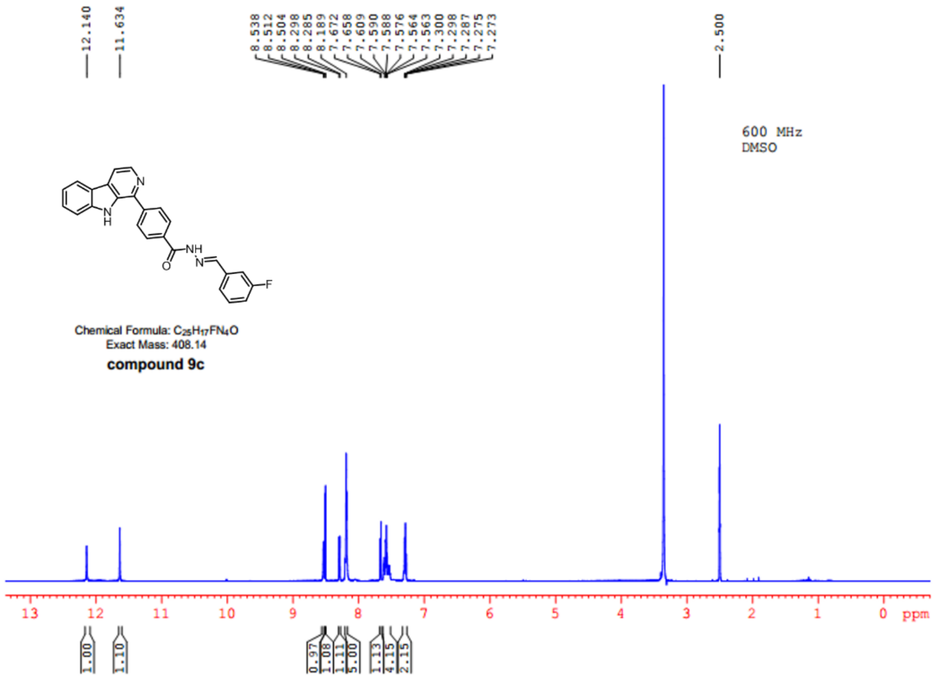

Figure S5 ^{1}H NMR spectrum of **9c**

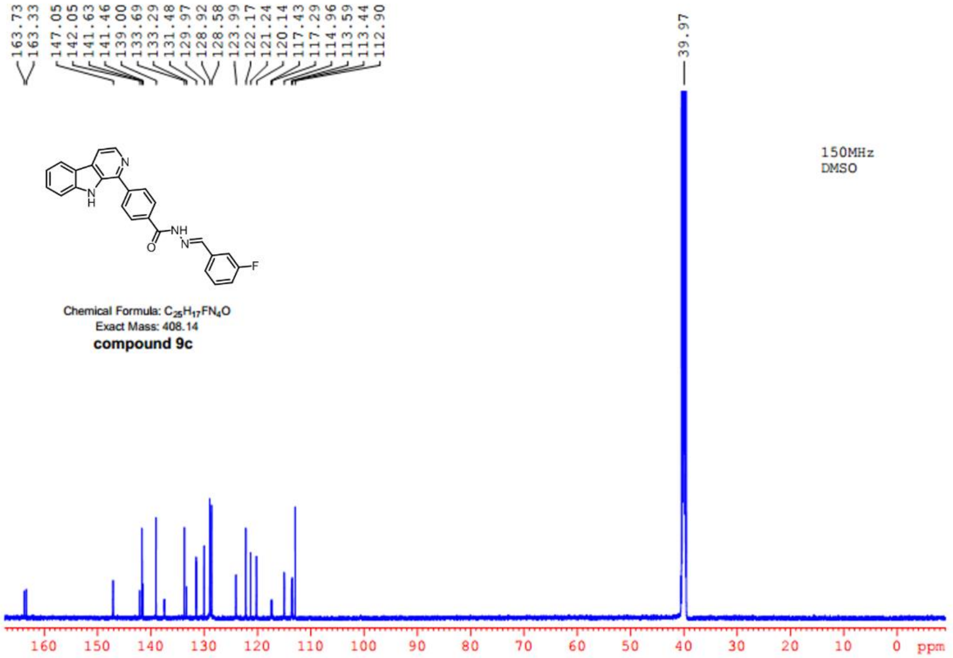

Figure S6 ^{13}C NMR spectrum of **9c**

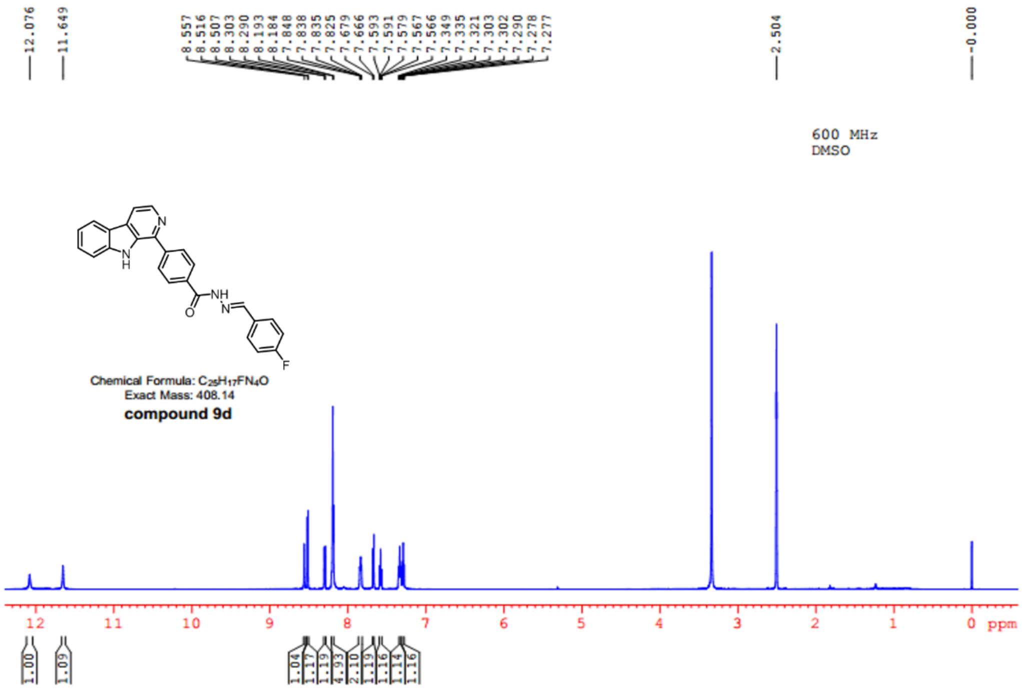

Figure S7 ^{1}H NMR spectrum of **9d**

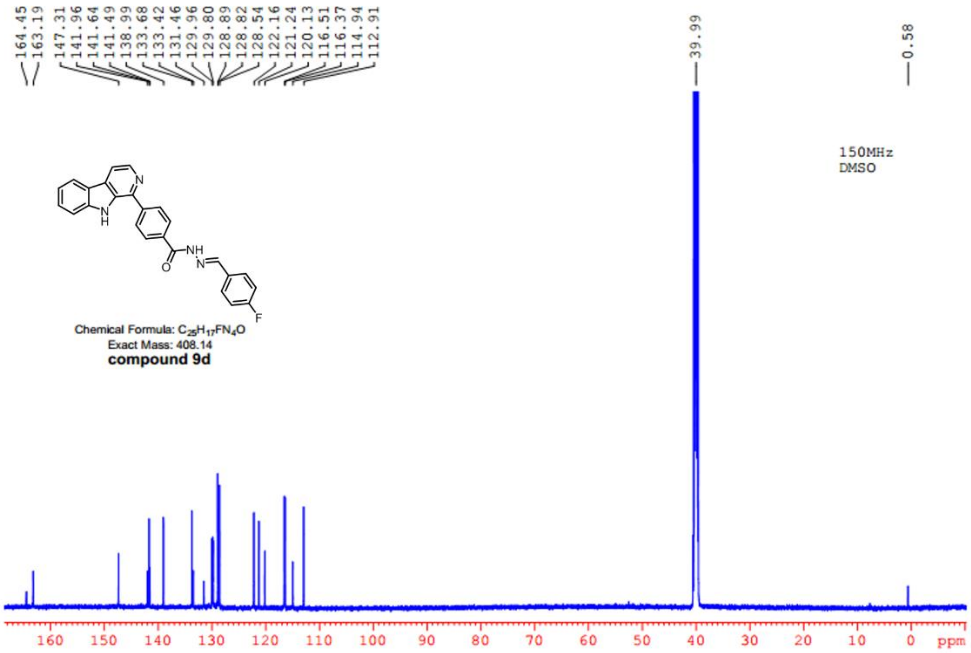

Figure S8 ^{13}C NMR spectrum of **9d**

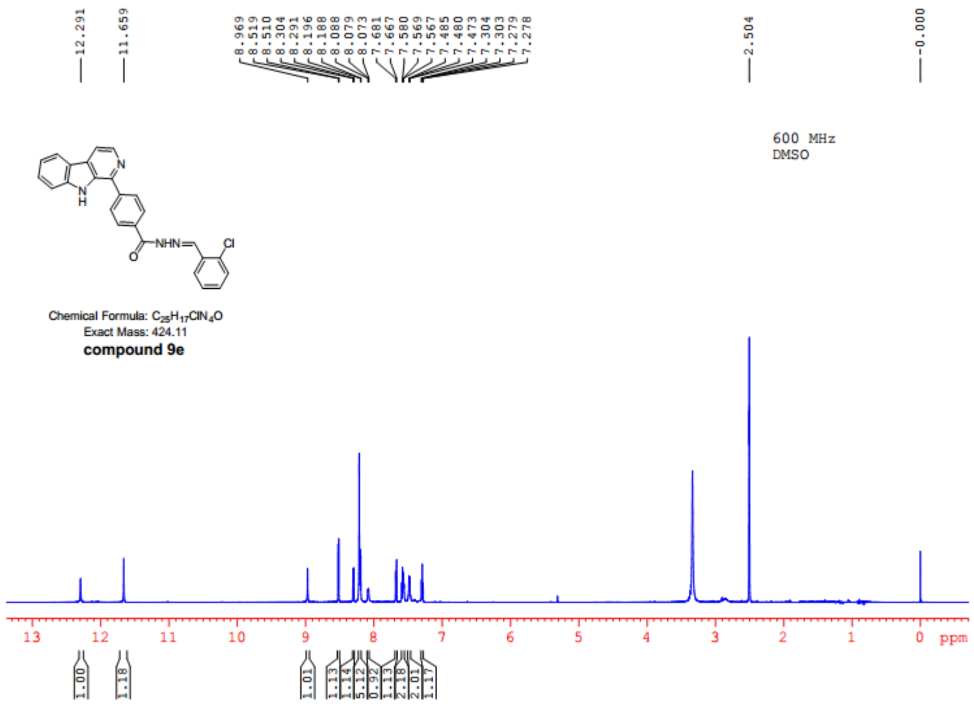

Figure S9 ^{1}H NMR spectrum of **9e**

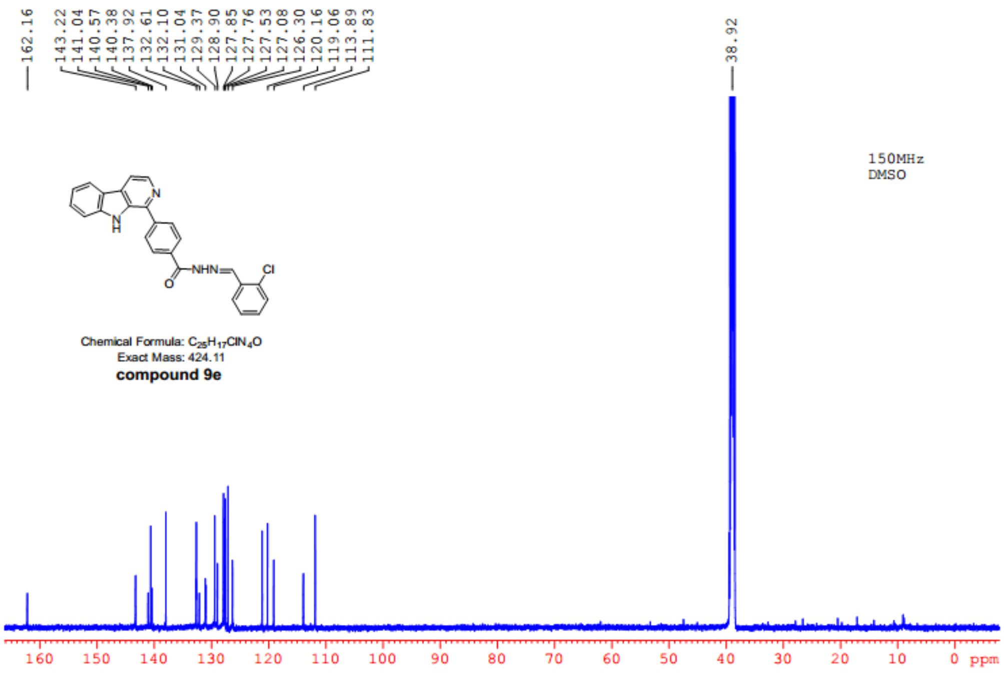

Figure S10 ^{13}C NMR spectrum of **9e**

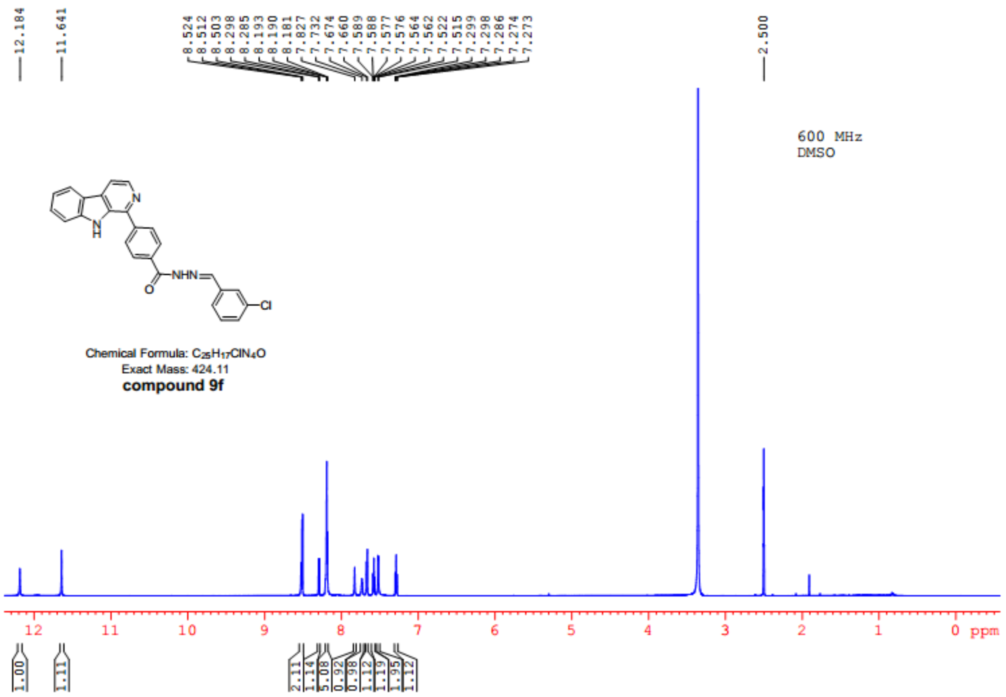

Figure S11 ^{1}H NMR spectrum of **9f**

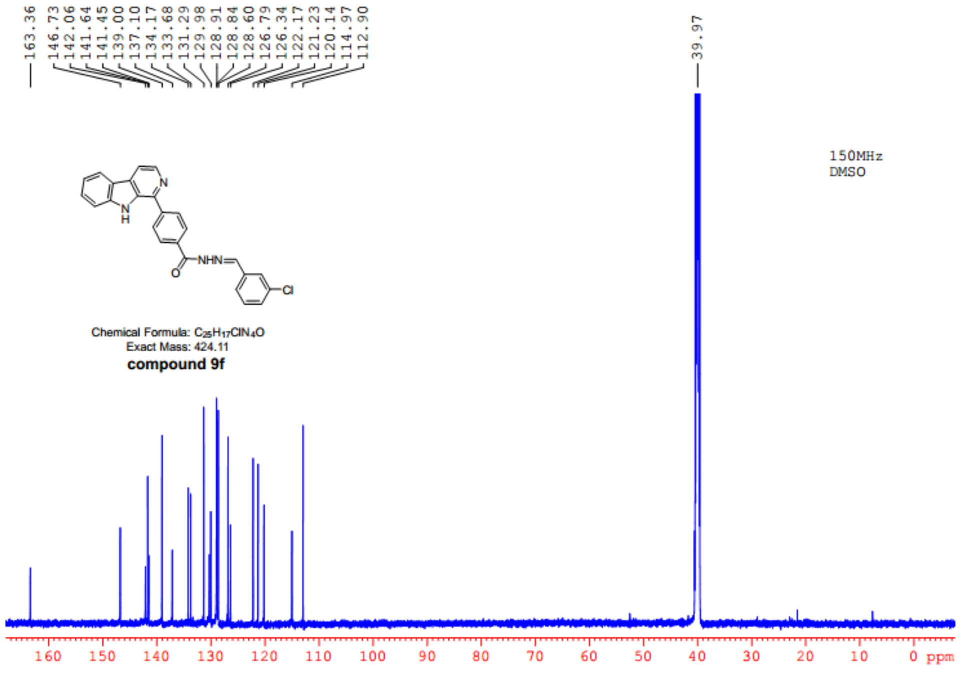

Figure S12 ^{13}C NMR spectrum of **9f**

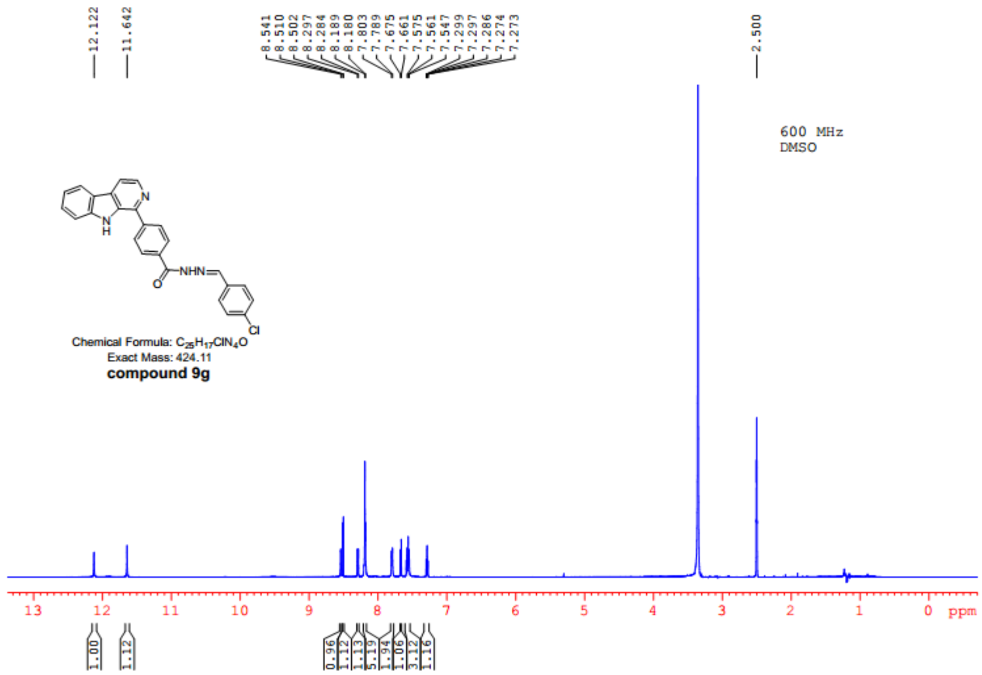

Figure S13 ^{1}H NMR spectrum of **9g**

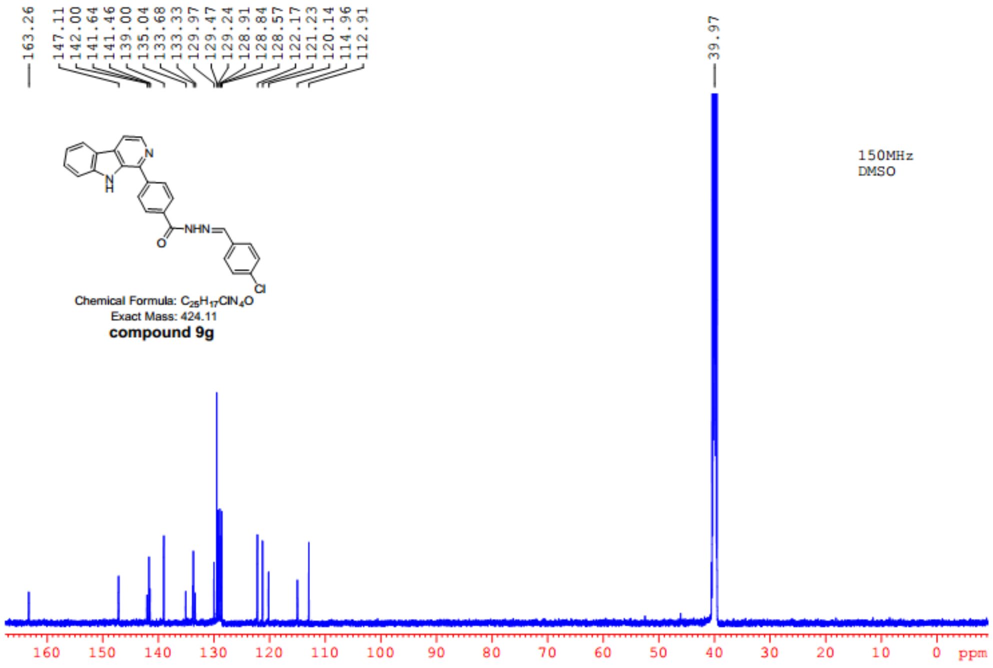

Figure S14 ^{13}C NMR spectrum of **9g**

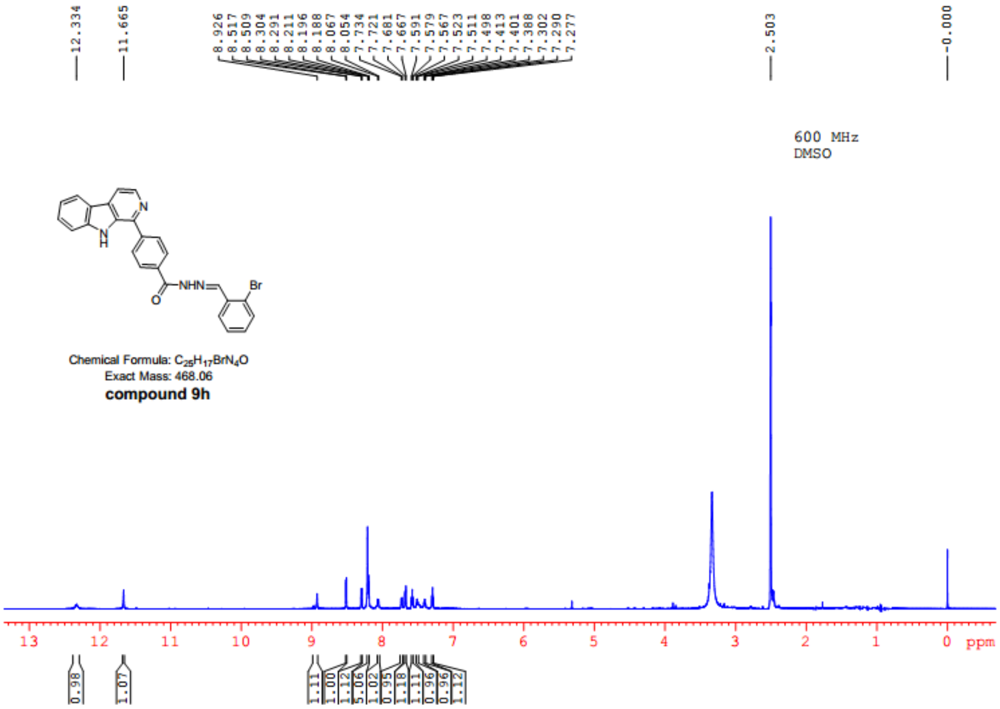

Figure S15 ^{1}H NMR spectrum of **9h**

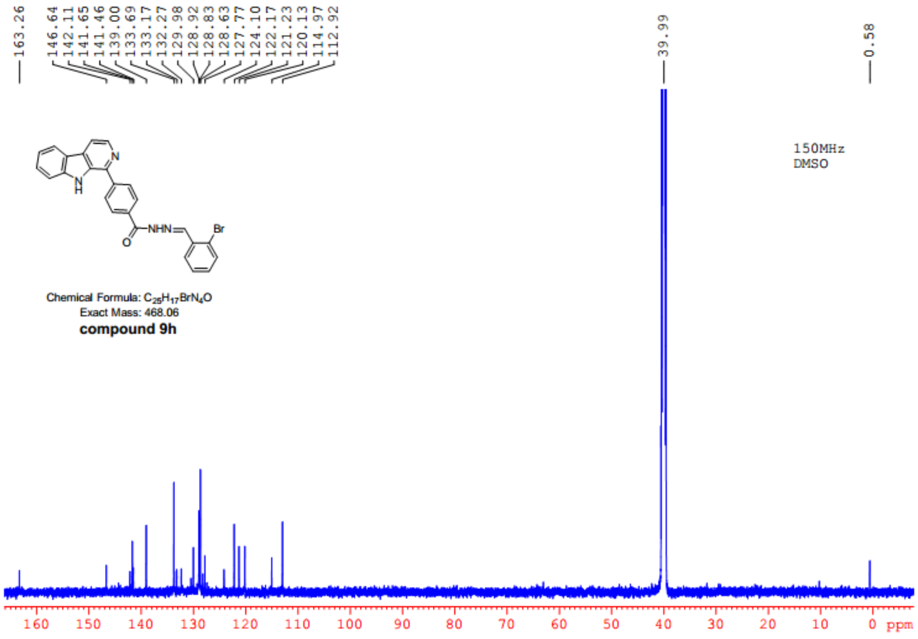

Figure S16 ^{13}C NMR spectrum of **9h**

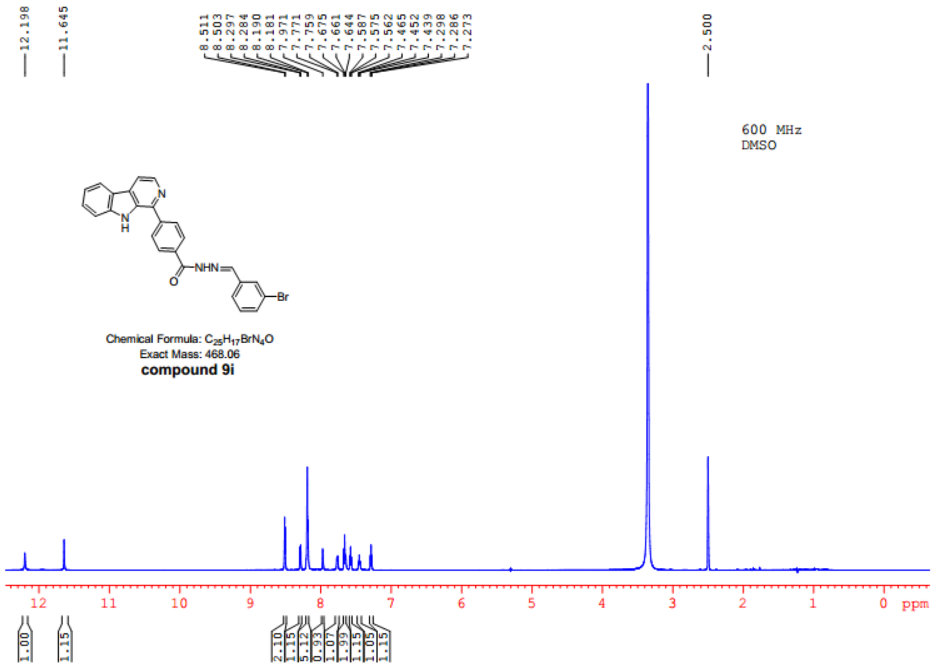

Figure S17 ^{1}H NMR spectrum of **9i**

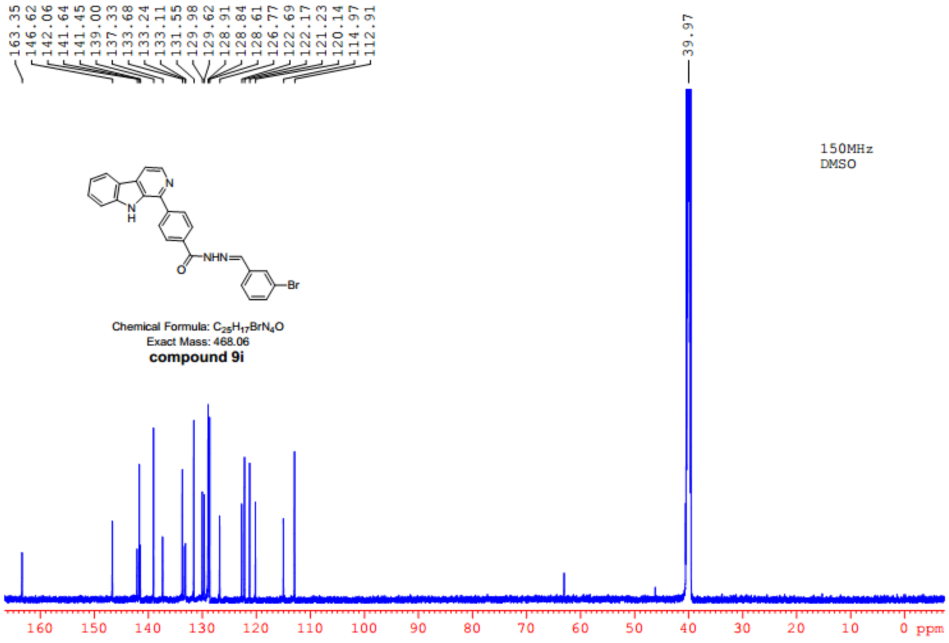

Figure S18 ^{13}C NMR spectrum of **9i**

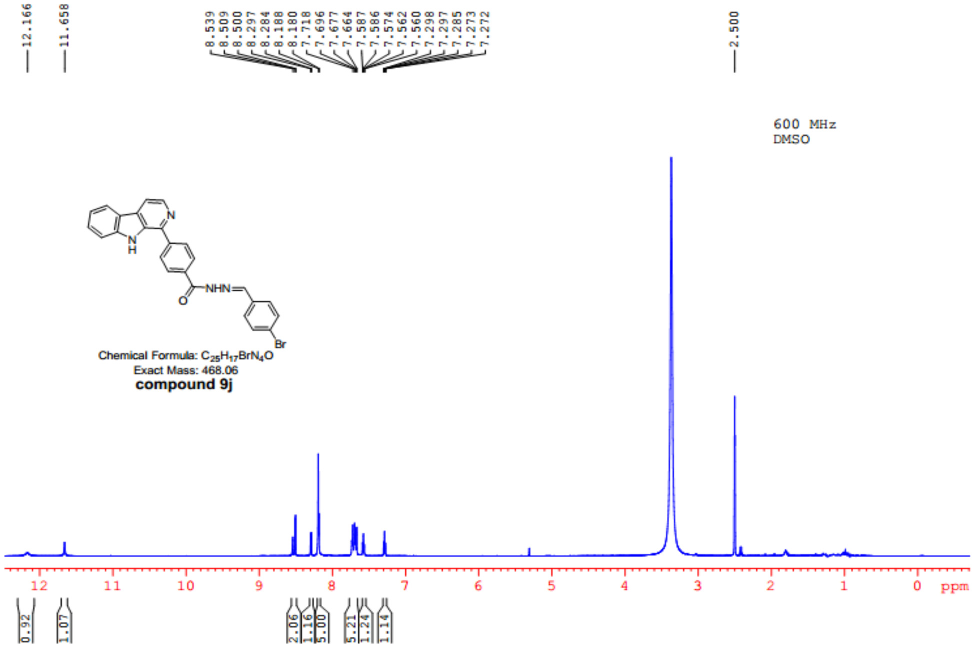

Figure S19 ^{1}H NMR spectrum of **9j**

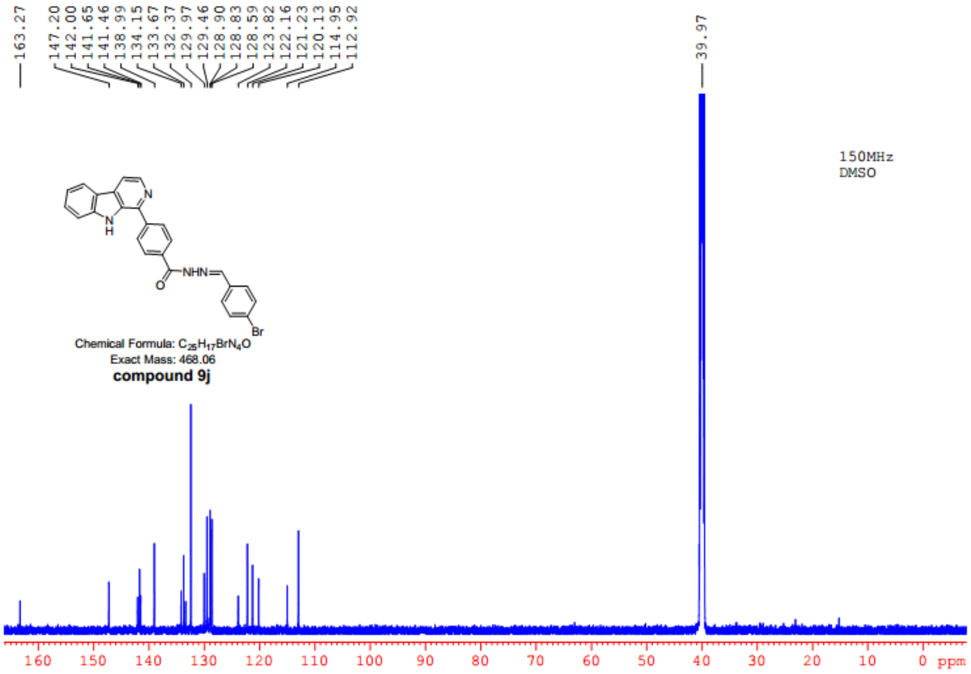

Figure S20 ^{13}C NMR spectrum of **9j**

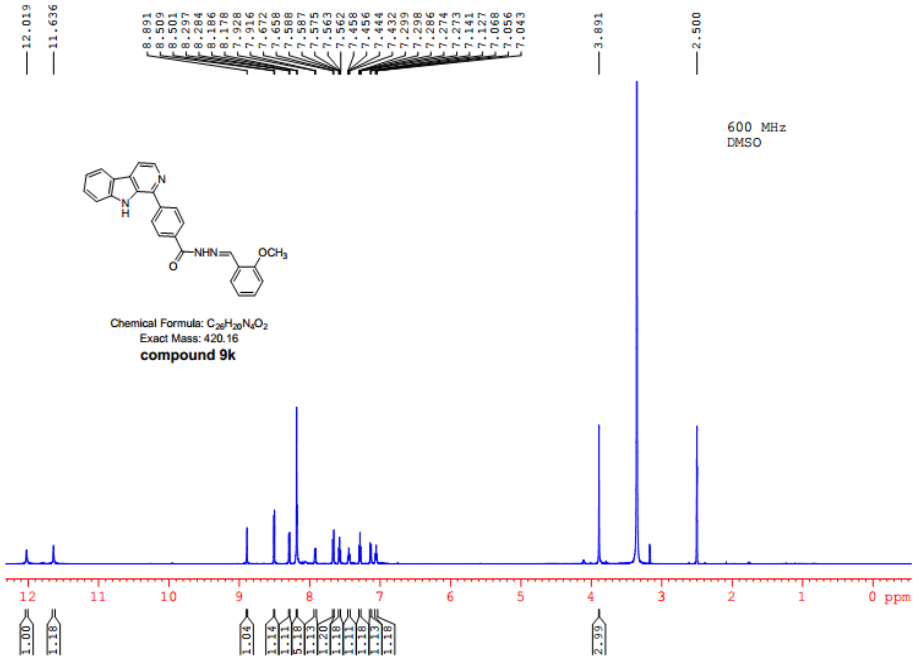

Figure S21 ^{1}H NMR spectrum of **9k**

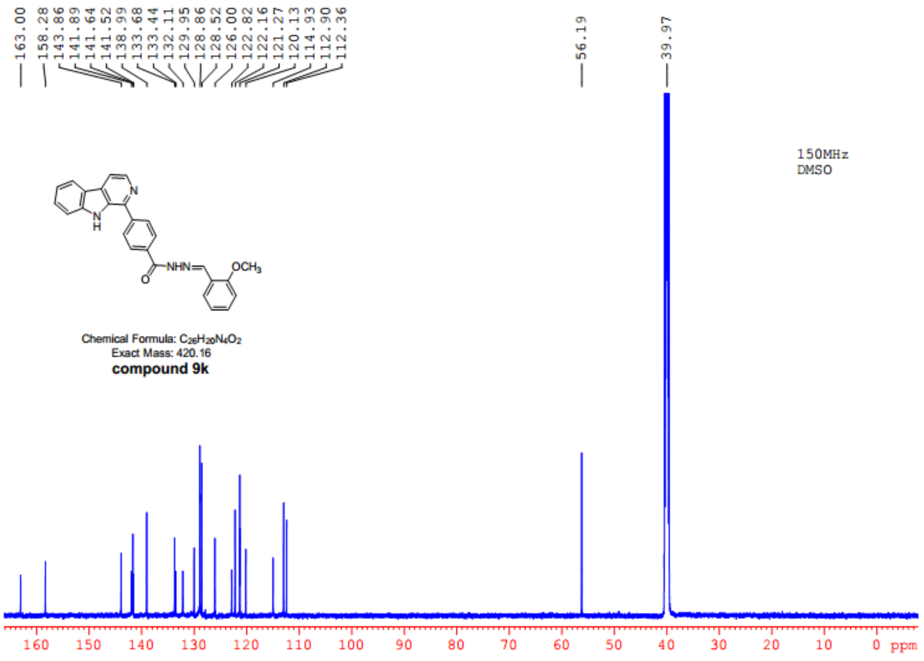

Figure S22 ^{13}C NMR spectrum of **9k**

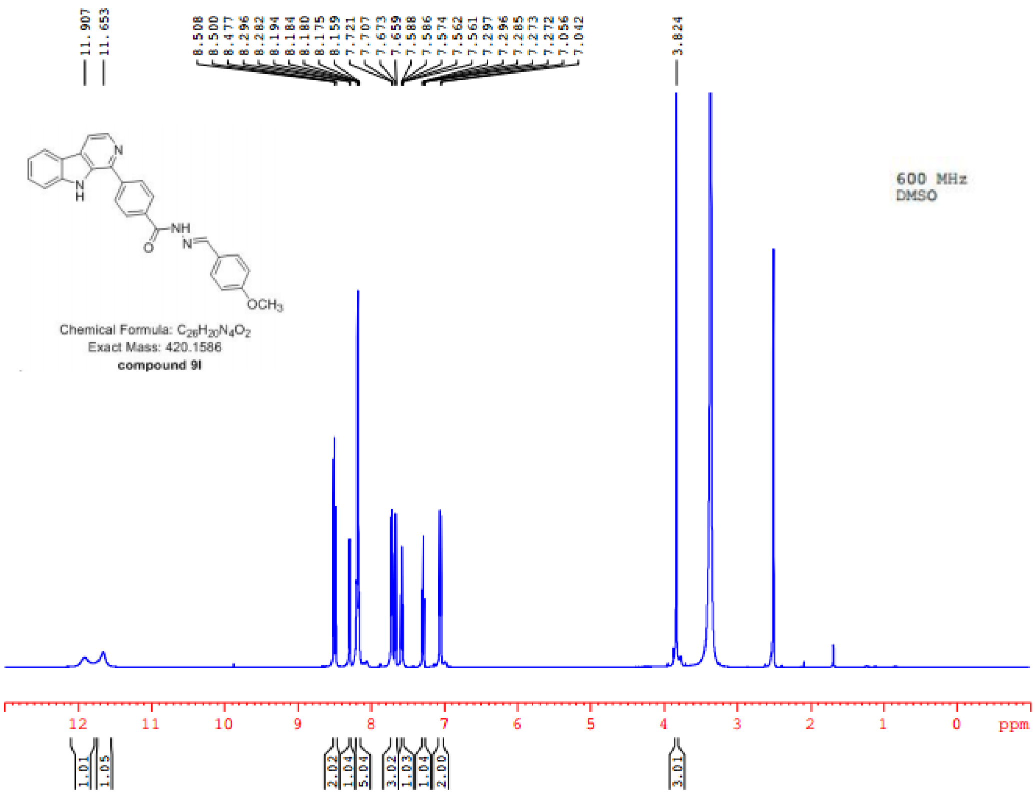

Figure S23 ^{1}H NMR spectrum of **9l**

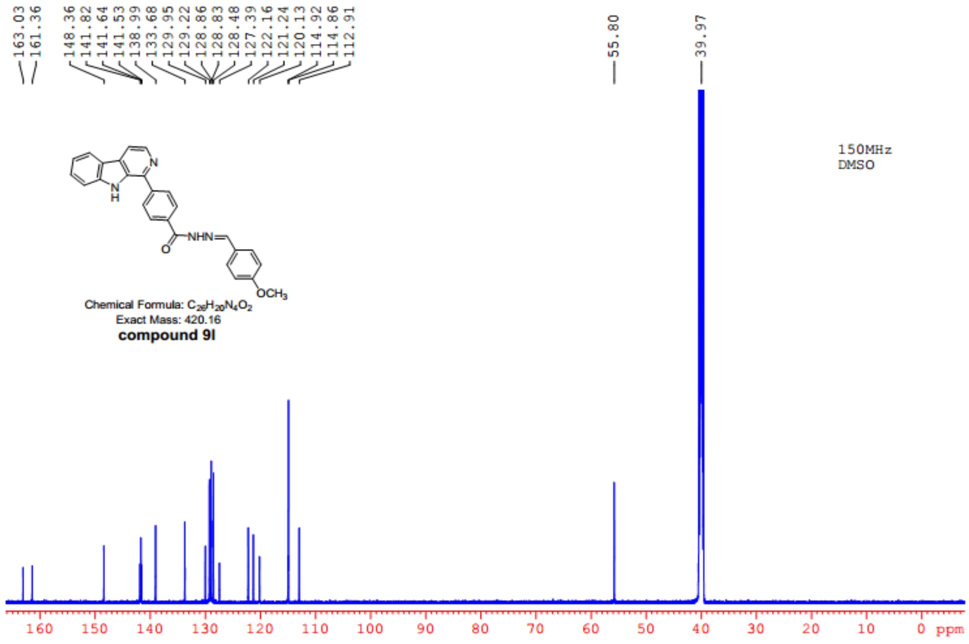

Figure S24 ^{13}C NMR spectrum of **9l**

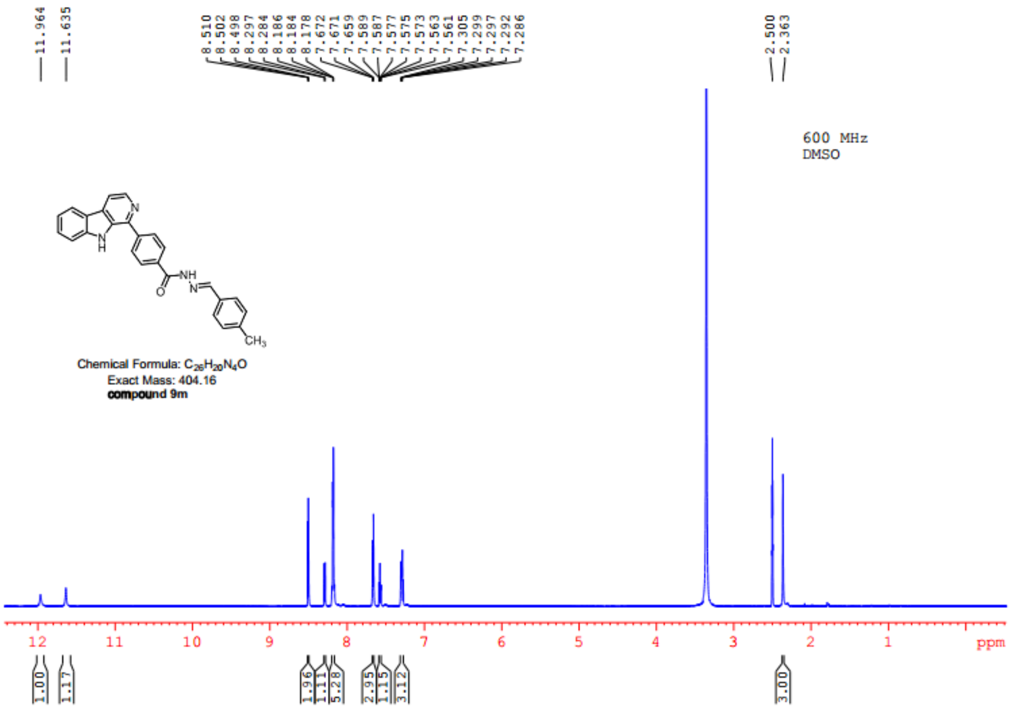

Figure S25 ^{1}H NMR spectrum of **9m**

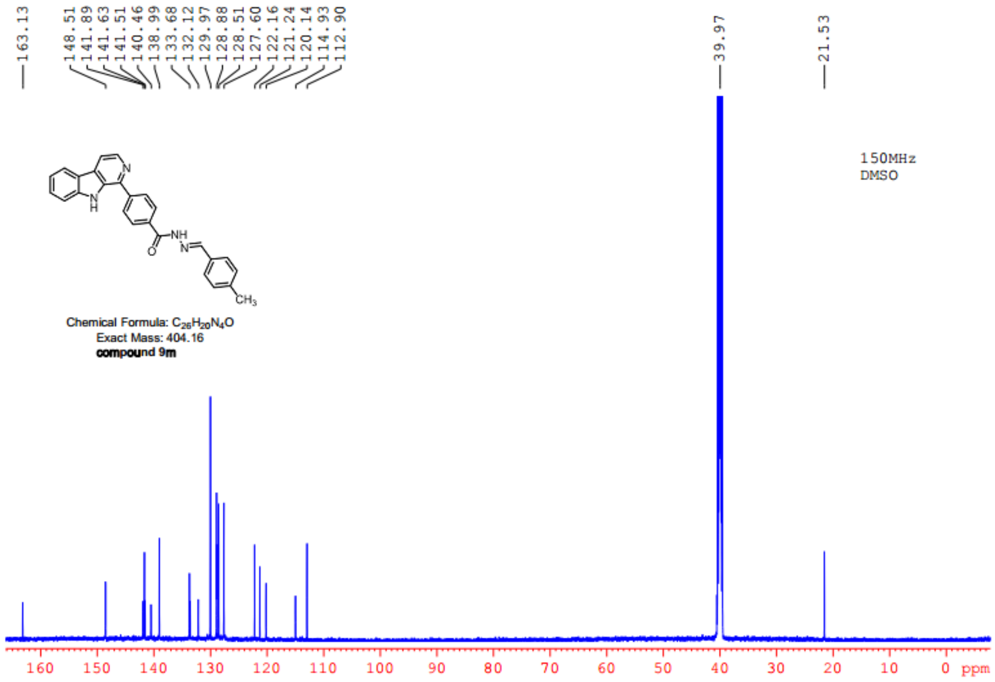

Figure S26 ^{13}C NMR spectrum of **9m**

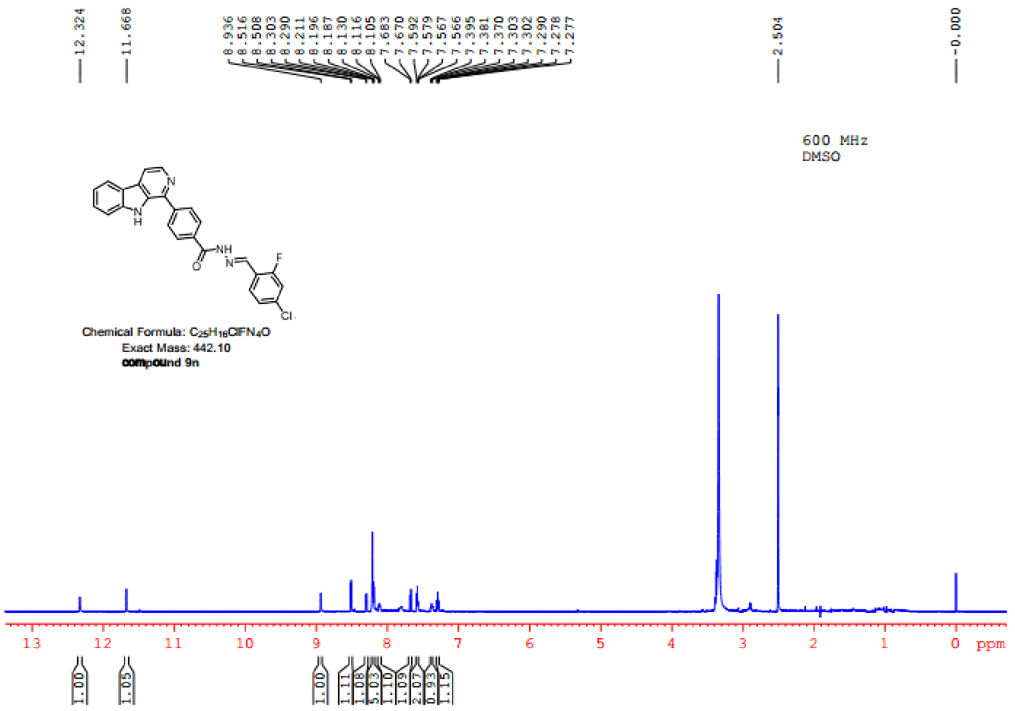

Figure S27 ¹H NMR spectrum of **9n**

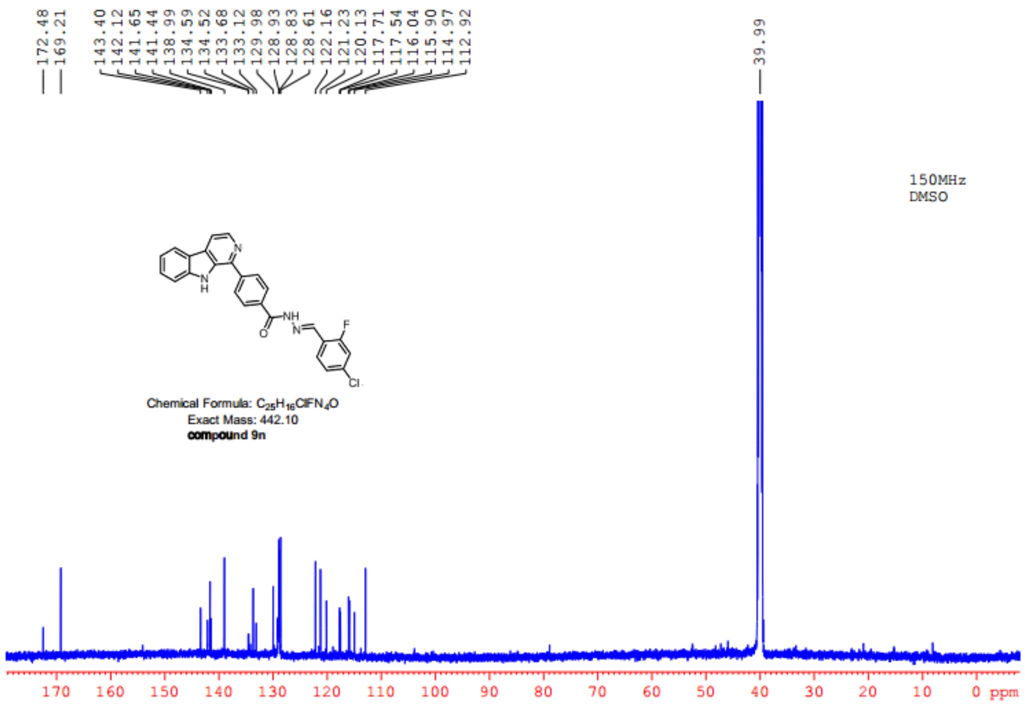

Figure S28 ¹³C NMR spectrum of **9n**

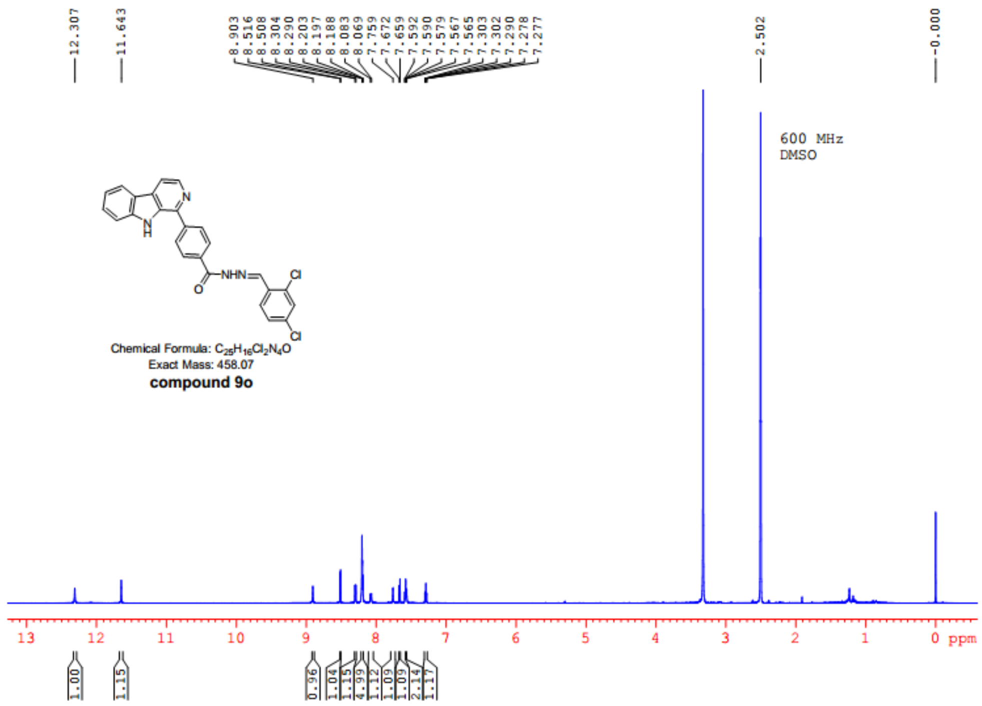

Figure S29 ^{1}H NMR spectrum of **9o**

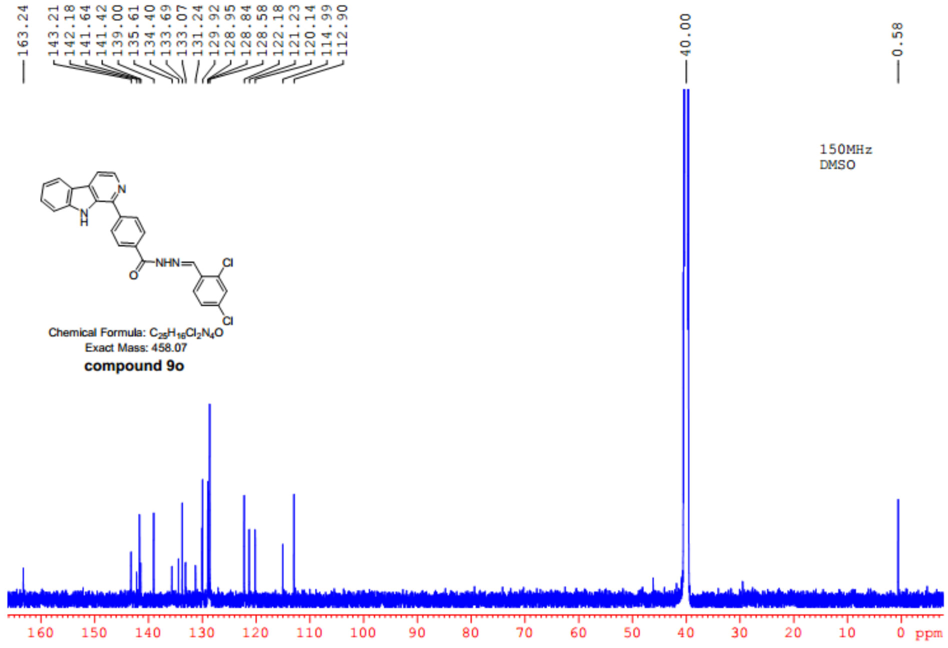

Figure S30 ^{13}C NMR spectrum of **9o**

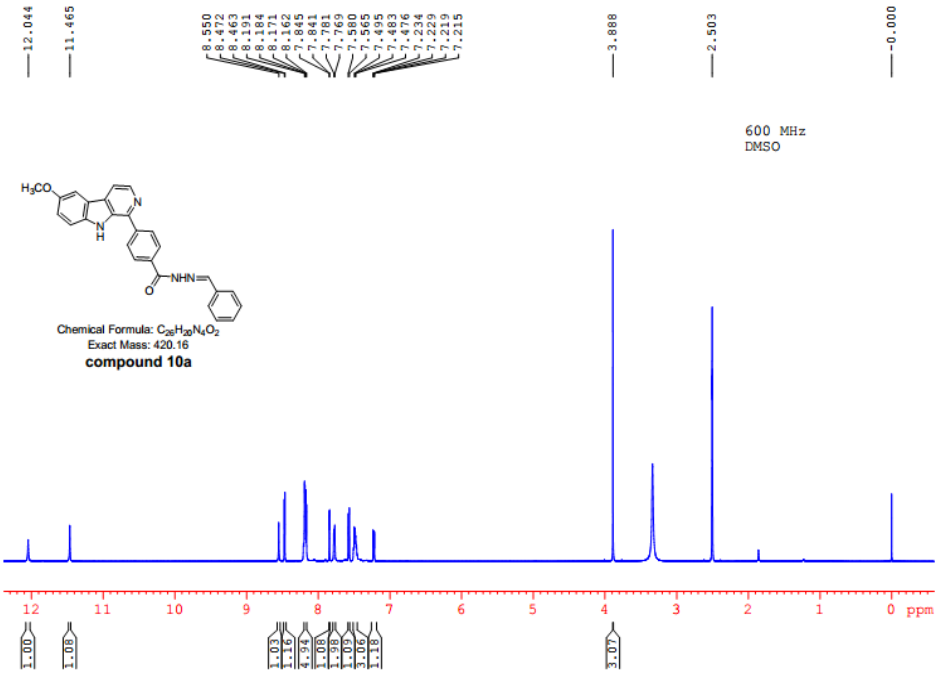

Figure S31 ^{1}H NMR spectrum of **10a**

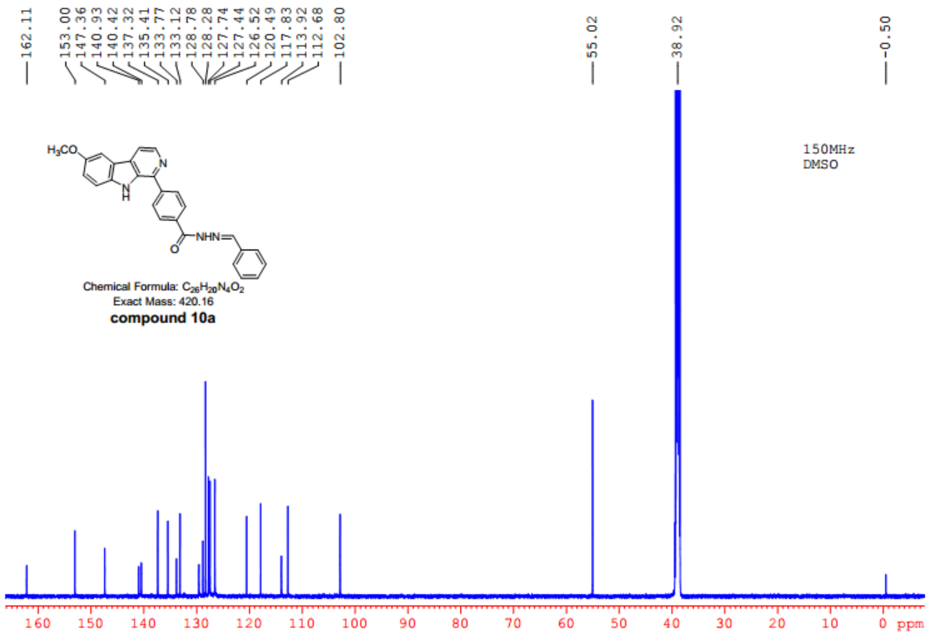

Figure S32 ^{13}C NMR spectrum of **10a**

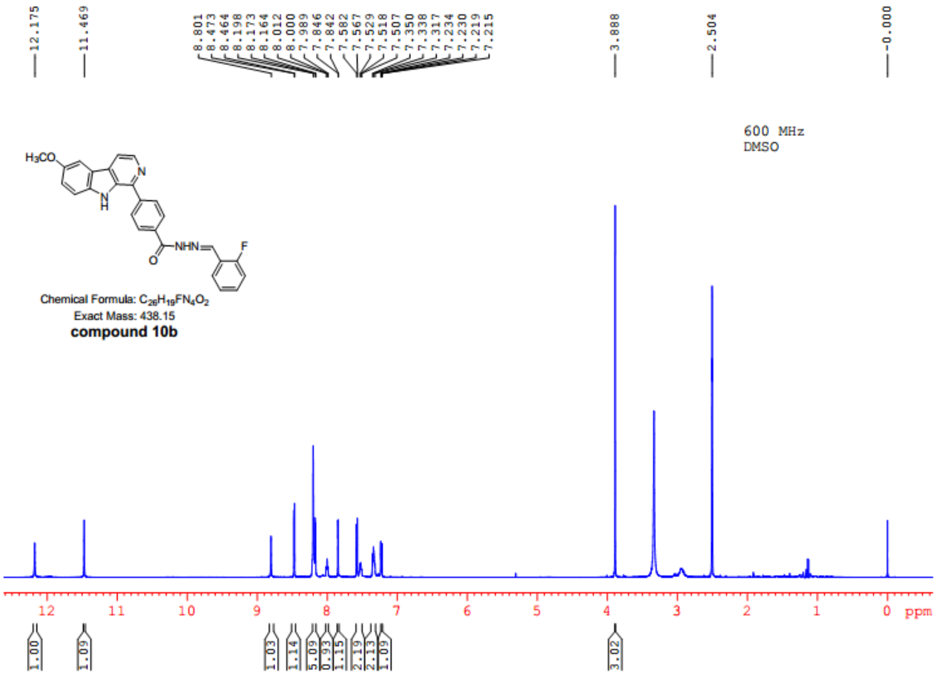

Figure S33 ^{1}H NMR spectrum of **10b**

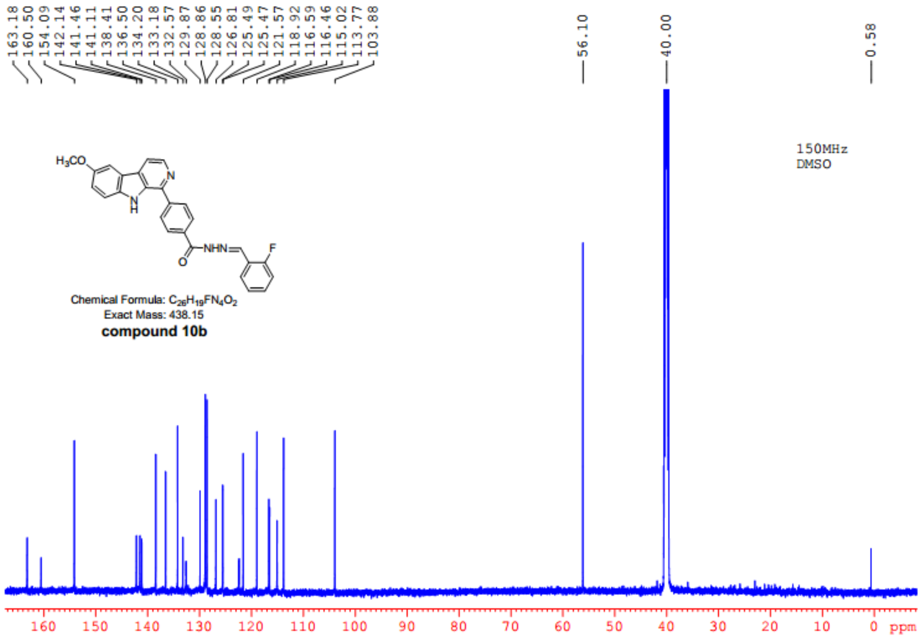

Figure S34 ^{13}C NMR spectrum of **10b**

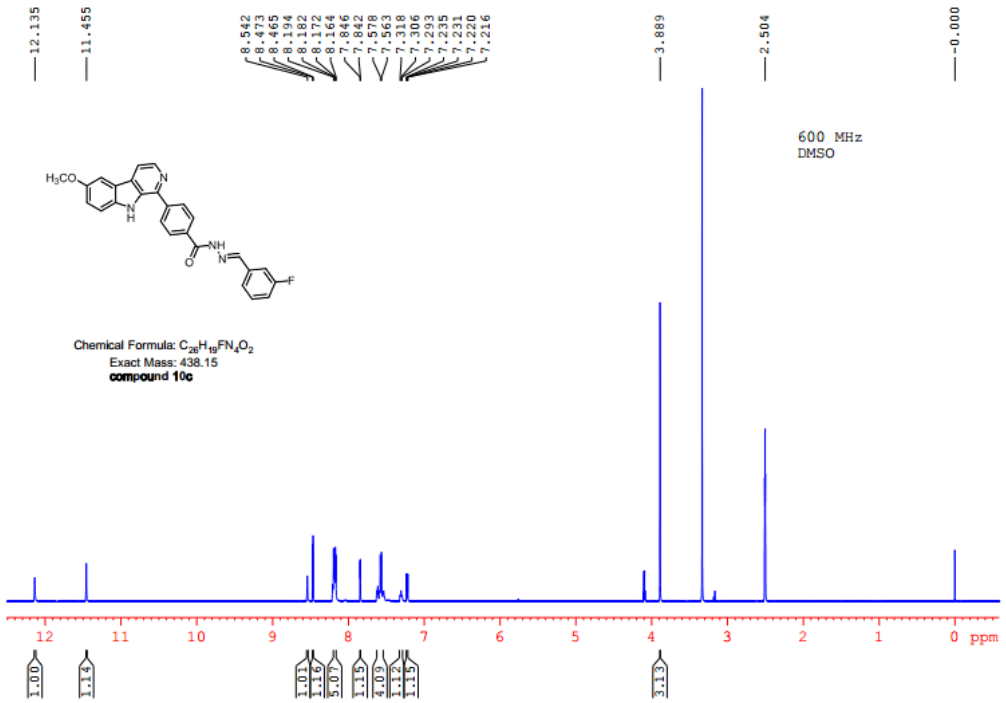

Figure S35 ^{1}H NMR spectrum of **10c**

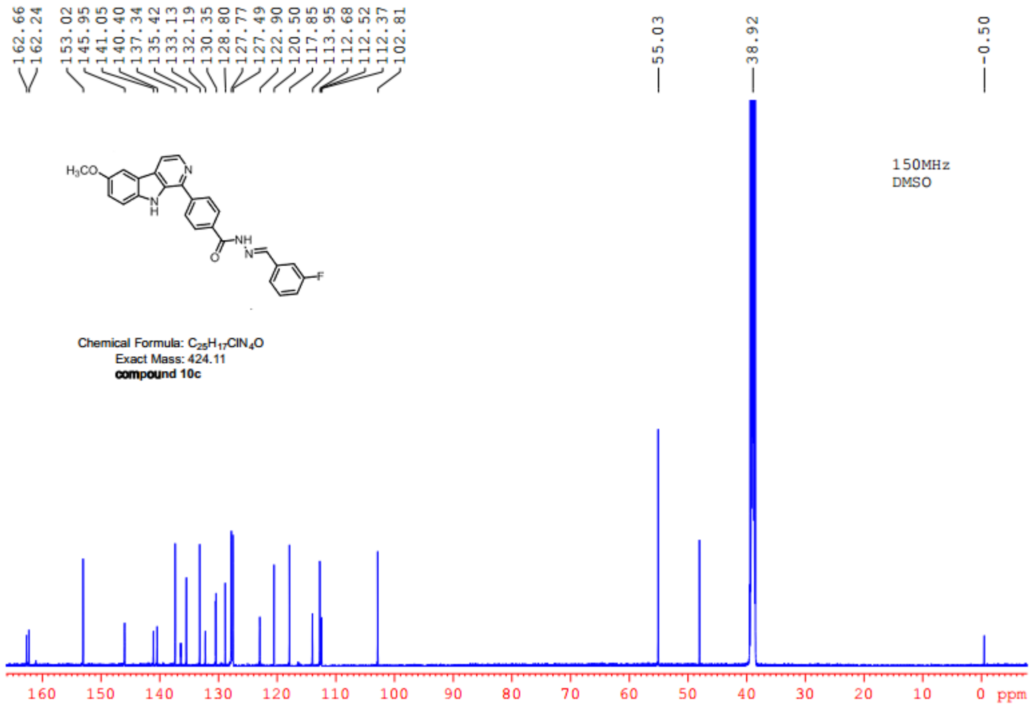

Figure S36 ^{13}C NMR spectrum of **10c**

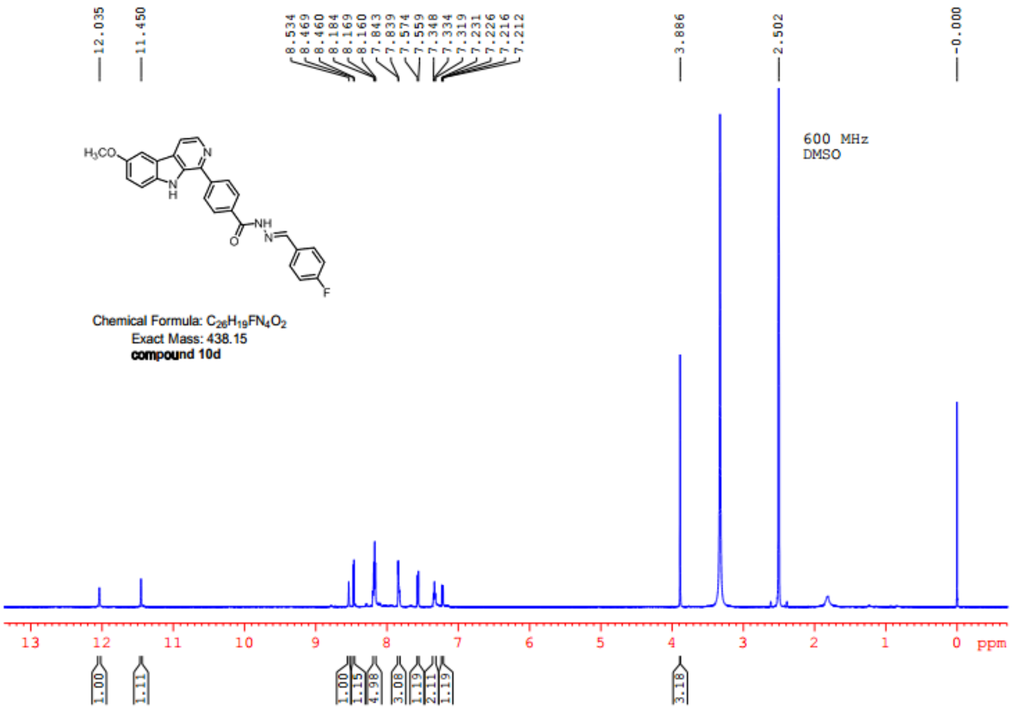

Figure S37 ^{1}H NMR spectrum of **10d**

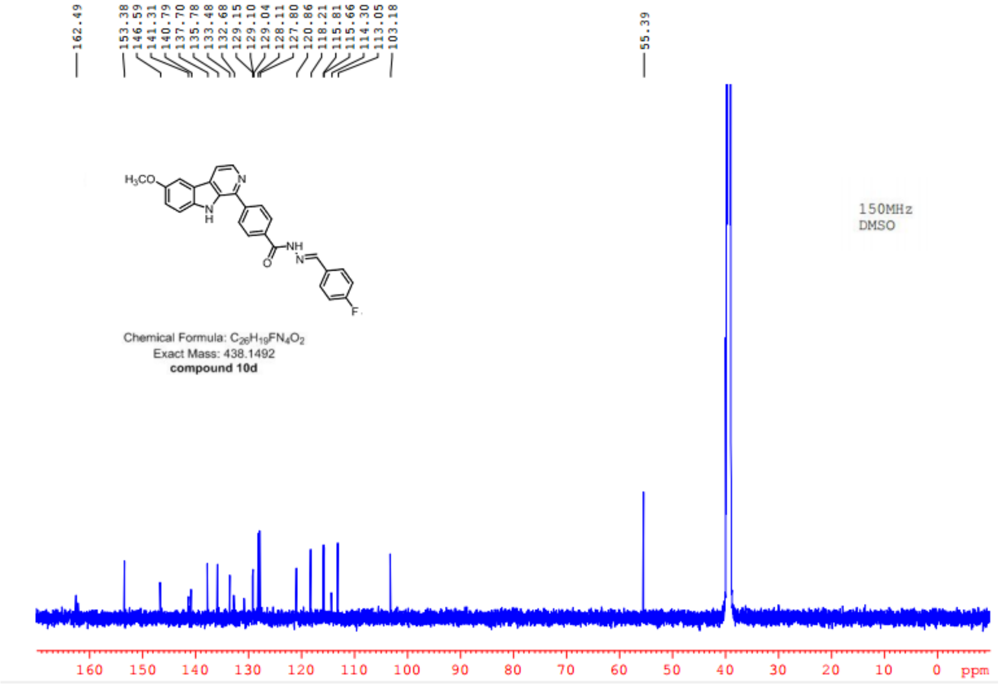

Figure S38 ^{13}C NMR spectrum of **10d**

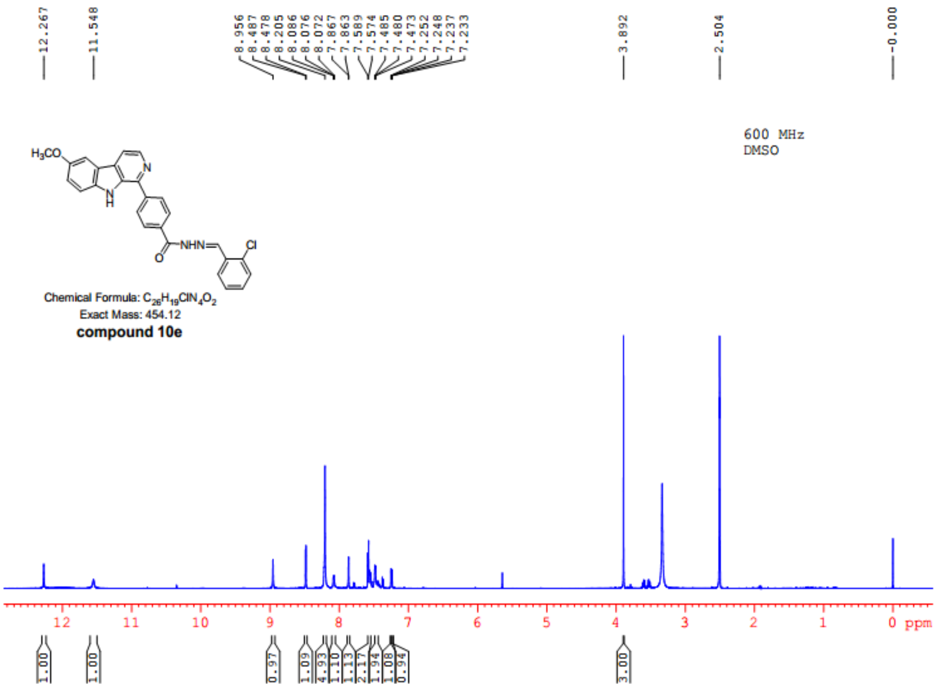

Figure S39 ^{1}H NMR spectrum of **10e**

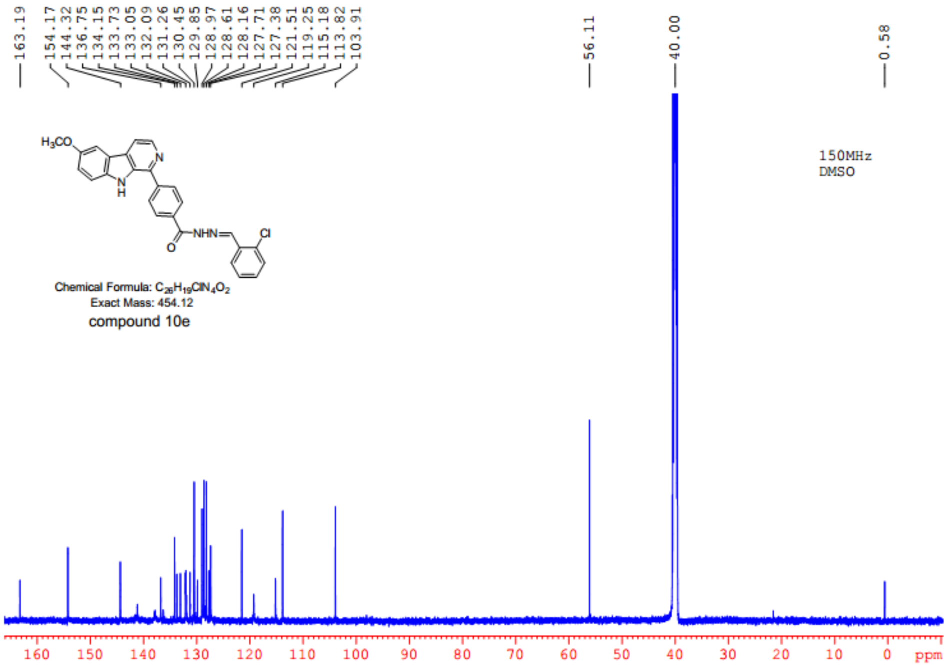

Figure S40 ^{13}C NMR spectrum of **10e**

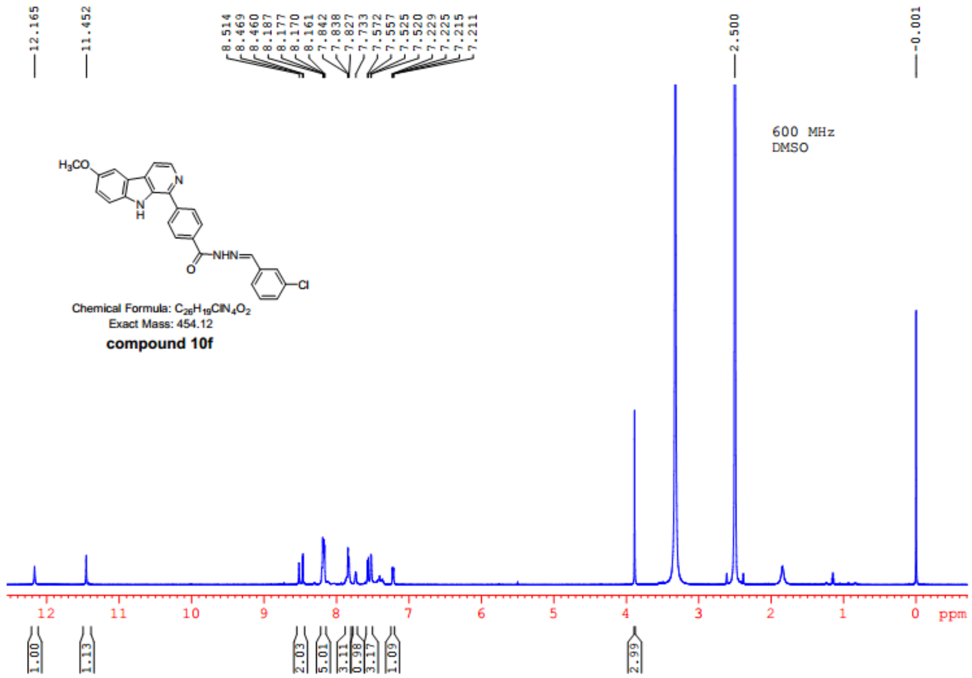

Figure S41 ^{1}H NMR spectrum of **10f**

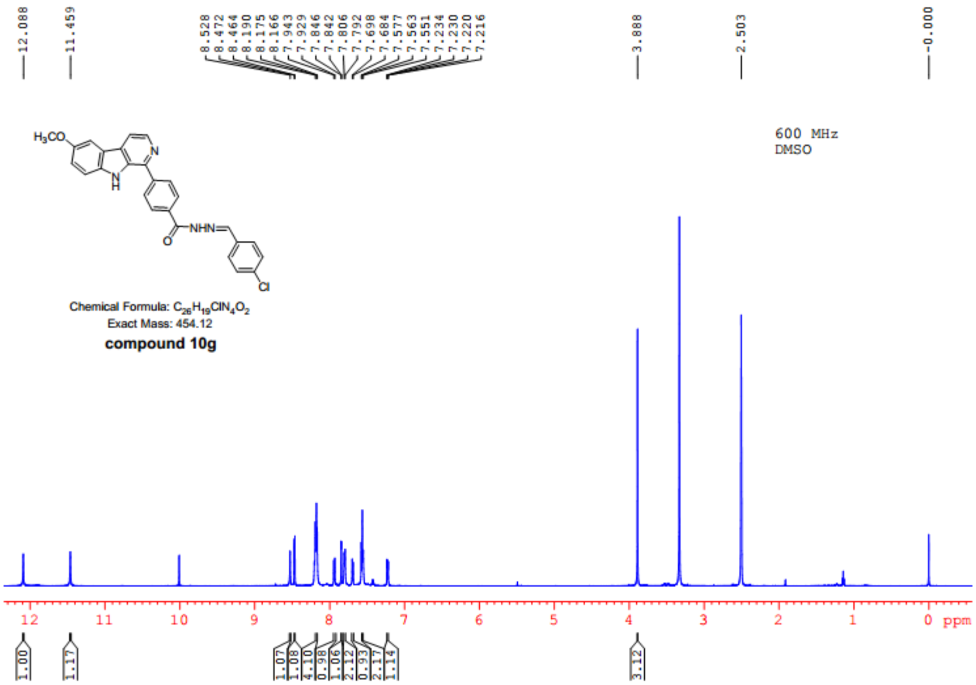

Figure S42 ^{1}H NMR spectrum of **10g**

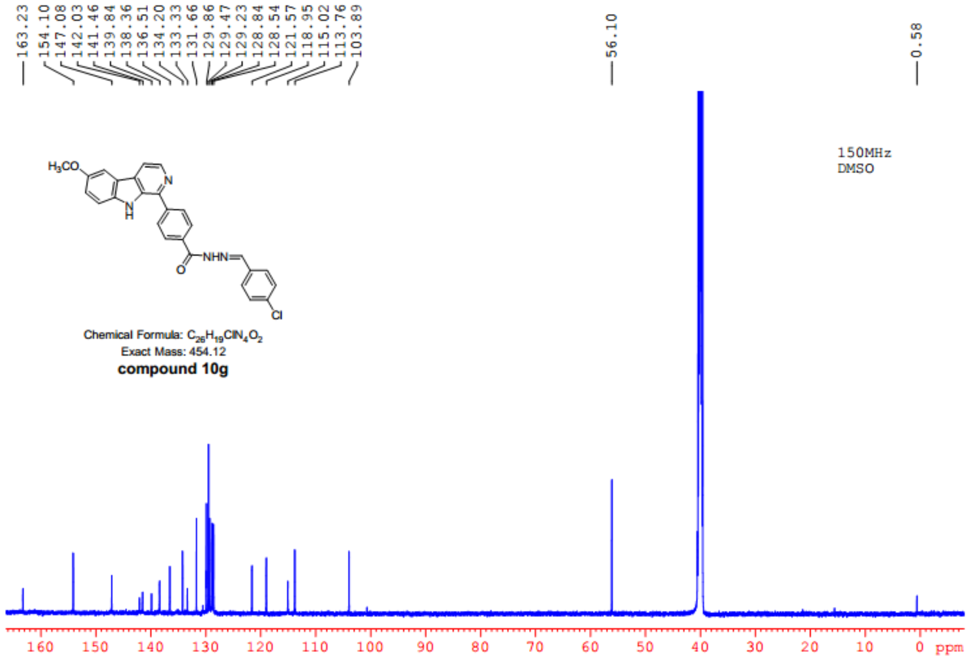

Figure S43 ^{13}C NMR spectrum of **10g**

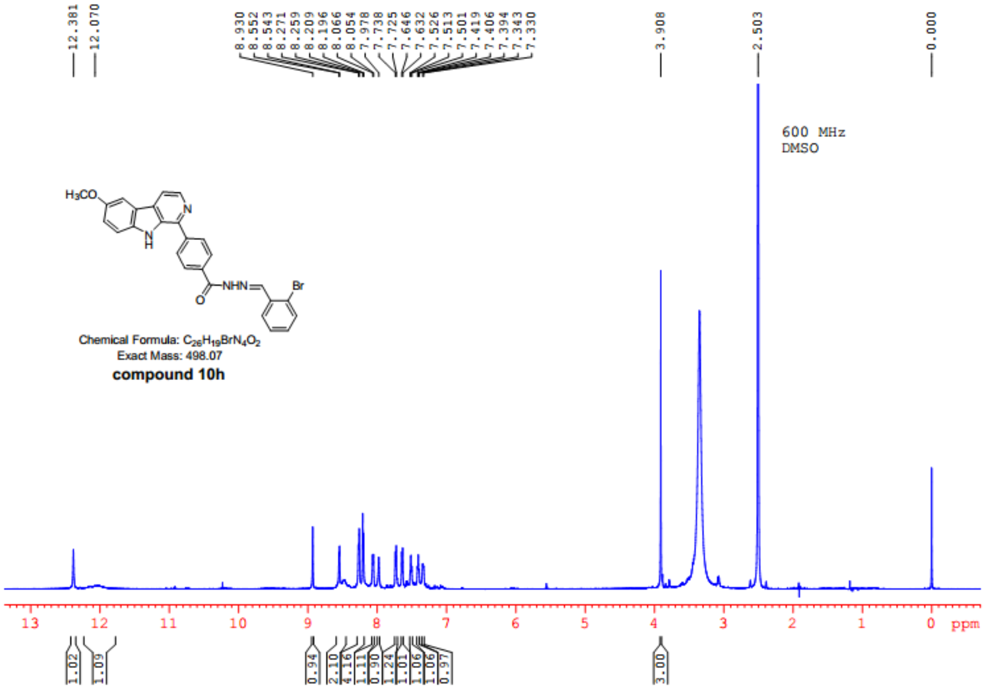

Figure S44 ^{1}H NMR spectrum of **10h**

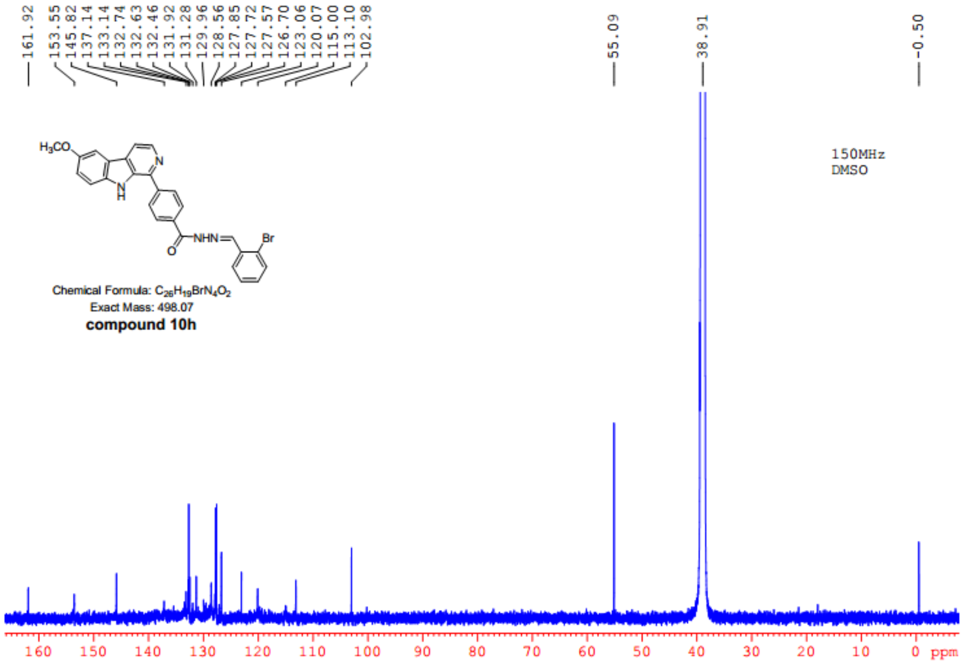

Figure S45 ^{13}C NMR spectrum of **10h**

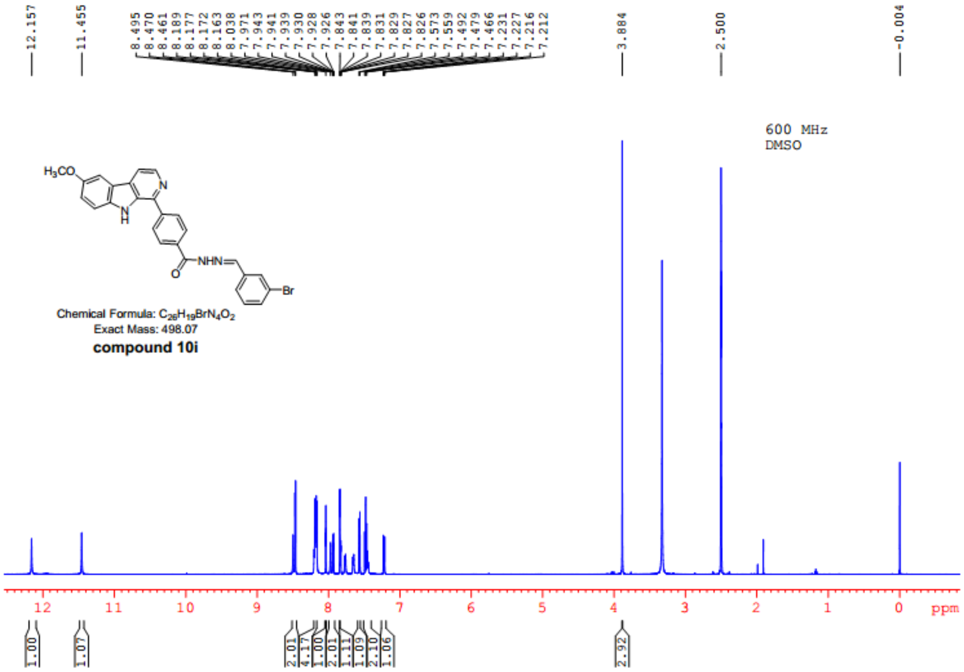

Figure S46 ^{1}H NMR spectrum of **10i**

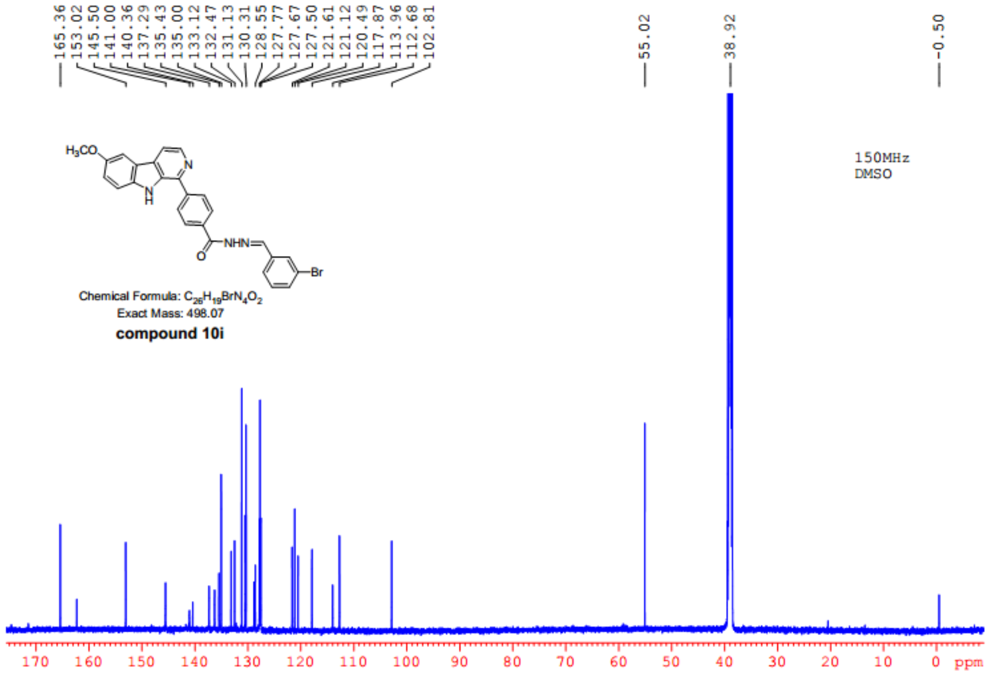

Figure S47 ^{13}C NMR spectrum of **10i**

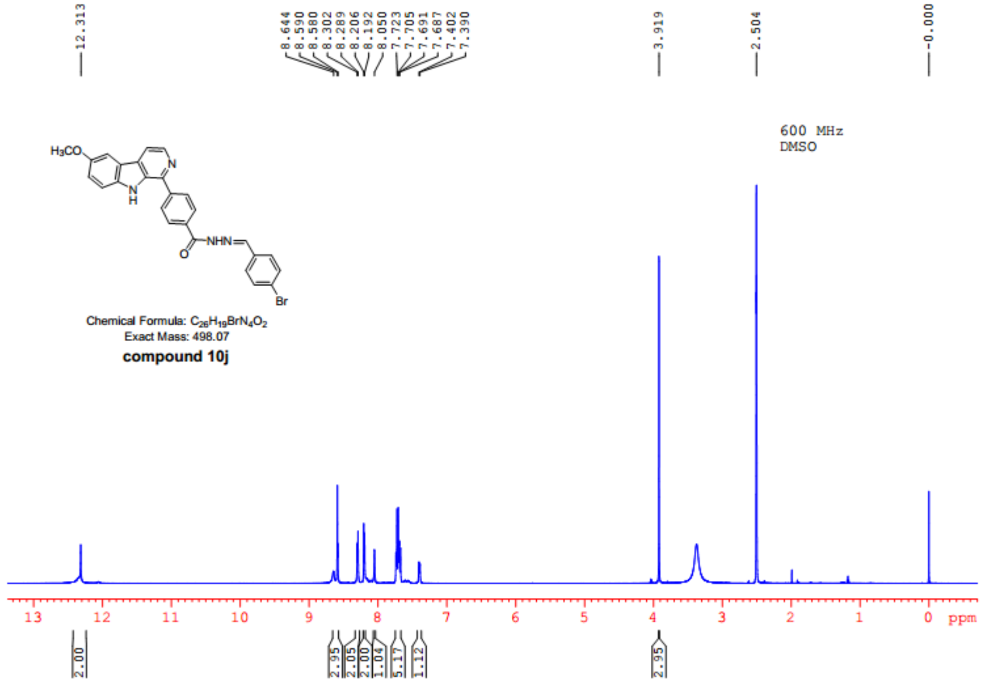

Figure S48 ^{1}H NMR spectrum of **10j**

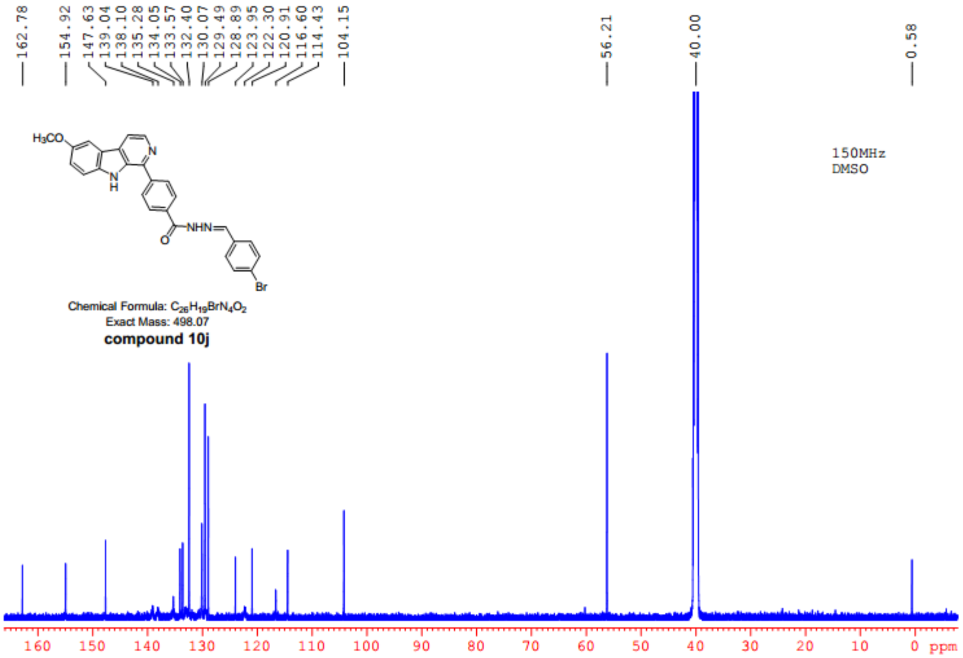

Figure S49 ^{13}C NMR spectrum of **10j**

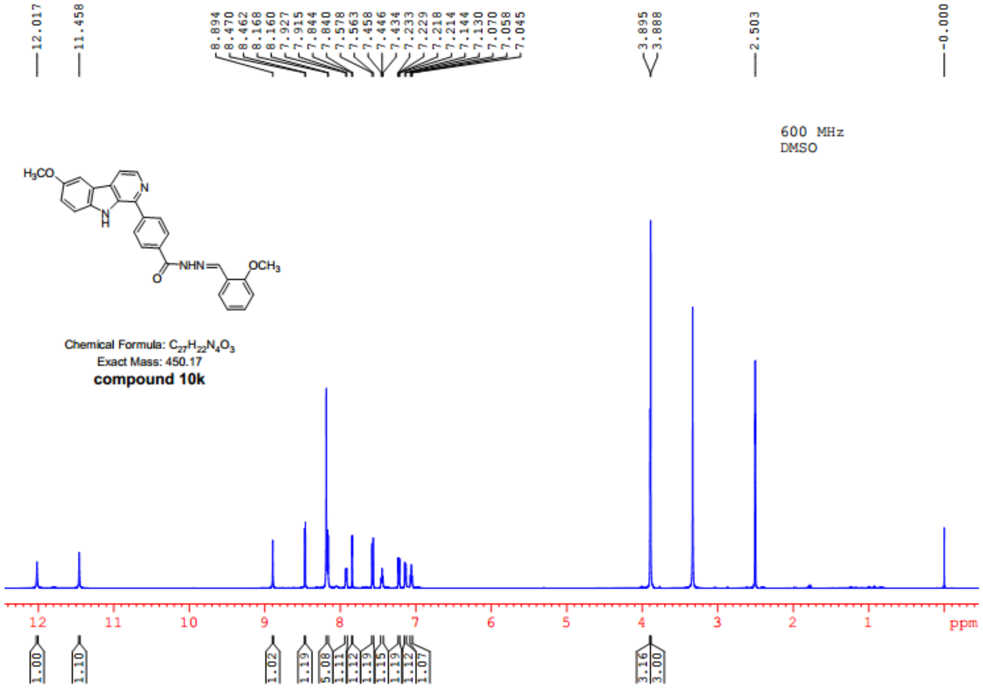

Figure S50 ^{1}H NMR spectrum of **10k**

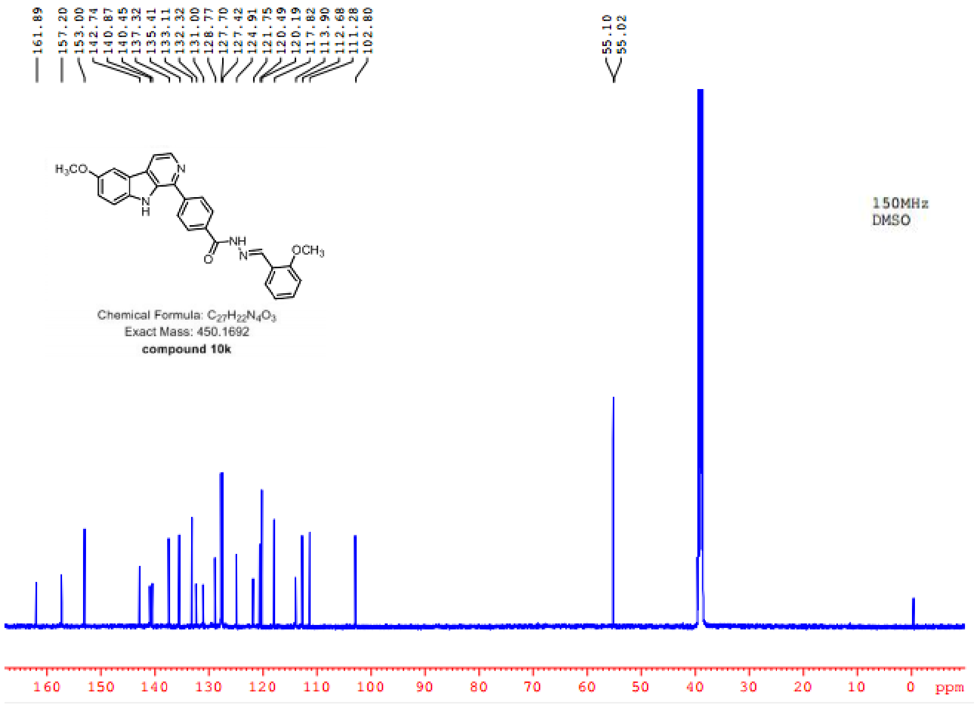

Figure S51 ^{13}C NMR spectrum of **10k**

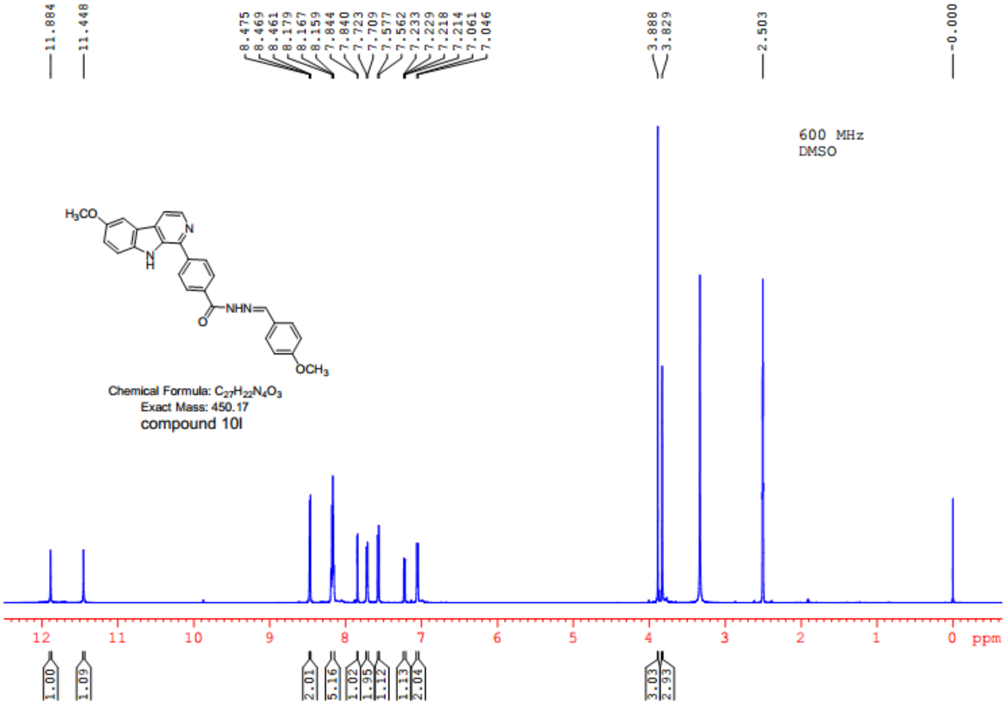

Figure S52 ^{1}H NMR spectrum of **10l**

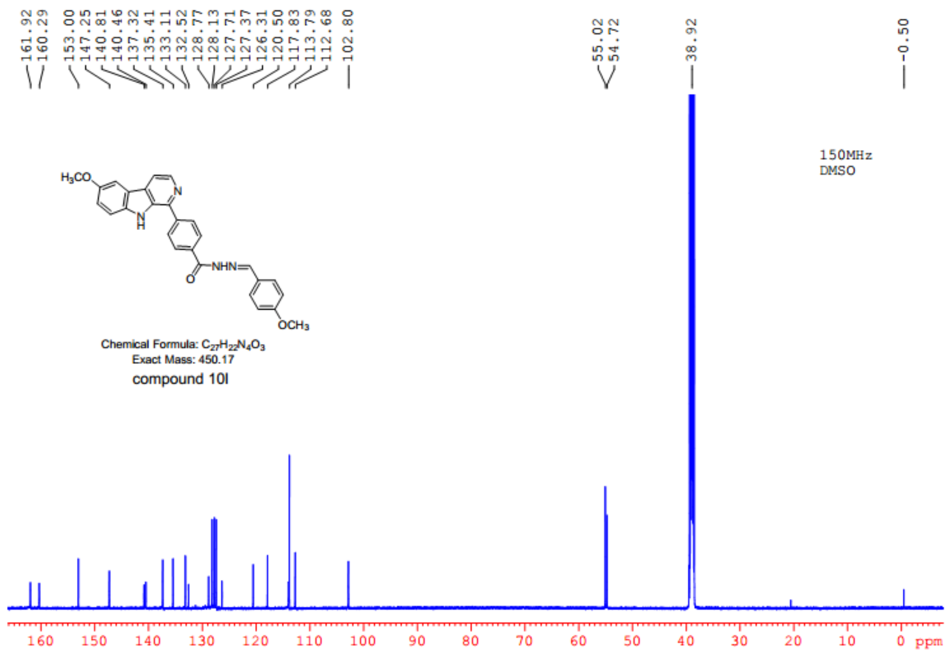

Figure S53 ^{13}C NMR spectrum of **10l**

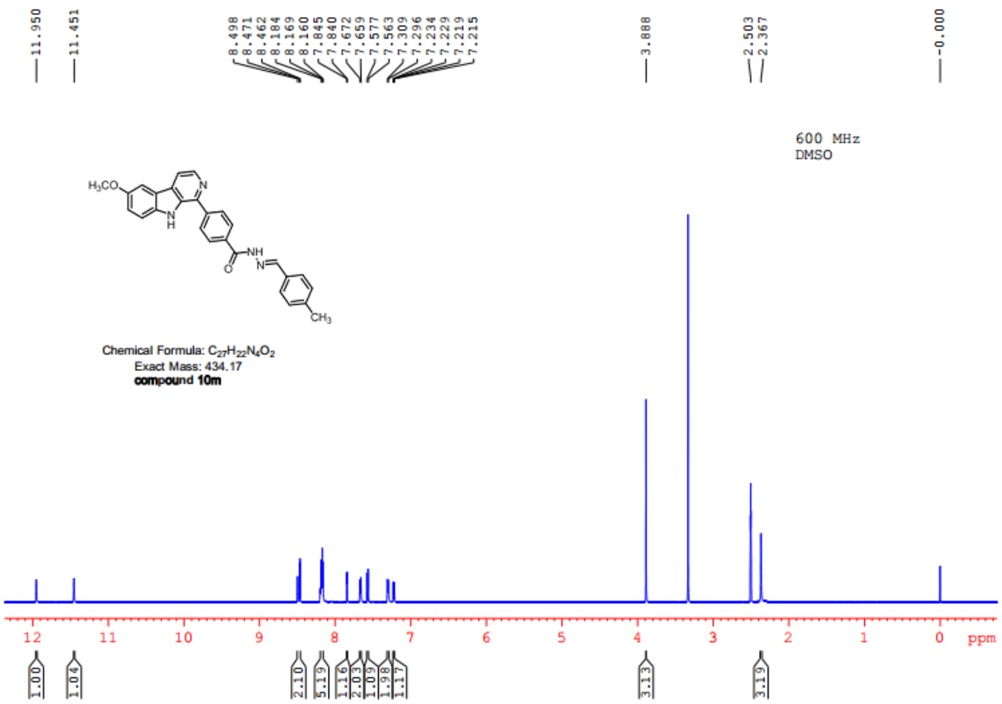

Figure S54 ^{1}H NMR spectrum of **10m**

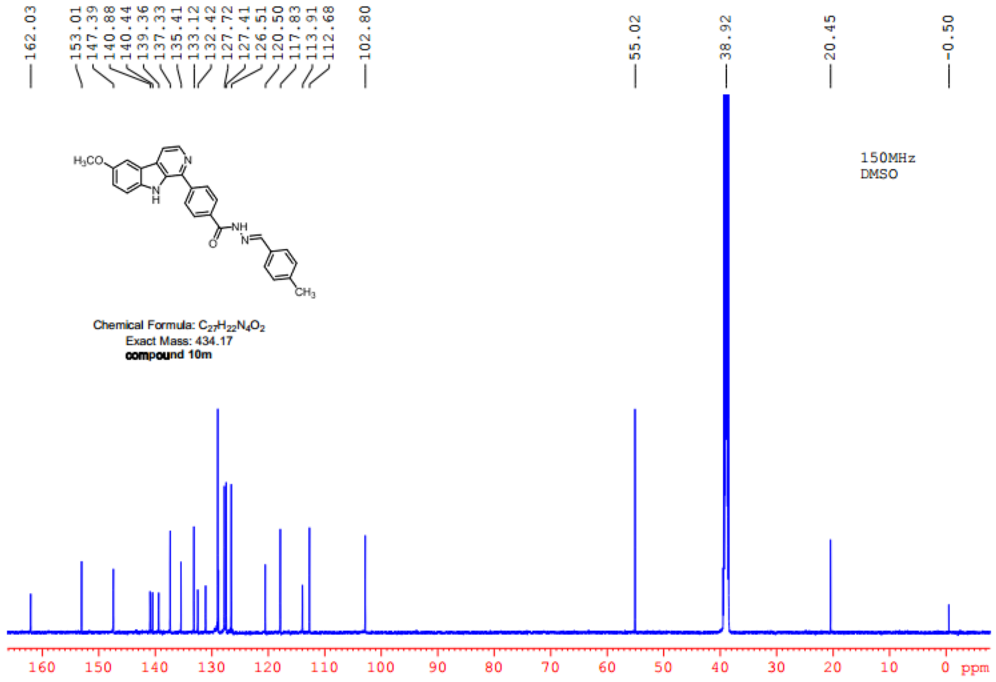

Figure S55 ^{13}C NMR spectrum of **10m**

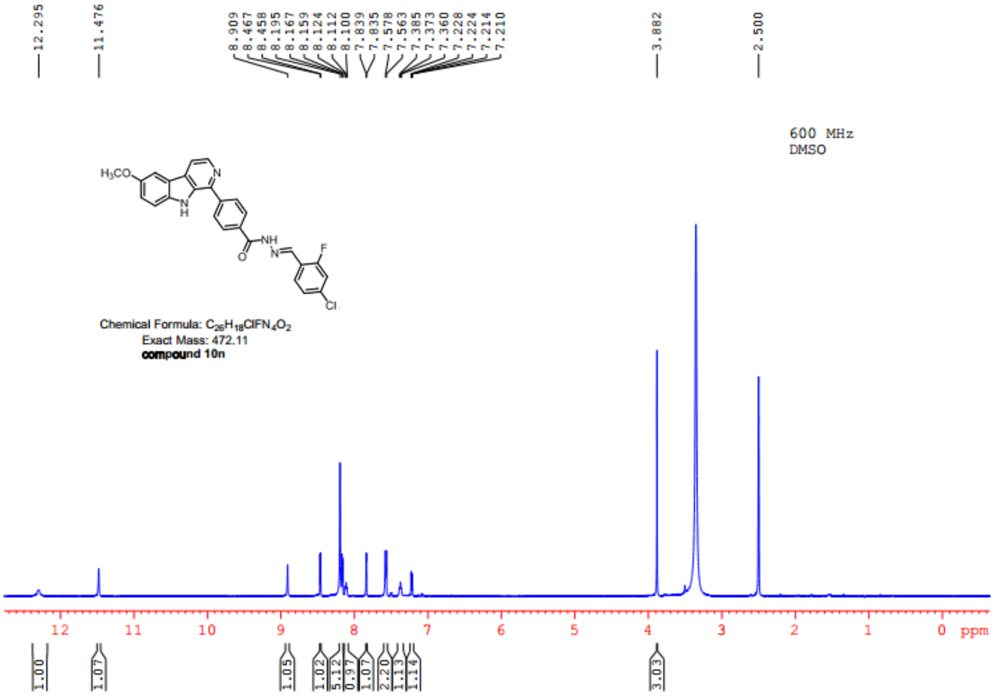

Figure S56 ^{1}H NMR spectrum of **10n**

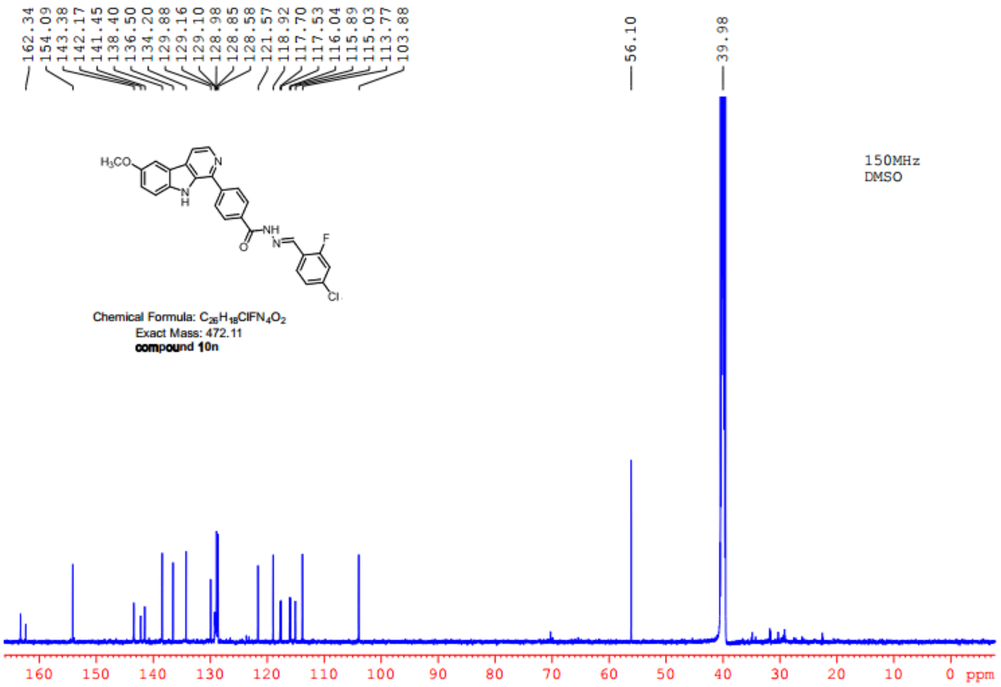

Figure S57 ^{13}C NMR spectrum of **10n**

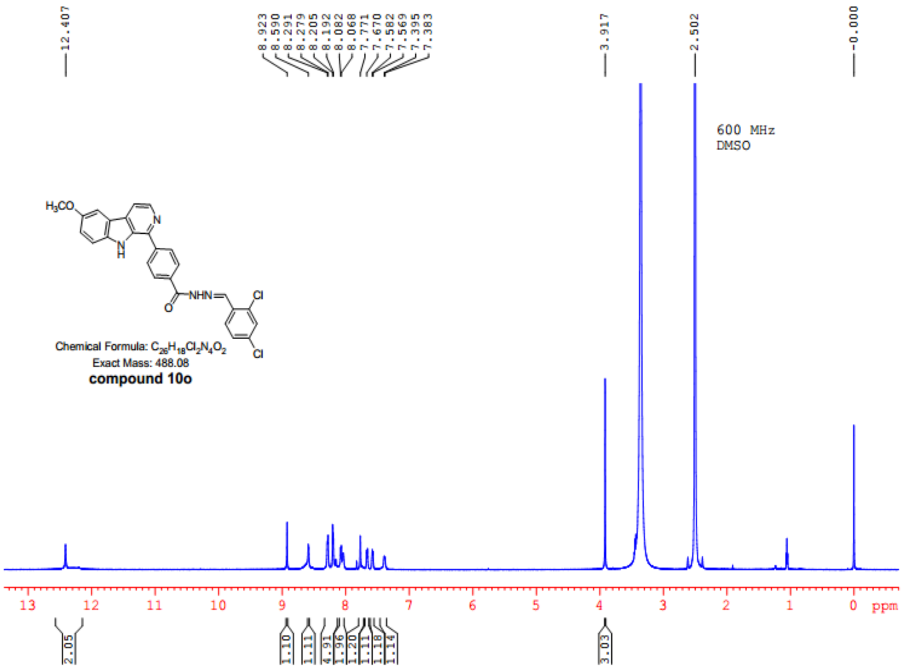

Figure S58 ^{1}H NMR spectrum of **10o**

3. The HRMS spectra of compounds 9a-o and 10a-o.

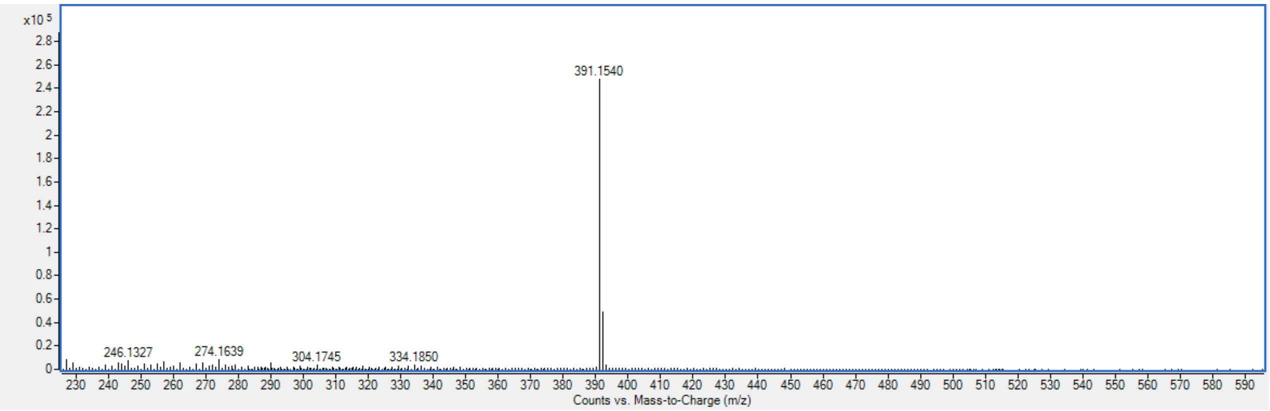

Figure S59 HRMS spectra of **9a**

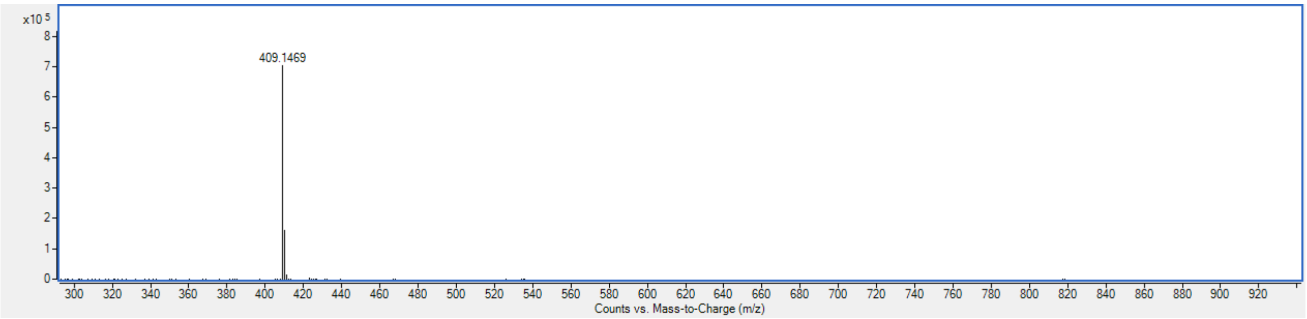

Figure S60 HRMS spectra of **9b**

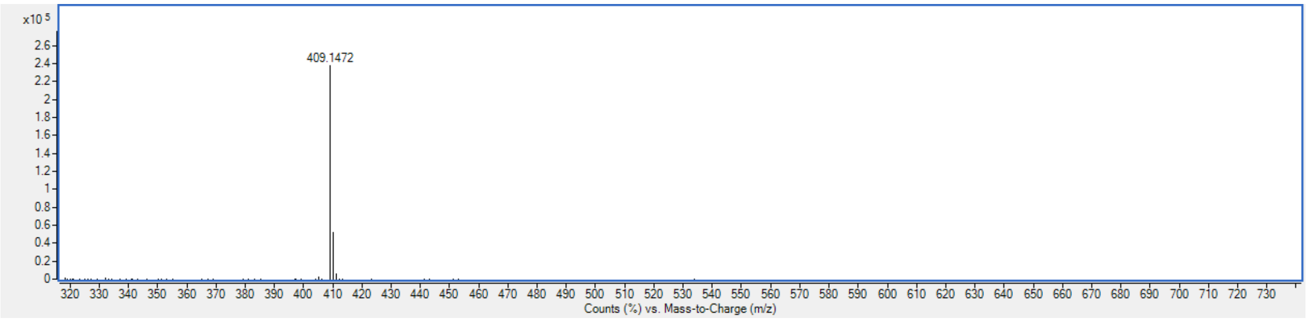

Figure S61 HRMS spectra of **9c**

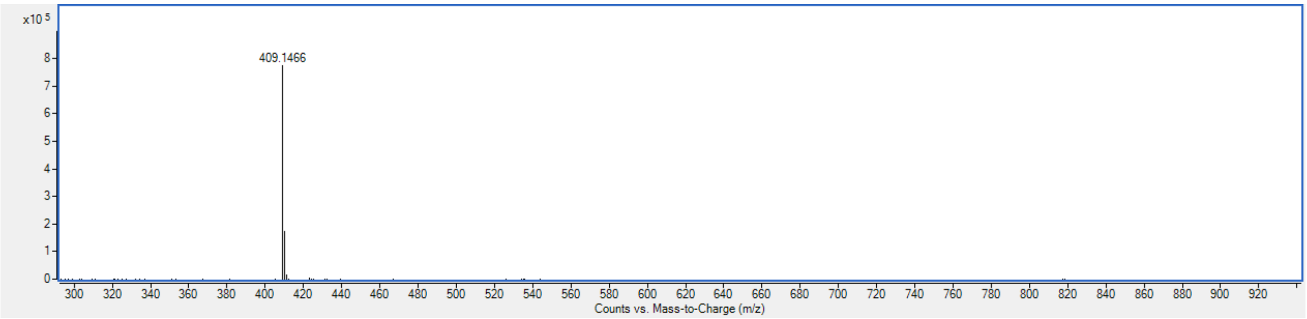

Figure S62 HRMS spectra of **9d**

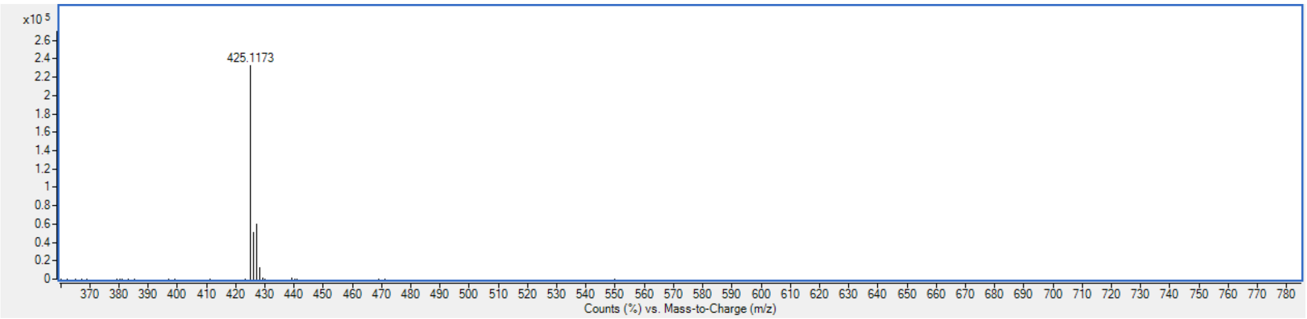

Figure S63 HRMS spectra of **9e**

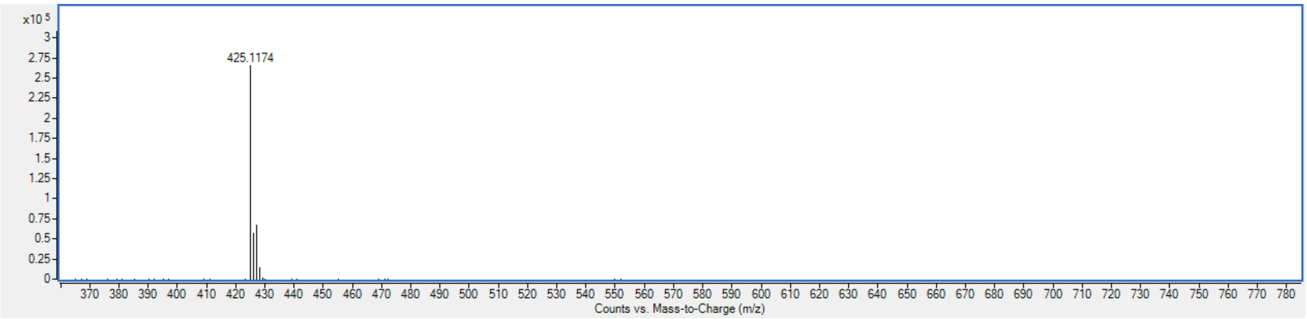

Figure S64 HRMS spectra of **9f**

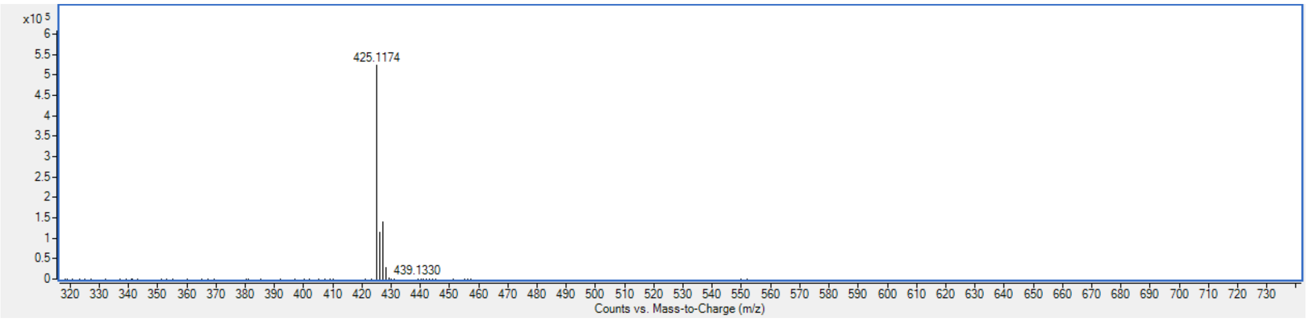

Figure S65 HRMS spectra of **9g**

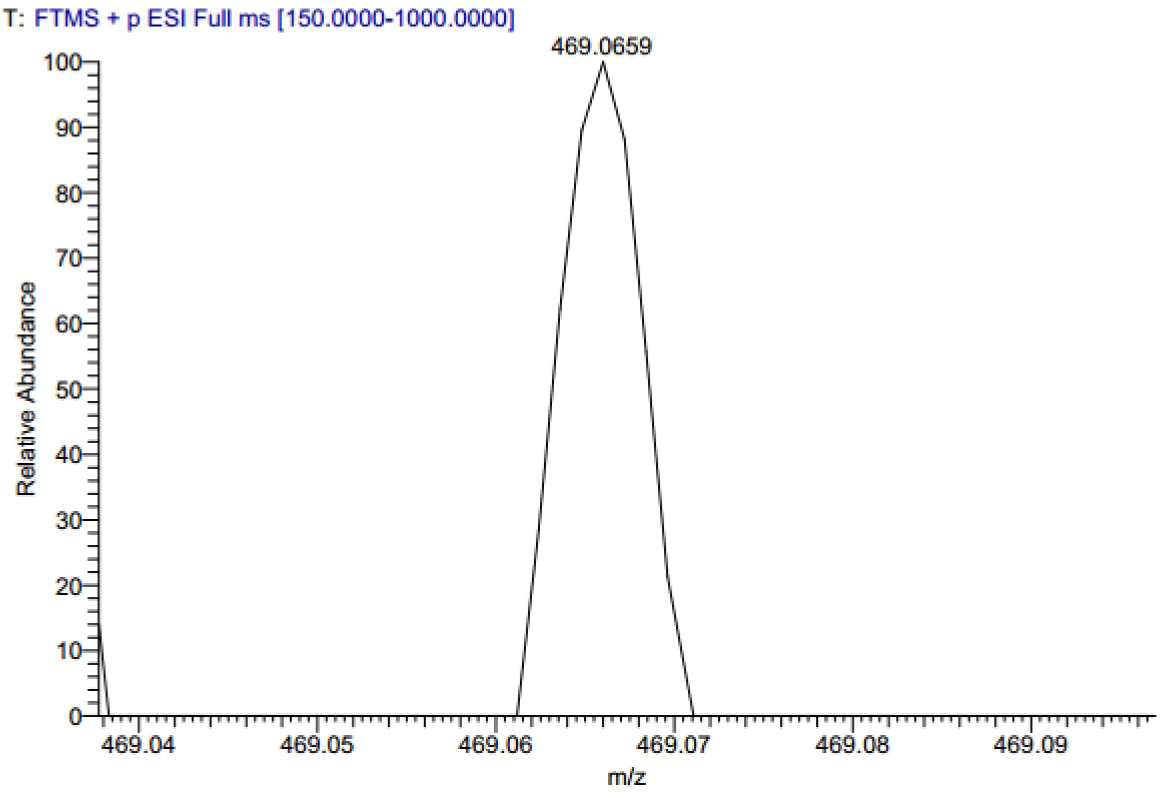

Figure S66 HRMS spectra of **9h**

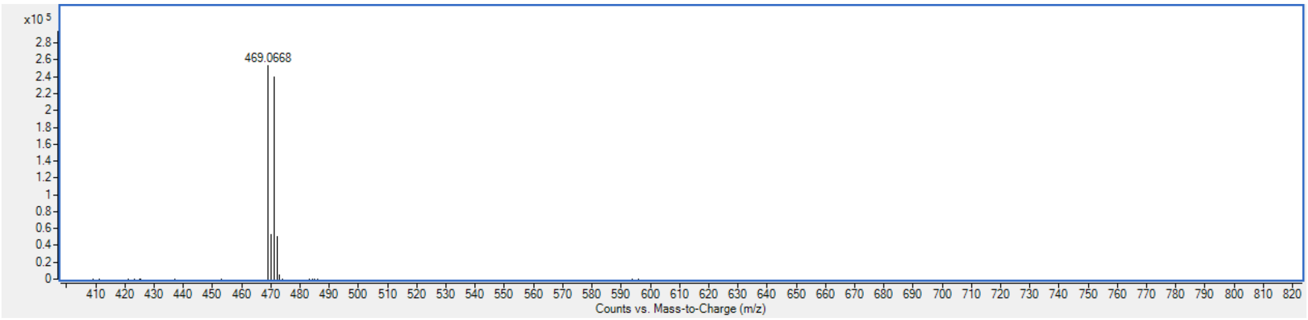

Figure S67 HRMS spectra of **9i**

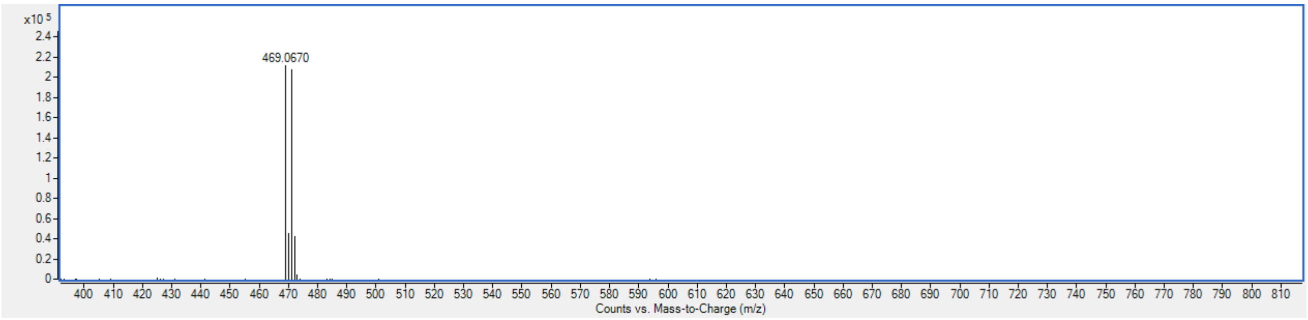

Figure S68 HRMS spectra of **9j**

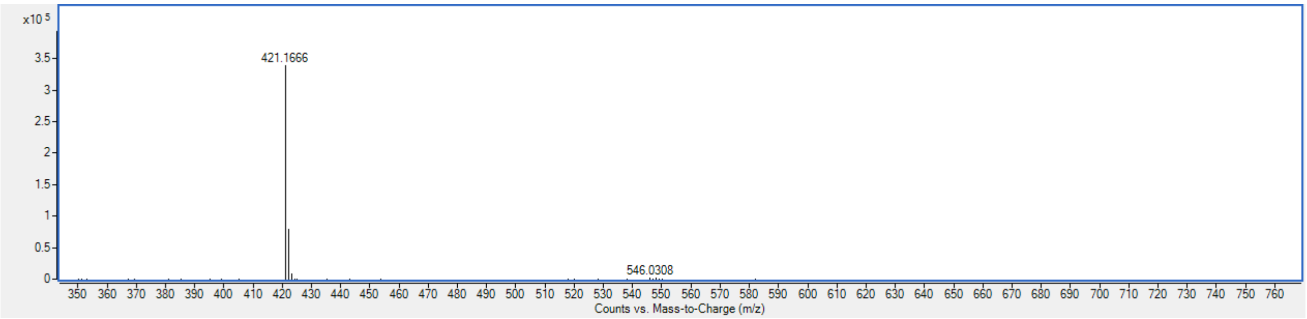

Figure S69 HRMS spectra of **9k**

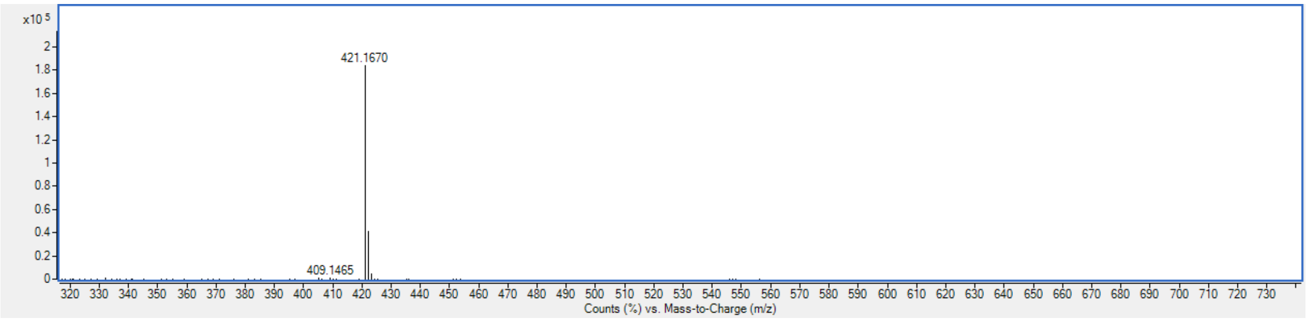

Figure S70 HRMS spectra of **9l**

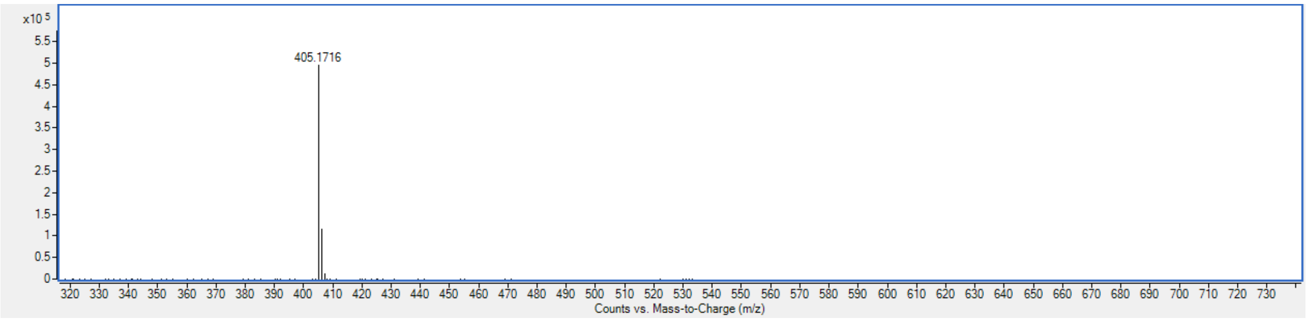

Figure S71 HRMS spectra of **9m**

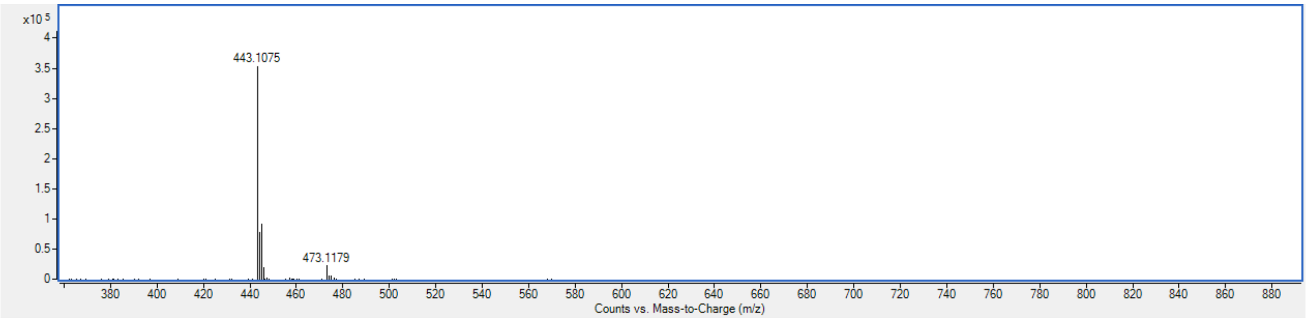

Figure S72 HRMS spectra of **9n**

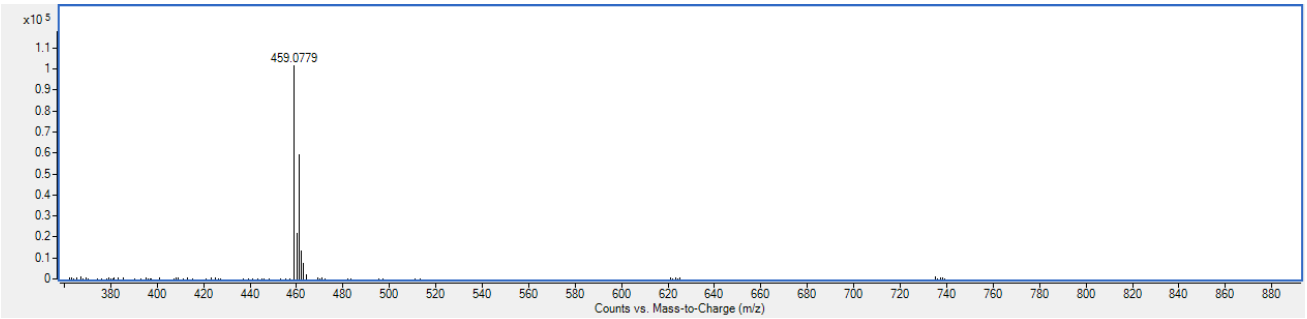

Figure S73 HRMS spectra of **9o**

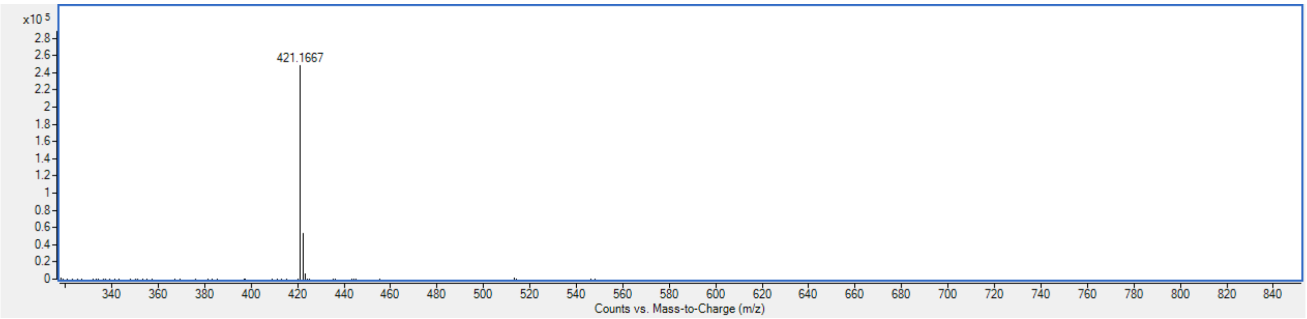

Figure S74 HRMS spectra of **10a**

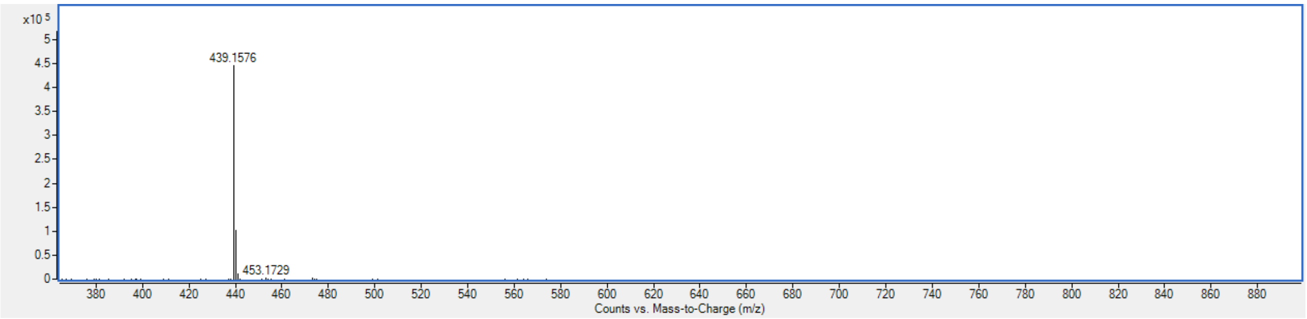

Figure S75 HRMS spectra of **10b**

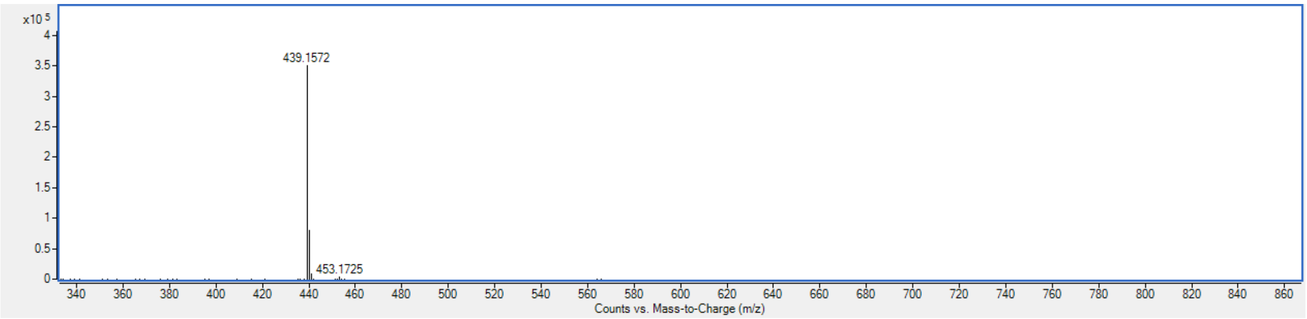

Figure S76 HRMS spectra of **10c**

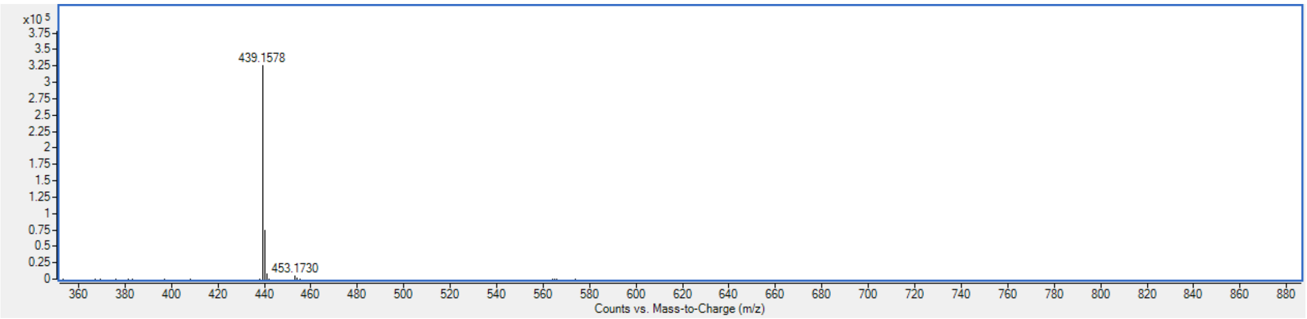

Figure S77 HRMS spectra of **10d**

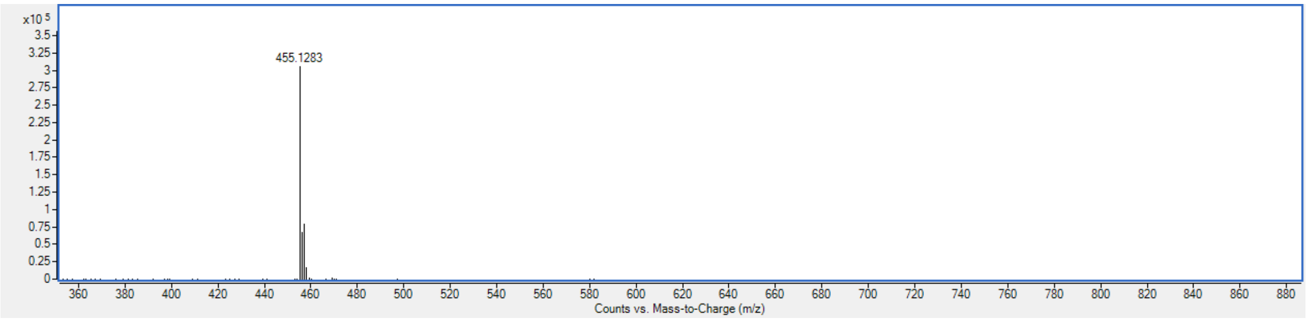

Figure S78 HRMS spectra of **10e**

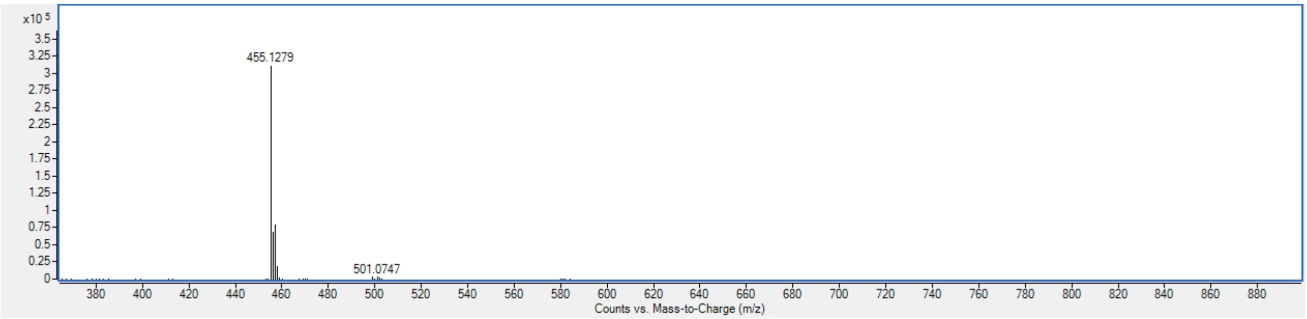

Figure S79 HRMS spectra of **10f**

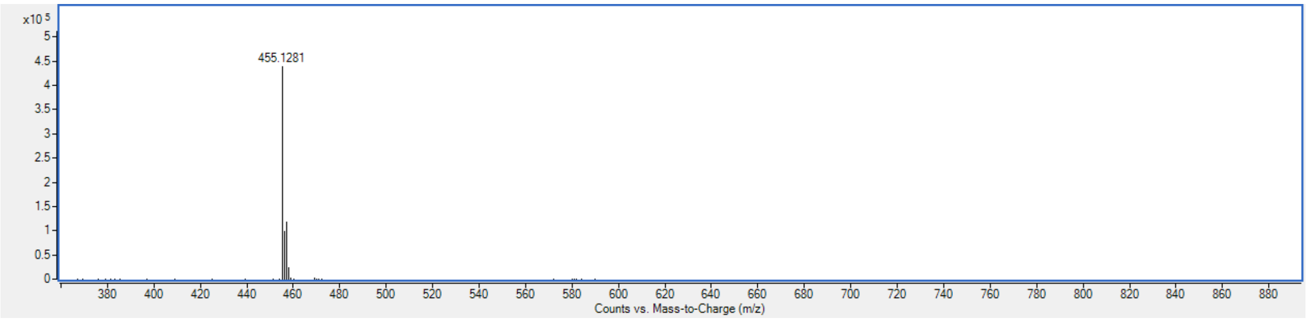

Figure S80 HRMS spectra of **10g**

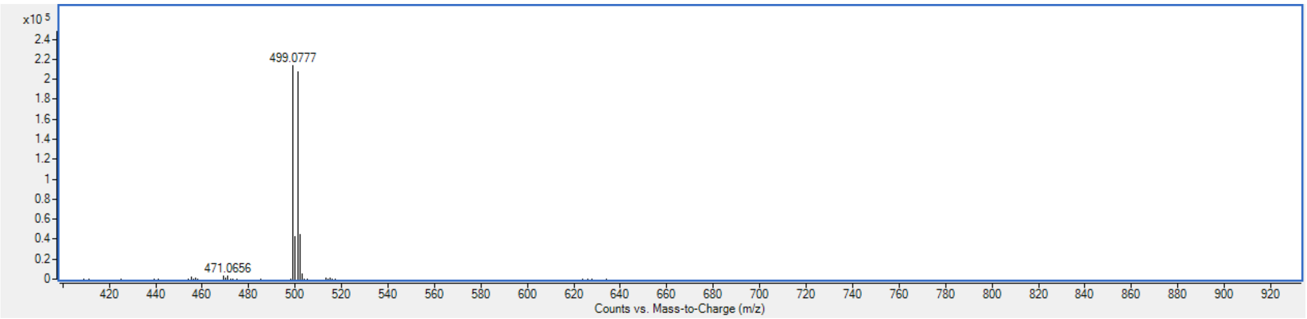

Figure S81 HRMS spectra of **10h**

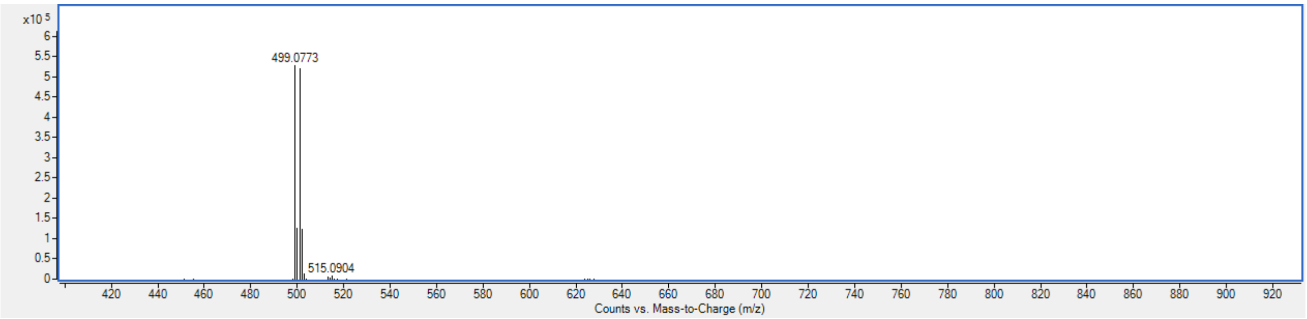

Figure S82 HRMS spectra of **10i**

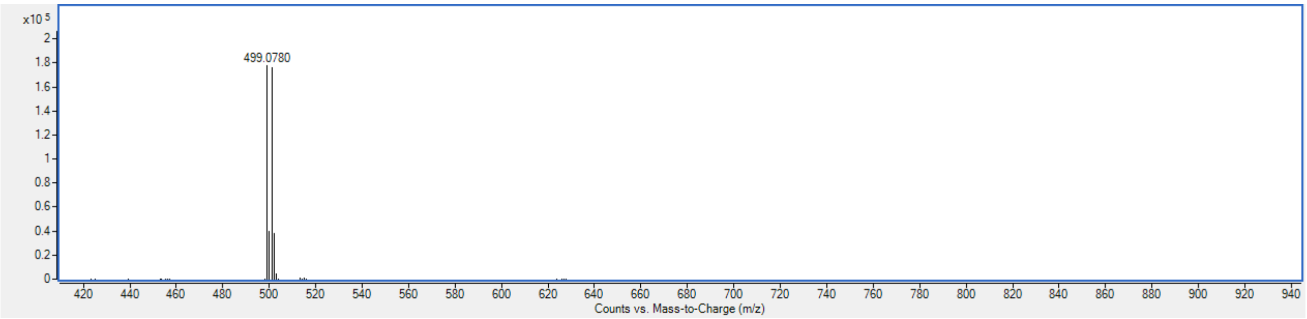

Figure S83 HRMS spectra of **10j**

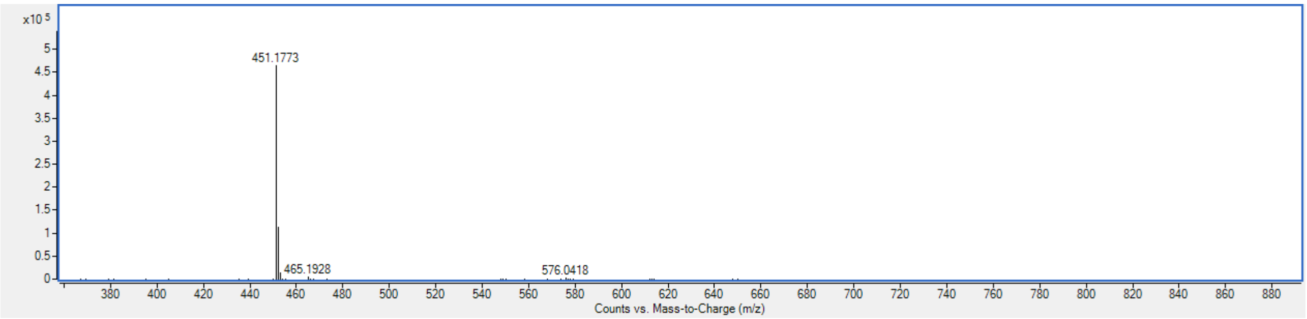

Figure S84 HRMS spectra of **10k**

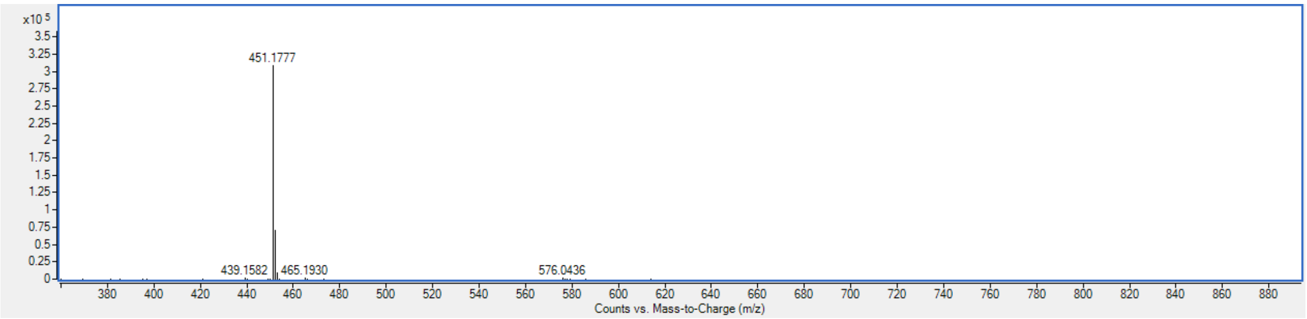

Figure S85 HRMS spectra of **10l**

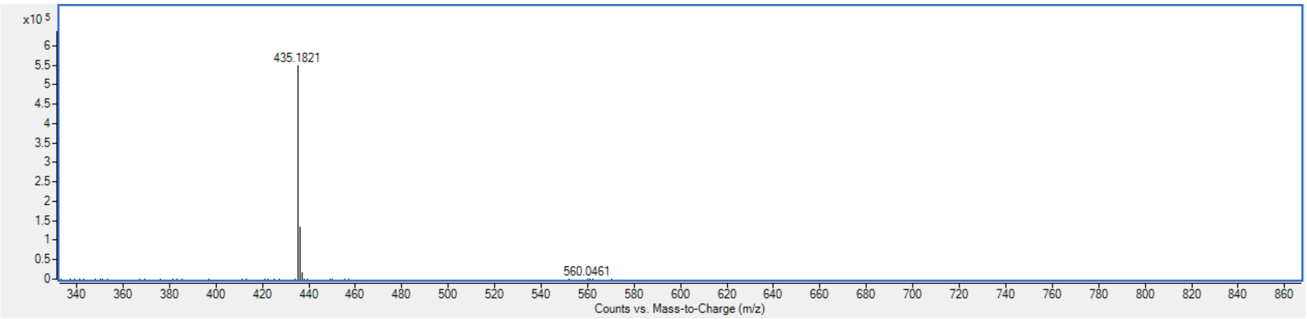

Figure S86 HRMS spectra of **10m**

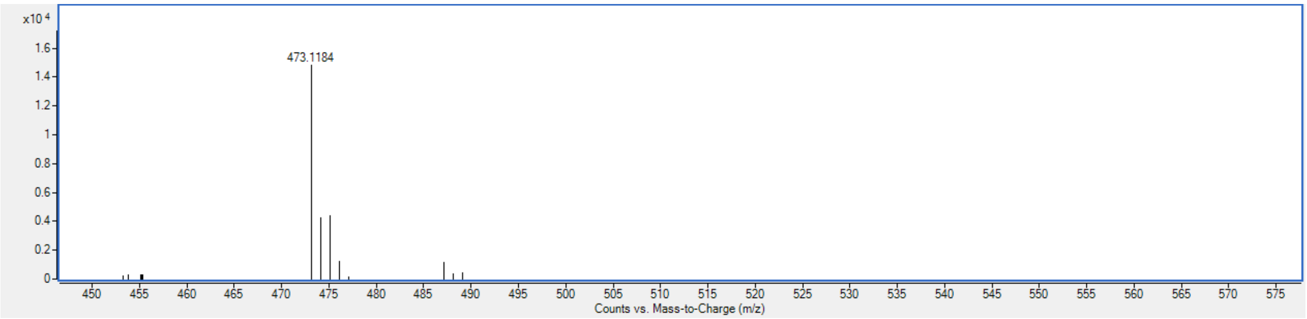

Figure S87 HRMS spectra of **10n**

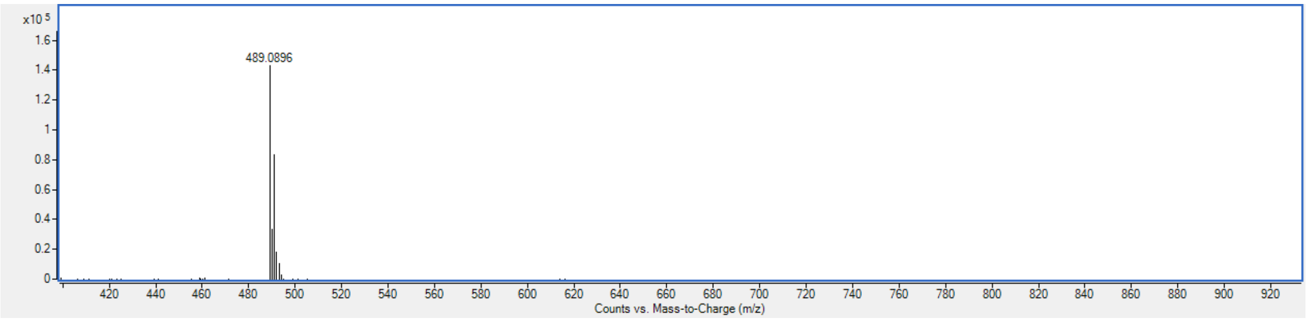

Figure S88 HRMS spectra of **10º**

4. The toxicity regression equation of 9n, 9o and hymexazol.

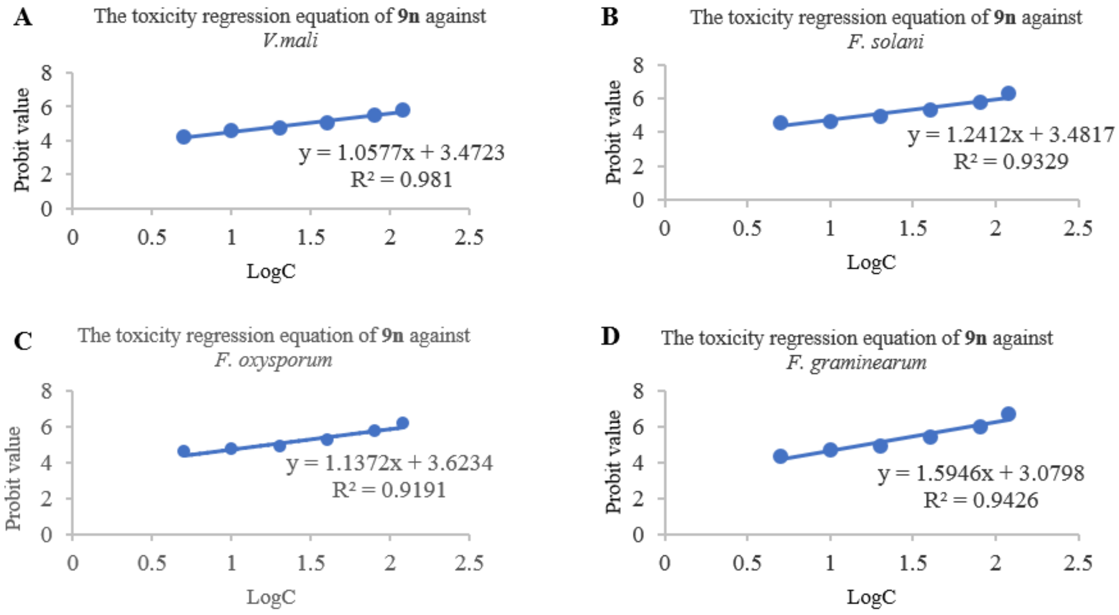

Figure S89 The toxicity regression equation of **9n**

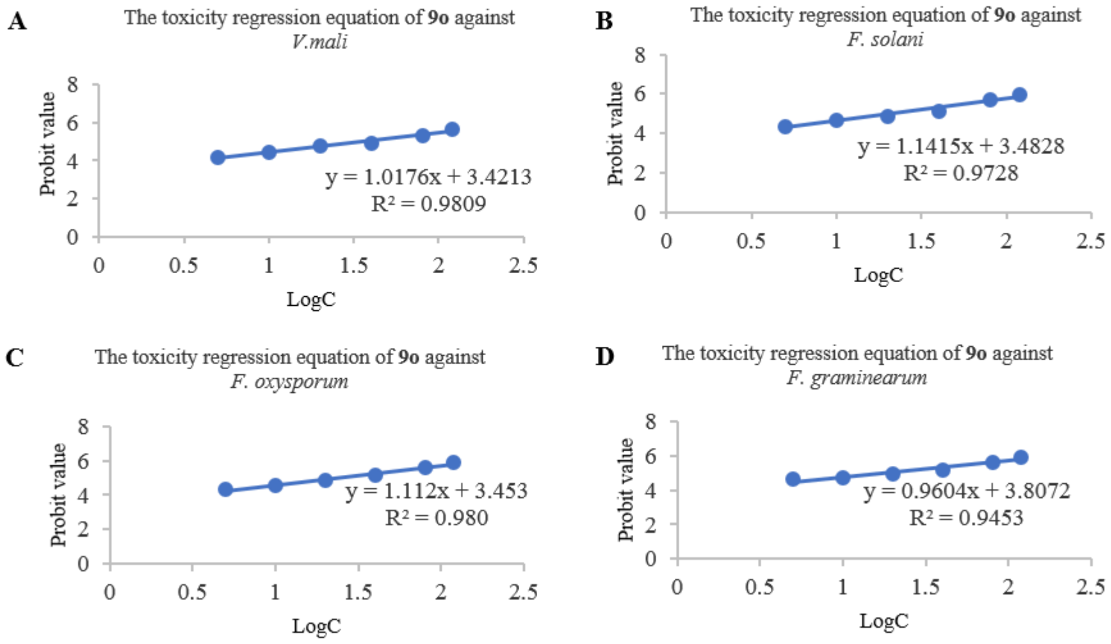

Figure S90 The toxicity regression equation of **9o**

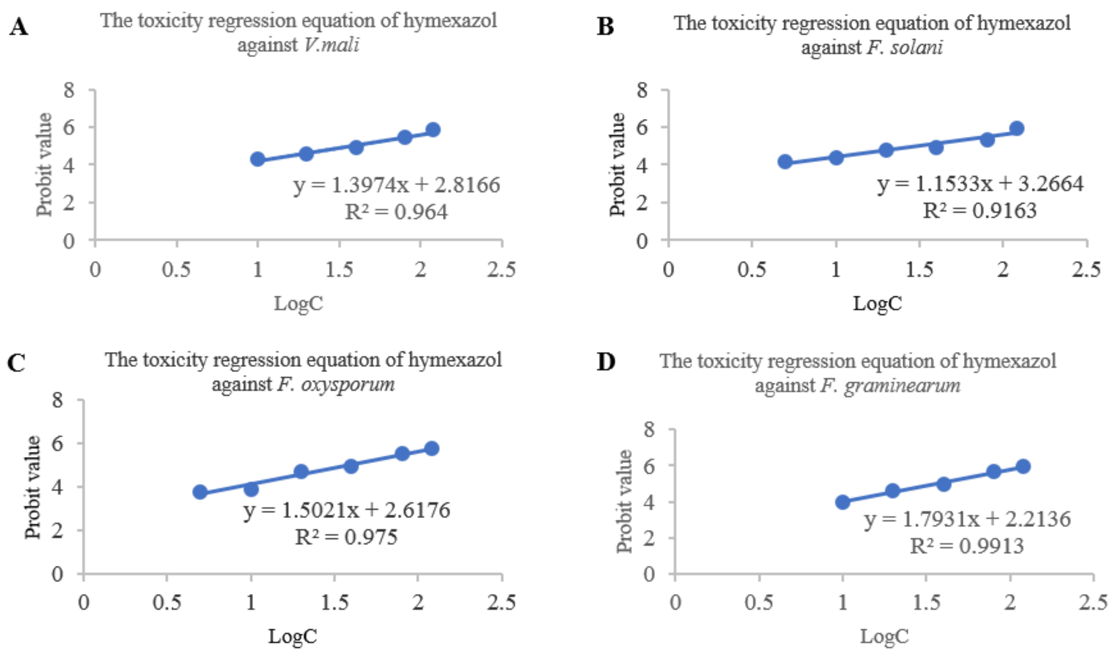

Figure S91 The toxicity regression equation of hymexazol
